# Cardiac Magnetic Resonance Radiomics Reveal Differential Impact of Sex, Age, and Vascular Risk Factors on Cardiac Structure and Myocardial Tissue

**DOI:** 10.3389/fcvm.2021.763361

**Published:** 2021-12-22

**Authors:** Zahra Raisi-Estabragh, Akshay Jaggi, Polyxeni Gkontra, Celeste McCracken, Nay Aung, Patricia B. Munroe, Stefan Neubauer, Nicholas C. Harvey, Karim Lekadir, Steffen E. Petersen

**Affiliations:** ^1^National Institute for Health Research (NIHR) Barts Biomedical Research Centre, William Harvey Research Institute, Queen Mary University of London, London, United Kingdom; ^2^Barts Health National Health Service (NHS) Trust, Barts Heart Centre, St Bartholomew's Hospital, West Smithfield, United Kingdom; ^3^Departament de Matemàtiques and Informàtica, Universitat de Barcelona, Barcelona, Spain; ^4^Division of Cardiovascular Medicine, Radcliffe Department of Medicine, University of Oxford, National Institute for Health Research Oxford Biomedical Research Centre, Oxford University Hospitals NHS Foundation Trust, Oxford, United Kingdom; ^5^Medical Research Council (MRC) Lifecourse Epidemiology Centre, University of Southampton, Southampton, United Kingdom; ^6^NIHR Southampton Biomedical Research Centre, University of Southampton and University Hospital Southampton NHS Foundation Trust, Southampton, United Kingdom; ^7^Health Data Research UK, London, United Kingdom; ^8^Alan Turing Institute, London, United Kingdom

**Keywords:** cardiovascular magnetic resonance, radiomics, healthy individuals, diabetes, hypertension, high cholesterol, smoking, sex differences

## Abstract

**Background:** Cardiovascular magnetic resonance (CMR) radiomics analysis provides multiple quantifiers of ventricular shape and myocardial texture, which may be used for detailed cardiovascular phenotyping.

**Objectives:** We studied variation in CMR radiomics phenotypes by age and sex in healthy UK Biobank participants. Then, we examined independent associations of classical vascular risk factors (VRFs: smoking, diabetes, hypertension, high cholesterol) with CMR radiomics features, considering potential sex and age differential relationships.

**Design:** Image acquisition was with 1.5 Tesla scanners (MAGNETOM Aera, Siemens). Three regions of interest were segmented from short axis stack images using an automated pipeline: right ventricle, left ventricle, myocardium. We extracted 237 radiomics features from each study using Pyradiomics. In a healthy subset of participants (*n* = 14,902) without cardiovascular disease or VRFs, we estimated independent associations of age and sex with each radiomics feature using linear regression models adjusted for body size. We then created a sample comprising individuals with at least one VRF matched to an equal number of healthy participants (*n* = 27,400). We linearly modelled each radiomics feature against age, sex, body size, and all the VRFs. Bonferroni adjustment for multiple testing was applied to all *p*-values. To aid interpretation, we organised the results into six feature clusters.

**Results:** Amongst the healthy subset, men had larger ventricles with dimmer and less texturally complex myocardium than women. Increasing age was associated with smaller ventricles and greater variation in myocardial intensities. Broadly, all the VRFs were associated with dimmer, less varied signal intensities, greater uniformity of local intensity levels, and greater relative presence of low signal intensity areas within the myocardium. Diabetes and high cholesterol were also associated with smaller ventricular size, this association was of greater magnitude in men than women. The pattern of alteration of radiomics features with the VRFs was broadly consistent in men and women. However, the associations between intensity based radiomics features with both diabetes and hypertension were more prominent in women than men.

**Conclusions:** We demonstrate novel independent associations of sex, age, and major VRFs with CMR radiomics phenotypes. Further studies into the nature and clinical significance of these phenotypes are needed.

## Introduction

Epidemiologic studies highlight cigarette smoking, high blood pressure, and high cholesterol as major modifiable risk factors for cardiovascular disease (1, 2). The association of these risk factors with incident cardiovascular events has been widely reported in multiple settings and their modification linked to substantial reductions in cardiovascular mortality (2).

There are important heterogeneities in cardiovascular disease patterns and clinical outcomes between men and women (3, 4). These differences may be partly explained by differential biological consequences of vascular risk factors (5, 6). Existing studies using cardiovascular magnetic resonance (CMR) have demonstrated distinct patterns of cardiovascular remodelling associated with classical vascular risk factors (7). Examining the potential sex differential impact of risk factors on cardiovascular phenotypes may provide insights into differences in cardiovascular disease patterns between men and women. However, this has not been addressed in existing work.

The application of radiomics analysis to CMR images allows extraction of multiple indices of ventricular shape and myocardial texture (8). Previous work has demonstrated the feasibility of CMR radiomics models for discrimination of health from disease (9–12), including distinction of vascular risk factors (13). These studies have focused on development of machine learning models optimised for disease discrimination using CMR radiomics features as input variables. CMR radiomics analysis may also be used for detailed cardiovascular phenotyping, with the potential to provide novel insights into disease processes. However, the approach of existing work does not allow granular evaluation of independent associations of CMR radiomics features with individual risk factors.

In this study, we demonstrate the utility of CMR radiomics analysis as a tool for detailed cardiovascular phenotyping. We characterise independent associations of sex, age, and key vascular risk factors with cardiovascular radiomics phenotypes and explore potential sex and age differential relationships.

## Methods

### Setting and Study Population

The UK Biobank is a very large cohort study comprising detailed characterisation of over 500,000 men and women from rural and urban settings across the UK. Individuals aged 40–69 years-old were identified from National Health Service (NHS) registers and recruited through postal invitation between 2006 and 2010. Individuals who were unable to consent or complete baseline assessment due to illness or discomfort were not included. There was baseline characterisation of demographics, lifestyle, and medical history of participants as well as blood sampling for selected biomarkers. The UK Biobank protocol is detailed in a dedicated document (14). The UK Biobank dataset is linked to routine national data sources including Hospital Episode Statistics (HES) and death registers, permitting continuous longitudinal tracking of incident health outcomes for the whole cohort (15). The UK Biobank imaging study, which includes, amongst other things, detailed CMR scanning, aims to image a random 20% (*n* = 100,000) subset of the original participants. To date (June 2021), ~50,000 participants have completed the UK Biobank imaging study.

### Background to CMR Radiomics

The application of radiomics analysis to CMR images is a novel technique allowing extraction of quantitative measures of ventricular shape and myocardial texture. Image segmentations used for conventional image analysis may be used to define regions of interest for radiomics analysis, which typically include the ventricular cavities and the left ventricular (LV) myocardium. These segmentations are used to build 3D masks of the defined regions of interest, from which radiomics features are extracted. There are three categories of radiomics features: shape, first-order, and texture. The shape features provide advanced geometric quantification of the region of interest, including volume, axial dimensions, and quantitative descriptions of the overall shape (e.g., elongation, sphericity, flatness). The first-order and texture features are derived from analysis of the distribution and pattern of voxel signal intensity levels in the defined region of interest. The signal intensities in magnetic resonance images reflect magnetic properties of the underlying tissue, which are in turn influenced by tissue composition (16). Thus, radiomics signal intensity features applied to the LV myocardium may provide insight into myocardial tissue characteristics. First-order radiomics features describe the global distribution of signal intensities in the region of interest using histogram based statistics such as mean, variation, and skewness. Texture features rely on higher order statistics to describe local signal intensity patterns. Further details on CMR radiomics are provided in a dedicated review paper (8).

### CMR Image Acquisition

The UK Biobank imaging study is performed using uniform pre-defined standard operating procedures, equipment, and staff training (17). CMR imaging was performed with 1.5 Tesla scanners (MAGNETOM Aera, Syngo Platform VD13A, Siemens Healthcare, Erlangen, Germany), the acquisition protocol is published elsewhere (18). Cardiac function assessment comprised three long axis cines (horizontal long axis, vertical long axis, left ventricular outflow tract sagittal and coronal) and a complete short axis stack covering the left and right ventricles acquired at one slice per breath hold using balanced steady-state free precession (bSSFP) sequences. Typical acquisition parameters are as follows: TR/TE = 2.6.1.1 ms, flip angle 80°, Grappa factor 2, voxel size 1.8 mm × 1.8 mm × 8 mm (6 mm for long axis). The actual temporal resolution of 32 ms was interpolated to 50 phases per cardiac cycle (~20 ms) (18). With the exception of distortion correction, no signal or image filtering was applied.

### CMR Image Segmentation

The first 5,000 UK Biobank CMR scans were manually segmented using CVI^42^®post-processing software (Version 5.1.1, Circle Cardiovascular Imaging Inc., Calgary, Canada). The analysis protocol has been previously published (19). In brief, LV endocardial and epicardial borders were contoured in end-diastole and end-systole in the short axis stack images. End-diastole was defined as the first phase of the acquisition. End-systole was selected as the cardiac phase at which the mid-ventricular LV intra-cavity blood pool appeared smallest by visual inspection. The LV papillary muscles were considered part of the blood pool (excluded from LV mass). The right ventricular (RV) endocardial borders were segmented in end-diastole and end-systole. The most basal slice for the LV was included in the segmentation if at least half of the LV blood pool circumference was surrounded by myocardium. The pulmonary valve plane was used to define the most basal RV slice, with volumes below the valve plane considered as part of the RV. This ground truth manual analysis set, was used to develop a fully automated image analysis pipeline with inbuilt quality control (20). Details of reproducibility performance of the automated algorithm are available in dedicated publications (19–21). This pipeline has been propagated to the first 32,068 UK Biobank CMR studies, which, along with their corresponding segmentations, were available for inclusion in the present study.

### Radiomics Feature Extraction

The segmentations from the short axis stack, described above, were used to define three regions of interest for radiomics analysis: RV cavity, LV cavity, LV myocardium. Features are calculated from 3D volumes of these ROIs. To reduce intensity level variations attributable to the acquisition process, we performed intensity normalisation of images through histogram matching, using as reference one of the studies from the dataset (22). For grey level discretisation, we used a fixed bin width of 25 intensity values. We extracted shape features from the RV and LV cavity. From the LV myocardium, we extracted signal intensity-based radiomics features (first order, texture). Radiomics features were extracted using the PyRadiomics open source platform version 2.2.0 (23). Thus, a total of 237 radiomics features were included in the analysis for each CMR study (LV shape *n* = 26, RV shape *n* = 26, LV myocardium first-order *n* = 36, LV myocardium texture *n* = 148). The full list of radiomics features included in the analysis is presented in Supplementary Table 1.

### Feature Clustering

**Figure 1:**
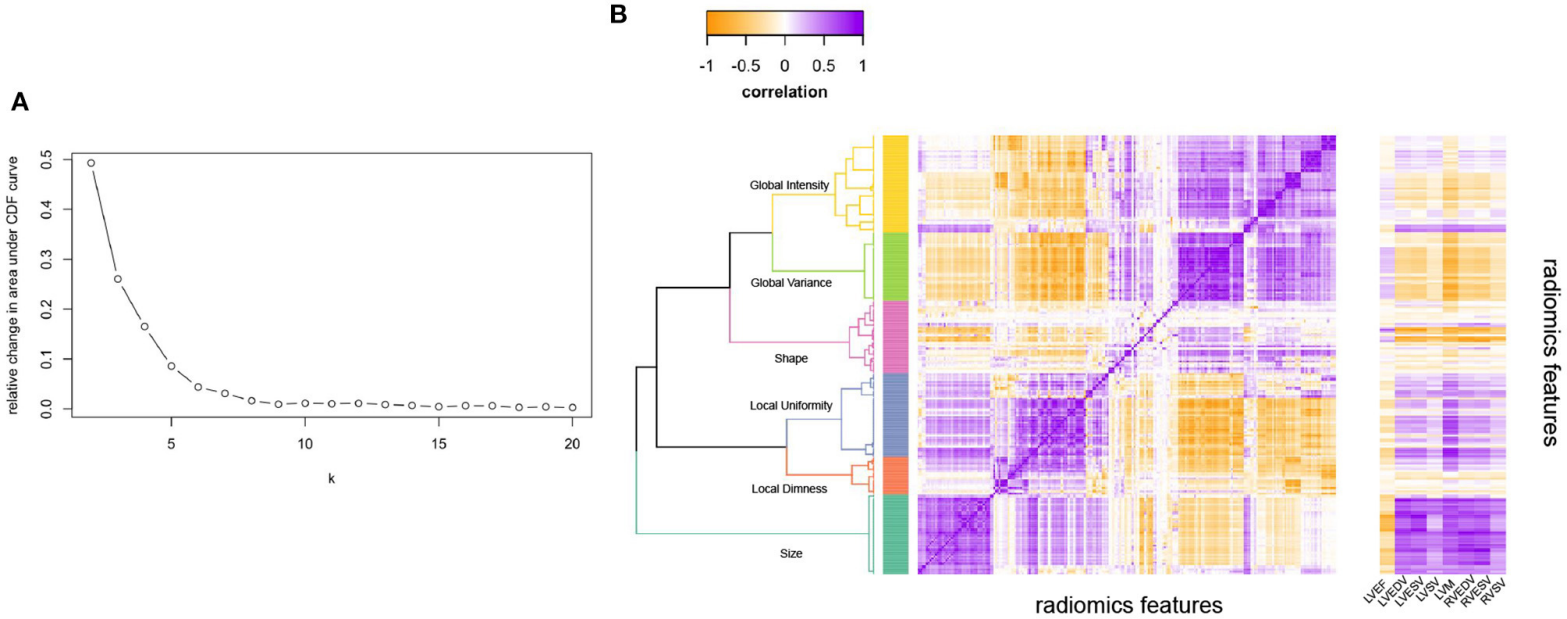
Illustrating the clustering method and approach to defining the number of radiomics feature clusters for the radiomics features. **(A)** Illustrates the relative change in area under the CDF (Consensus Cumulative Distribution Function) curve of the y axis with increasing number of clusters (k on x axis), with the curve levelling off at six clusters. **(B)** Is the correlation heatmap illustrating the six defined clusters, with the darkest purple indicating perfect positive correlation and darkest yellow perfect negative correlation. The dendrogram indicates the six clusters from hierarchical clustering. The ribbon on the right of **(B)** Illustrates correlation of each radiomics feature with the conventional metrics indicated on the x-axis. LVEDV, left ventricular end-diastolic volume; LVEF, left ventricular ejection fraction; LVESV, left ventricular end-systolic volume; LVM, left ventricular mass; RVEDV, right ventricular end-diastolic volume; RVESV, right ventricular end-systolic volume; RVSV, right ventricular stroke volume.

As the number of radiomics was large, to aid interpretation, we grouped inter-correlated radiomics features using hierarchical cluster analysis (Figure 1) (24). More precisely, features were clustered using Ward's algorithm (Ward. D linkage function in R) so that variance is minimised within clusters with distance measured via Pearson coefficient (1-r) (25). The clusters were defined using features derived from participants free from cardiovascular disease and vascular risk factors. The optimal number of clusters was selected via consensus clustering using the ConsensusClusterPlus v1.50 function in R which allows for calculating quantitative stability evidence for determining the number and membership of possible clusters in an unsupervised manner (26). We assessed the curve for the change in the area under the Consensus Cumulative Distribution Function (CDF) and chose the number of clusters at which the area under the CDF no longer appreciably increases (the elbow). At six clusters, the CDF curve levelled off and all but one cluster had high consensus (Table 1; Figure 1), so we chose six clusters. We then assigned descriptive names to each cluster based on the properties of its constituent features, as summarised in Table 1.

**Table 1:** Summary of the six defined radiomics feature clusters including their assigned names, example features, and properties represented by the features within each cluster.

**Cluster name**	**Example features**	**Description of feature properties**	**Consensus D1**
Size	Volume surface area	Size of the ventricles	0.98
Local uniformity	First-order uniformity GLSZM Large area emphasis	Size of areas with the same intensity level within myocardium	0.67
Global variance	First-order variance GLCM contrast	Variance of myocardial intensity level distribution	0.51
Shape	Shape elongation shape sphericity	Descriptors of overall ventricular shape	0.96
Local dimness	GLDM low grey level emphasis GLSZM low grey level zone emphasis	Relative presence of areas of low signal intensity level	0.78
Global intensity	First-order mean first-order energy	Average brightness of myocardial intensity level	0.70

*GLCM, Grey Level Co-occurrence Matrix; GLDM, Grey Level Dependence Matrix; GLSZM, Grey Level Size Zone Matrix. Consensus D1 indicates the repeatability of cluster components on repeated clustering, that is the likelihood that the same features appear in the cluster if the clustering analysis is repeated. Higher values within the shape category indicate greater sphericity and less elongated ventricular shapes. Please note, for computational reasons in Pyradiomics the “flatness” and “elongation” features are reported as inverse values, thus higher elongation and flatness values indicate less elongated more spherical shapes (and vice versa)*.

Additionally, we examined correlation of conventional CMR metrics with all the radiomics features (Figure 1B). Conventional metrics correlated most strongly with radiomics features in the “size” cluster; correlation with other radiomics features was weak and inconsistent. Indicating that although there is some overlap between CMR radiomics features and conventional metrics, there are also many areas where radiomics features provide information that is different and uncorrelated to conventional metrics. Notably, LV mass additionally showed significant correlations with features in the “local variance” and “global uniformity” clusters. This may reflect dependency of these signal intensity-based features on ROI size (LV mass reflects the size of the myocardium ROI from which the texture features are extracted). It is also possible, that these metrics represent myocardial tissue alterations present in individuals with elevated LV mass (e.g., myocardial fibrosis).

### Definition of the Study Sample

We first considered variation in radiomics features by sex and age in a healthy subset of participants. This analysis included participants without cardiovascular disease or vascular risk factors at time of imaging. For analysis of associations with vascular risk factors, we considered individuals who had vascular risk factors, but not cardiovascular disease. To create a balanced analysis sample, individuals with at least one vascular risk factor were matched on age and sex with participants without vascular risk factors (Supplementary Figure 1).

We considered cardiovascular disease as any ischaemic heart disease, non-ischaemic cardiomyopathy, valvular disease, or significant arrhythmia. These were ascertained from a combination of self-reported answers at baseline interview, UK Biobank algorithmically derived outcomes, and linked HES data codes (Supplementary Table 2). The following vascular risk factors were considered: hypertension, diabetes, high cholesterol, and current smoking. These were also defined by reference to a combination of self-reported answers, HES records, and blood biochemistry data (Supplementary Table 3). Age was taken as recorded at the time of imaging. Sex was taken from self-report at baseline.

### Statistical Analysis

Statistical analysis was performed using R version 3.6.222 (27). Within the healthy subset, we estimated the independent associations of sex and age with individual radiomics features using multivariable linear regression models adjusted for body surface area. We calculated standardised beta coefficients, 95% confidence intervals, and *p*-values associated with age and sex for each radiomics feature. For ease of interpretation, we grouped these results within the previously defined feature clusters (Table 1). We calculated the average beta coefficient and confidence intervals for associations in each cluster. The full detail of associations of age and sex with individual radiomics features is presented in Supplementary Table 4.

To examine the association of vascular risk factors with radiomics features, we created a balanced cohort comprising a 1:1 ratio of “risk factor” and “no risk factor” individuals. To accomplish this, we estimated propensity scores from a logistic glm predicting presence of at least one risk factor from age and sex. Subjects with at least one risk factor were paired with their nearest neighbour with no risk factor using the R package matchit 4.1.0 (28). Thus, the analysis sample comprised an equal number of individuals with vascular risk factors and those without vascular risk factors matched on age and sex. Within this sample, we entered all the vascular risk factors in a mutually adjusted multivariable linear regression model to estimate the independent association of each risk factor with individual radiomics features adjusting for age, sex, and body surface area. As before, we organise these results within the previously defined clusters, reporting the average beta coefficient and confidence interval for each cluster. We present the results for associations of each vascular risk factor with individual radiomics features in Supplementary Table 5.

For all associations, we tested for potential differential relationships by sex and age, using interaction terms in fully adjusted models and explored the nature of any significant interactions in stratified analyses. We adjusted for multiple testing using a conservative Bonferroni correction per number of features (p^*^237).

## Results

### Baseline Participant Characteristics

CMR data was available for 32,068 UK Biobank participants, comprising 15,443 (48.2%) men and 16,625 women (51.8%) with average age of 63.3 ± 7.5 years (Table 2). The rates of diabetes, high cholesterol, hypertension, and smoking were 5.9%, 34.8%, and 32.9%, respectively (Table 2). Ischaemic heart disease was the most common cardiovascular disease and was observed in 6.0% of participants (Table 2). Overall, there were 3,528 (11.0%) participants with documented cardiovascular disease (Supplementary Figure 1).

**Table 2:** Baseline participant characteristics.

	**All participants**	**Healthy subset**	**Matched vascular risk factor cohort**
Total population	32,068	14,902	27,400
Men	15,443 (48.2%)	6,095 (40.9%)	13,290 (48.5%)
Women	16,625 (51.8%)	8,807 (59.1%)	14,110 (51.5%)
Age at imaging (years)	63.3 ± 7.5	61.0 ± 7.3	63.4 ± 7.2
Body surface area (m^2^)	1.9 ± 0.2	1.8 ± 0.2	1.9 ± 0.2
Body mass index (Kg/m^2^)	26.6 ± 4.2	25.6 ± 3.8	26.6 ± 4.2
Ischaemic heart disease	1,937 (6.0%)	0	0
Valvular heart disease	582 (1.8%)	0	0
Non-ischaemic cardiomyopathies	59 (0.2%)	0	0
Heart failure unspecified aetiology	191 (0.6%)	0	0
Cardiac arrhythmia	1,443 (4.5%)	0	0
Diabetes	1,881 (5.9%)	0	1,471
High cholesterol	11,161 (34.8%)	0	8,848
Hypertension	10,545 (32.9%)	0	8,322
Smoking (current)	1,157 (3.6%)	0	1,038

*Continuous variables are summarised as mean ± standard deviation and count variables as number of participants (percentage of total)*.

Exclusion of individuals with cardiovascular disease and vascular risk factors, resulted in a sample of 14,902 participants, which were considered as the healthy subset. This cohort comprised 6,095 men and 8,807 women, with mean ages of 61.5 ± 7.6 years and 60.7 ± 7.1 years, respectively (Table 2). The matched cohort comprised 13,700 individuals with at least one vascular risk factor matched 1:1 on age and sex to healthy participants creating a total analysis sample of 27,400 participants (Supplementary Figure 1; Table 2).

### Variation of Radiomics Features by Age and Sex in the Healthy Subset

#### Associations of Sex With Radiomics Features in the Healthy Subset

**Figure 2:**
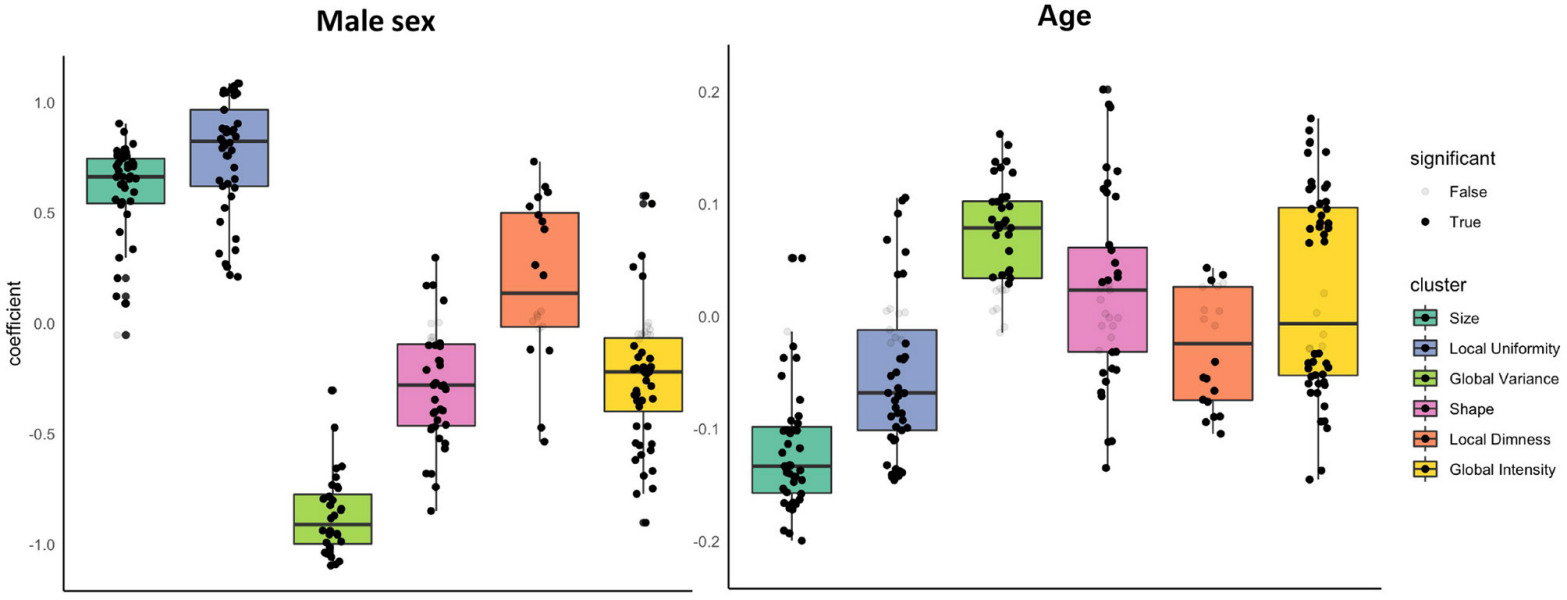
Associations of sex and age with radiomics features in the healthy subset grouped into clusters. Results are from linear regression models adjusted for age, sex, and body surface area. The y axis is standardised beta coefficients for associations of sex (left) and age (right) with radiomics features. Each dot represents point estimate of the association with a radiomic feature from a separate model. Black dots indicate statistically significant associations. Grey dots indicate non-significant associations. Statistical significance is based on Bonferroni adjusted *p*-value < 0.05. Feature associations are grouped into previously defined clusters (Figure 1; Table 1). The dark line in the box plot indicates the median beta coefficient in the cluster, the box borders indicate limits of the interquartile range.

We estimated the association of sex with radiomics features in the healthy subset, whilst adjusting for age and body size. Full details of all linear regression coefficients and *p*-values are presented in Supplementary Table 4. For ease of interpretation, we group associations into previously defined feature clusters and calculate the mean beta coefficient for each cluster (Table 3; Figure 2).

**Table 3:** Relationship of sex and age with radiomics features in the healthy subset expressed as the average association within each of the six radiomics feature clusters.

		**Radiomics feature clusters**						
**Exposures**		**Size**	**Local uniformity**	**Global variance**	**Shape**	**Local dimness**	**Global intensity**	**Totals**
Sex (Male)	Mean beta	0.58	0.76	−0.90	−0.28	0.19	−0.24	
	95% CI	0.51, 0.66	0.68, 0.84	−0.97 to−0.84	−0.36 to −0.19	0.02, 0.36	−0.33 to −0.16	
	Significant features, *n* (%)	41 (95%)	45 (100%)	37 (100%)	34 (87%)	14 (70%)	43 (83%)	214 (91%)
Age	Mean beta	−0.12	−0.05	0.07	0.02	−0.02	0.02	
	95% CI	−0.14 to −0.10	−0.07 to −0.03	0.06, 0.09	−0.00 to 0.05	−0.05 to −0.00	−0.00 to 0.05	
	Significant features, *n* (%)	42 (98%)	37 (82%)	29 (78%)	27 (69%)	13 (65%)	46 (89%)	194 (82%)
Sex*age	Mean beta	−0.01	−0.07	0.03	0.02	0.00	−0.01	
	Lower CI	−0.015 to −0.00	−0.08 to −0.06	0.01, 0.04	0.00, 0.03	−0.02 to 0.03	−0.02 to 0.00	
	Significant features, *n* (%)	3 (7%)	22 (49%)	11 (30%)	7 (18%)	4 (20%)	8 (15%)	55 (23%)
	Total features in cluster (*n*)	43	45	37	39	20	52	236

Results are the mean beta coefficient and 95% CI for associations of each exposure with the features within each cluster. Beta indicates standard deviation change in radiomics feature per 1 unit/standard deviation change in the exposure. Models are mutually adjusted for age and sex, and include additional adjustment for body surface area. The interaction term is from a separate fully adjusted model. For sex, the reference level is set as “female.” “Significant features” indicates the number and percentage of features with a statistically significantly association within each cluster, based on a Bonferroni adjusted p-value. CI, confidence interval. *indicated multiplication for the interaction terms.

There were significant associations between sex and radiomics features across all feature clusters. Compared to women, men had larger ventricular cavity sizes (“size” cluster, average beta: 0.58, 95% CI: 0.51, 0.66), with a less spherical overall shape of the ventricles (“shape” cluster, mean beta: −0.28, 95% CI: −0.36 to −0.19), these shape alterations were broadly consistent for the LV and RV (Supplementary Table 4). There were also distinct differences in the distribution and patterns of signal intensities of the LV myocardium for men and women. Men had, on average, lower global signal intensity values (“global intensity” cluster, mean beta: −0.24, 95% CI: −0.33 to −0.16) and less variation in intensity values (“global variance” cluster, average beta: −0.90, 95% CI: −0.97 to −0.84). Furthermore, men showed enhanced measures of local dimness patterns (“local dimness” cluster, mean beta: 0.19, 95% CI: 0.02, 0.36) indicating greater relative presence of areas of low signal intensity in the LV myocardium compared to women. Consistent with this observation, men also had greater local uniformity of myocardial signal intensities (“local Uniformity” cluster, mean beta: 0.76, 95% CI: 0.68, 0.84), indicating a more homogeneous appearance of myocardial signal intensity levels. Thus, overall, compared to women men had larger more elongated ventricles with dimmer and less texturally complex appearance of the LV myocardium intensities.

#### Associations of Age With Radiomics Features in the Healthy Subset

We next considered, the association of age with each radiomics feature whilst adjusting for sex and body size. We report all linear modelling results in Supplementary Table 4. For ease of interpretation, we group associations into previously defined feature clusters and calculate the mean beta coefficient for each cluster (Table 3; Figure 2). Compared to associations between sex and radiomics features, there were fewer statistically significant associations with age and, in general, the magnitudes of effects were smaller.

As expected, older age was associated with smaller ventricular cavity size (“size” cluster, average beta: −0.12, 95% CI: −0.14 to −0.10). The were no significant alterations of the overall ventricular shape with ageing based on the mean associations within the shape cluster (beta: 0.02, 95% CI: −0.00 to 0.05). Examination of individual feature associations revealed association of increasing age with less spherical LV and more spherical RV shape (Supplementary Table 4).

Older age was associated with greater variation in myocardial intensity levels (“global variance” cluster, mean beta: 0.07, 95% CI: 0.06, 0.09), but without significant alteration in the average myocardial brightness (“global intensity” cluster, mean beta: 0.02 95% CI: −0.00 to 0.05). Corresponding to the increased variance, average local uniformity in textures decreased with increasing age (“local uniformity” cluster, mean beta: −0.05, 95% CI: −0.07 to −0.03) and there was decrease in local dimness patterns (“local dimness” cluster, average beta: −0.02, 95% CI: −0.05 to −0.00). Overall, myocardial signal intensity alterations with age appear mixed with a broad pattern indicating dimmer hearts in end systole and brighter hearts in end diastole.

#### Sex Differential Age-Related Alterations in Radiomics Features

We tested for potential sex differential age related alterations of radiomics features through consideration of interaction terms (sex^*^age) in models additionally adjusted for age, sex, and body size (Supplementary Table 4; Supplementary Figure 2; Table 3). Overall, ageing related changes in radiomics features appeared consistent for men and women. Relatively few features show a significant sex-age interaction (*n* = 55, 23%) and most clusters had a mean interaction effect close to zero (Supplementary Table 4; Supplementary Figure 2).

**Figure 3:**
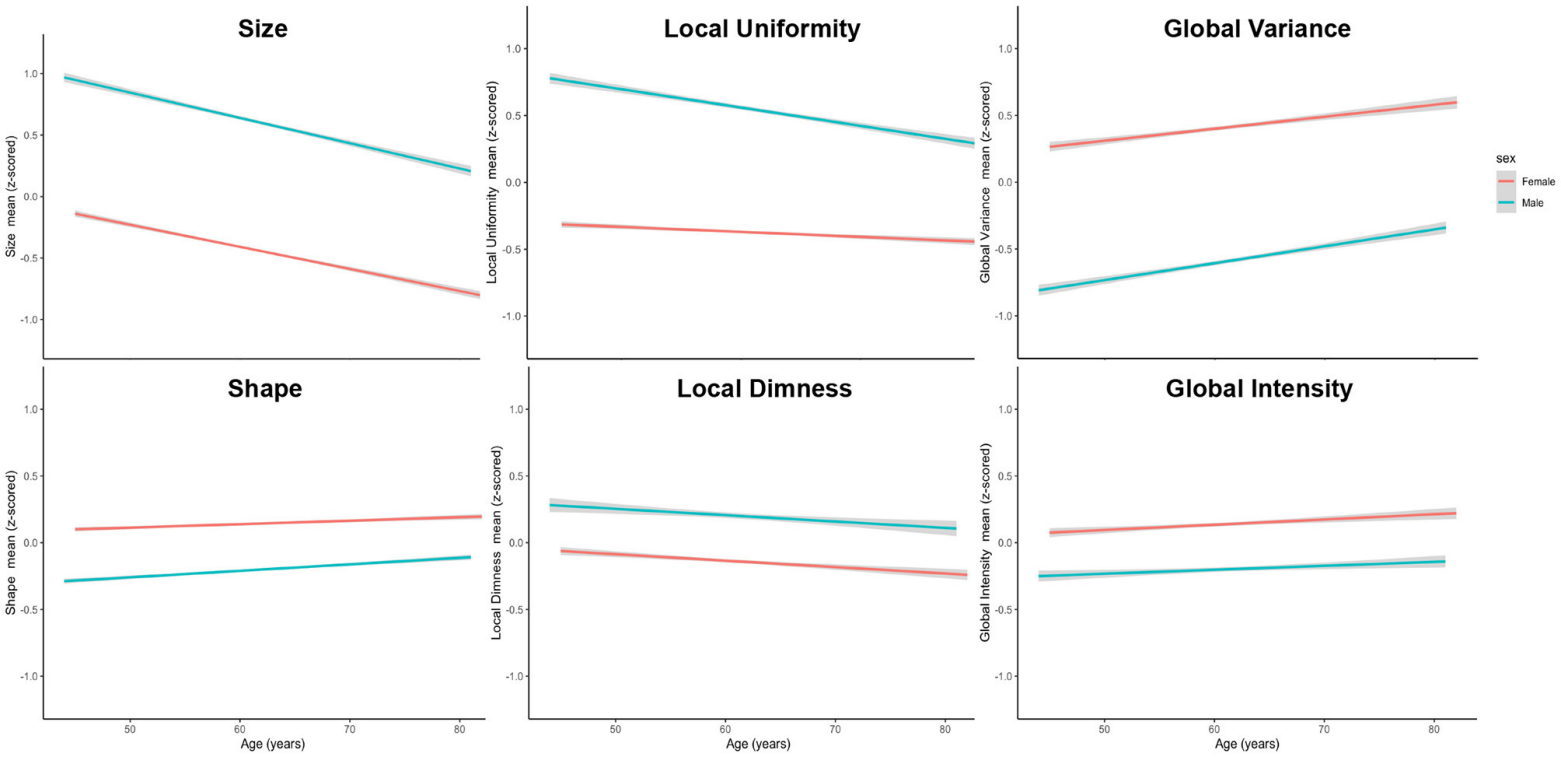
Mean standardised radiomics value for each feature cluster stratified by sex across all ages. Men had larger (higher size values) and more elongated (higher shape values) ventricles than women. Men had dimmer less varied signal intensities at both a global (lower global intensity, lower global variance) and local (higher local uniformity, higher local dimness) level. Alteration of radiomics features with ageing were generally consistent for men and women. There was more rapid decline in local uniformity in men with minimal age-related change in this cluster for women.

To further visualise variation of radiomics features with age in men and women, we plotted the mean z-scored radiomics value within each cluster stratified by sex across all ages (Figure 3). Overall, age-related changes in radiomics feature clusters were, on average, consistent for men and women. The local uniformity cluster had the largest number of features with statistically significant age-sex interactions (*n* = 22). On average, men had higher local uniformity, which declined with age. Women had lower local uniformity compared to men with little change in the features within this cluster with ageing.

### Variation of Radiomics Features With Vascular Risk Factors

**Figure 4:**
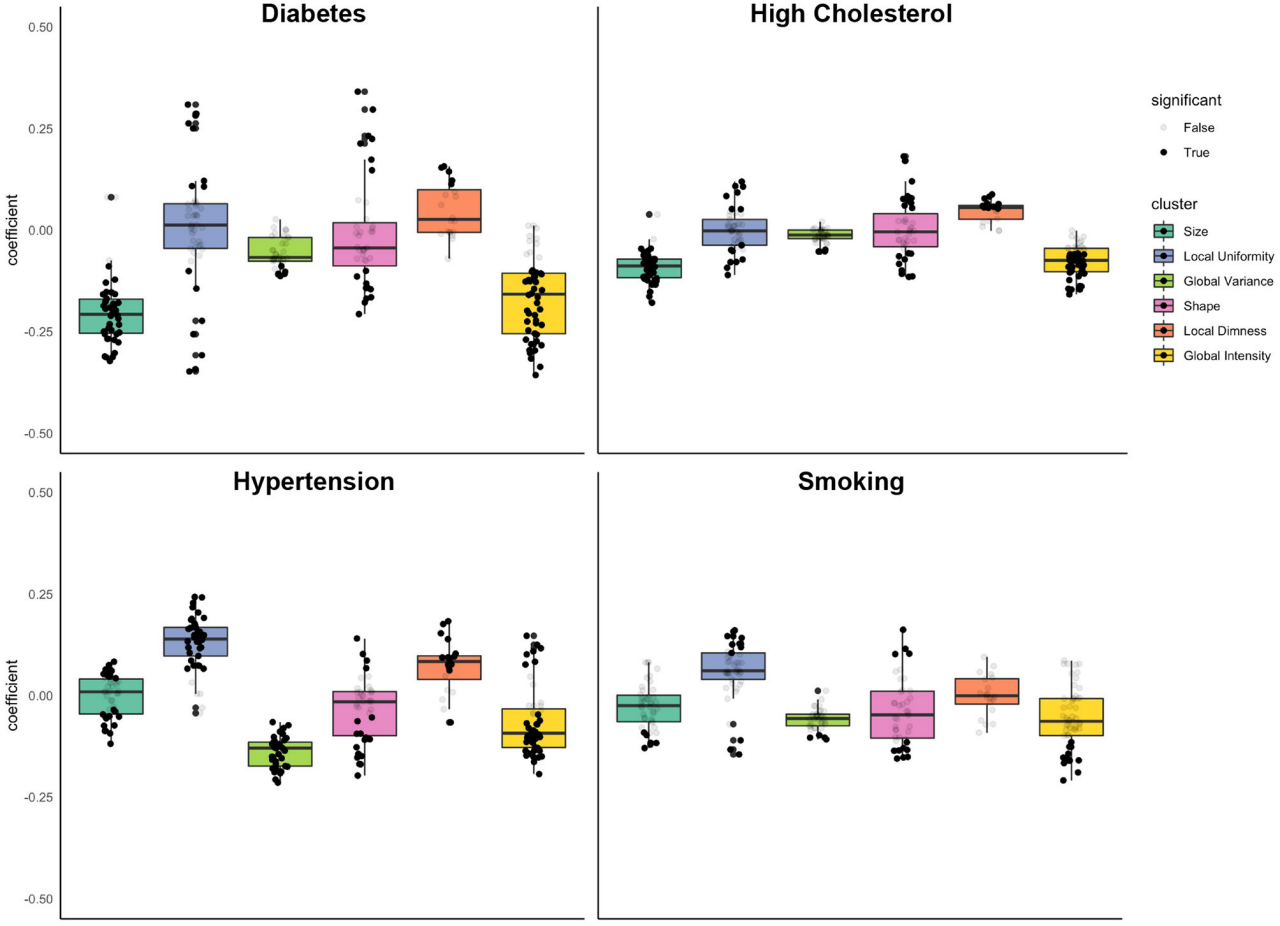
Associations of diabetes, high cholesterol, hypertension, and smoking with radiomics features grouped into clusters. Results are from linear regression models adjusted for age, sex, and body surface area, diabetes, high cholesterol, hypertension, and smoking. The y axis is standardised beta coefficients for associations of vascular risk factors (diabetes, high cholesterol, hypertension, smoking) with radiomics features. Each dot represents point estimate of association with a radiomic feature from a separate model. Black dots indicate statistically significant associations. Grey dots indicate non-significant associations. Statistical significance is based on Bonferroni adjusted *p*-value < 0.05. Feature associations are grouped into previously defined clusters (Figure 1; Table 1). The dark line in the box plot indicates the median beta coefficient in the cluster, the box borders indicate limits of the interquartile range.

In the matched cohort (*n* = 27,400), we estimated the independent association of vascular risk factors with radiomics features in multivariable linear regression models mutually adjusted for all the risk factors and additionally adjusting for age, sex, and body surface area. Modelling results for the associations of the vascular risk factors with each radiomics feature are reported in Supplementary Table 5. For ease of interpretation, we group associations into previously defined feature clusters and calculate the mean beta coefficient for each cluster (Table 4; Figure 4). We discuss associations with each vascular risk factor in turn.

**Table 4:** Relationship of vascular risk factors with radiomics features in the healthy subset expressed as the average association within each of the six radiomics feature clusters.

		**Radiomics feature clusters**						
**Exposure**		**Size**	**Local uniformity**	**Global variance**	**Shape**	**Local dimness**	**Global intensity**	**Totals**
Diabetes	Mean beta	−0.20	0.006	−0.06	−0.01	0.05	−0.17	
	95% CI	−0.23 to −0.17	−0.039 to 0.05	−0.07 to −0.04	−0.05 to 0.04	0.02, 0.08	−0.20 to −0.14	
	Significant features, *n* (%)	40 (93%)	15 (33%)	6 (16%)	17 (44%)	5 (25%)	42 (81%)	125 (53%)
Diabetes*sex	Mean	−0.13	−0.050	0.094	0.028	−0.028	0.019	
	95% CI	−0.15 to −0.11	−0.08 to −0.02	0.08, 0.11	−0.01 to 0.06	−0.05 to −0.01	−0.00 to 0.04	
	Significant features, *n* (%)	14 (33%)	4 (9%)	0 (0%)	3 (8%)	0 (0%)	2 (4%)	23 (10%)
Diabetes*age	Mean	0.01	−0.00	0.01	−0.00	0.01	0.00	
	95% CI	0.00, 0.01	−0.01 to 0.00	0.00, 0.01	−0.01 to 0.01	0.00, 0.01	−0.01 to 0.01	
	Significant features, *n* (%)	0 (0%)	0 (0%)	0 (0%)	0 (0%)	0 (0%)	0 (0%)	0 (0%)
High cholesterol	Mean	−0.09	−0.00	−0.01	0.00	0.05	−0.08	
	95% CI	−0.10 to 0.08	−0.02 to 0.02	−0.02 to −0.01	−0.02 to 0.02	0.04, 0.06	−0.09 to 0.07	
	Significant features, *n* (%)	40 (93%)	15 (33%)	3 (8%)	19 (49%)	12 (60%)	37 (71%)	126 (53%)
High cholesterol*sex	Mean	−0.04	−0.06	0.08	0.02	−0.01	0.01	
	95% CI	−0.05 to −0.02	−0.08 to −0.05	0.07, 0.09	0.00, 0.04	−0.04 to 0.01	−0.00 to 0.02	
	Significant features, *n* (%)	10 (23%)	21 (47%)	18 (49%)	3 (8%)	0 (0%)	4 (8%)	56 (24%)
High cholesterol*age	Mean	0.03	−0.00	0.01	0.01	−0.01	0.02	
	95% CI	0.02, 0.03	−0.01 to 0.00	0.01, 0.02	0.00, 0.02	−0.01 to −0.01	0.02, 0.03	
	Significant features, *n* (%)	7 (16%)	0 (0%)	0 (0%)	0 (0%)	0 (0%)	0 (0%)	7 (3%)
Hypertension	Mean	−0.00	0.13	−0.14	−0.04	0.07	−0.07	
	95% CI	−0.02 to 0.01	0.11, 0.15	−0.15 to −0.13	−0.06 to −0.01	0.04, 0.10	−0.09 to −0.04	
	Significant features, *n* (%)	23 (54%)	40 (89%)	37 (100%)	18 (46%)	15 (75%)	43 (83%)	176 (75%)
Hypertension*sex	Mean	−0.03	−0.03	0.11	0.03	0.04	0.02	
	95% CI	−0.05 to −0.02	−0.05 to −0.01	0.10, 0.13	0.01, 0.05	0.02, 0.07	0.01, 0.03	
	Significant features, *n* (%)	5 (12%)	9 (20%)	25 (68%)	7 (18%)	2 (10%)	5 (10%)	53 (23%)
Hypertension*age	Mean	0.02	−0.03	0.03	0.01	−0.01	0.02	
	95% CI	0.01, 0.02	−0.03 to −0.02	0.03, 0.04	−0.00 to 0.01	−0.02 to −0.01	0.01, 0.03	
	Significant features, *n* (%)	1 (2%)	2 (4%)	7 (19%)	0 (0%)	0 (0%)	6 (12%)	16 (7%)
Smoking	Mean	−0.03	0.06	−0.06	−0.04	0.00	−0.06	
	95% CI	−0.05 to −0.01	0.04, 0.08	−0.07 to −0.05	−0.07 to −0.01	−0.02 to −0.03	−0.08 to −0.03	
	Significant features, *n* (%)	6 (14%)	14 (31%)	4 (11%)	12 (31%)	0 (0%)	12 (23%)	48 (20%)
Smoking*sex	Mean	−0.03	−0.01	0.05	0.05	0.05	−0.02	
	95% CI	−0.04 to −0.01	−0.03 to 0.00	0.04, 0.06	0.03, 0.07	0.02, 0.08	−0.04 to 0.00	
	Significant features, *n* (%)	0 (0%)	0 (0%)	0 (0%)	0 (0%)	0 (0%)	0 (0%)	0 (0%)
Smoking*age	Mean	−0.02	−0.00	−0.01	−0.01	0.04	−0.01	
	95% CI	−0.03 to −0.01	−0.01 to 0.01	−0.01 to 0.00	−0.02 to −0.00	0.04, 0.05	−0.02 to −0.01	
	Significant features, *n* (%)	0 (0%)	0 (0%)	0 (0%)	0 (0%)	0 (0%)	0 (0%)	0 (0%)
	Total	43	45	37	39	20	52	236

Results are the mean beta coefficient and 95% CI for associations of each exposure with the features within each cluster. Beta indicates standard deviation change in radiomics feature per 1 unit/standard deviation change in the exposure. Models are mutually adjusted for all the risk factors (diabetes, high cholesterol, hypertension, smoking) and include adjustment for age, sex, and body surface area. Interaction terms are from separate fully adjusted models, separately for age and sex. “Significant features” indicates the number and percentage of features with a statistically significantly association within each cluster, based on a Bonferroni adjusted p-value. CI, confidence interval. *indicated multiplication for the interaction terms.

#### Associations of Diabetes With Radiomics Features

The most prominent diabetes related alterations of radiomics features were within the size and global intensity clusters, with statistically significant associations in 93% (*n* = 40) and 81% (*n* = 42) of features within these clusters, respectively. Diabetes was associated with decreased size of the LV and RV cavities (“size” cluster, mean beta: −0.20, 95% CI: −0.23 to −0.17), decreased global intensity (“global intensity” cluster, mean beta: −0.17, 95% CI: −0.20 to −0.14), lower global variance (“global variance” cluster, mean beta: −0.06, 95% CI: −0.07 to −0.04), and greater local dimness (“local dimness” cluster, mean beta: 0.05, 95% CI: 0.02, 0.08).

Associations at the mean were not significant for the local uniformity and shape clusters. However, considering the clusters more closely (Figure 4), we see that diabetes drives a differential response with both local uniformity and shape clusters. Since there is some within cluster heterogeneity in what features quantify, we examined the coefficient of individual features within each cluster (Supplementary Table 5). For example, within the shape cluster, a number of features quantify intensity variance, and these features trend downward (Supplementary Table 5). This corresponds well-with the observed small but significant trend in global variance. Examination of individual feature associations reveals less spherical LV in end-diastole and more elongated RV in both end-diastole and end-systole (Supplementary Table 5). Overall, diabetes was associated with decreased ventricular size, decreased myocardial intensity (brightness), decreased global variance (variation in intensity levels), and increased local uniformity.

#### Sex and Age Differential Associations of Diabetes With Radiomics Features

To examine the potential sex and age differential association of diabetes with radiomics features, we first considered the separately computed interaction terms (Supplementary Table 6; Supplementary Figure 3). There was no evidence of an age differential relationship, with no significant interaction terms detected for any radiomics feature. For the most part, associations were also consistent for men and women, with a statistically significant interaction term observed in only 10% of radiomics features, the majority of these were from the size cluster (Table 4).

To inspect further, we separated the beta boxplots by sex and compared the distributions of diabetes associations for each cluster (Supplementary Figure 4). We found that no feature showed a difference in direction of average association. For size specifically, women showed a lower average effect size than for men.

#### Associations of High Cholesterol With Radiomics Features

High cholesterol had a unique signature of radiomic changes (Table 4; Figure 4). Like diabetes, high cholesterol was associated with smaller ventricular size (“size” cluster, mean beta: −0.09, 95% CI: −0.10 to 0.08), however the magnitude of this association was smaller than that for diabetes and was not statistically significant. Examination of individual features within the “shape” cluster (specifically: sphericity, elongation, flatness), revealed differential shape associations in the LV and RV, with less sphericity of the former and greater sphericity of the latter (Supplementary Table 5). High cholesterol was also associated with decreased global intensity and slightly increased local dimness. Like diabetes, high cholesterol drives differential changes within the local uniformity cluster. Broadly, high cholesterol was associated with smaller ventricles, dimmer myocardium, and lower variance in myocardial intensities.

#### Sex and Age Differential Associations of High Cholesterol With Radiomics Features

We considered the impact of sex and age on the high cholesterol radiomics associations (Supplementary Table 6; Supplementary Figure 3). We identified few significant interaction effects for sex and age, 24 and 3%, respectively (Table 4). The majority of the significant sex interactions were with features within the local uniformity (*n* = 21) and global variance (*n* = 18) clusters (Table 4). We therefore explored sex differential relationships within these clusters (Supplementary Figure 4). For both clusters, the direction of associations was consistent for men and women, however the degree of the association can differ between the sexes (Supplementary Figure 4). As with diabetes, women showed a slightly lower size decrease with high cholesterol compared to men.

#### Associations of Hypertension With Radiomics Features

Like diabetes and high cholesterol, hypertension was associated with significant decreases in global intensity of the LV myocardium (“global intensity” cluster, average beta: −0.07 95% CI: −0.09 to −0.04). Hypertension was also associated with decreased variation in intensity levels (“global variance,” mean beta: −0.14, 95% CI: −0.15 to −0.13), increased local dimness (“local dimness, average beta: 0.07, 95% CI: 0.04, 0.10), and greater uniformity of local intensity levels (“local uniformity” cluster, average beta: 0.13, 95% CI: 0.11, 0.15). These myocardial alterations were the most consistent relationships observed with hypertension (Table 4; Figure 4).

For both the shape and size feature clusters, the significant associations appeared at the extremes of the beta coefficient distributions within each cluster, rather than at the mean (Figure 4). With regards the shape feature cluster, hypertension was associated with more elongated, less spherical ventricular shapes based on the average cluster association (“shape” cluster, average beta: −0.04, 95% CI: −0.06 to −0.01). Examining individual feature associations, these associations appeared significant for the LV, but not the RV (Supplementary Table 5). The average beta coefficient in the size cluster demonstrated no significant association with hypertension. However, there were significant associations with a number of features (*n* = 23) within this cluster, which lie distal either side of the distribution (Table 4; Figure 4).

#### Sex and Age Differential Associations of Hypertension With Radiomics Features

We examined potential variation of the associations of hypertension with radiomics features by sex and age (Supplementary Table 6; Supplementary Figure 3). The associations with hypertension were largely consistent across age and for men and women. There were significant interaction terms for sex and age in 23 and 7% of features, respectively. Most of the features with significant sex interaction terms belonged to the global variance cluster (Table 4; Figure 4). In stratified analysis, we demonstrate that for both men and women, hypertension is associated with lower global variance; however, women show a greater decrease in global variance than men (Supplementary Figure 4).

#### Associations of Smoking With Radiomics Features

Unlike the three previously considered vascular risk factors, smoking showed little consistent effect on any of the clusters of radiomics features (Table 4; Figure 4). The mean effect within each cluster is near zero (Figure 4). However, individual features show definite dependence on smoking (Supplementary Table 5). For example, end systolic global intensity features (e.g., mean and median signal intensities) all decreased with smoking. Furthermore, there were significant associations with RV shape features, demonstrating association of smoking with less spherical, flatter, and more elongated RV in both end-diastole and end-systole. These shape associations were not statistically significant with the LV (Supplementary Table 5).

In general, signal intensity based associations with smoking trended in similar directions to the other vascular risk factors. Broadly, the myocardium of smokers tends to decrease in global intensity and increase in local uniformity. However, these relationships were not as prominent as those for the other risk factors.

#### Sex and Age Differential Associations of Smoking With Radiomics Features

We found no evidence of differential associations of smoking with radiomics features by sex or age (Table 4; Supplementary Figure 3).

## Discussion

### Summary of Findings

In this large study of UK Biobank participants free from cardiovascular disease, we report novel independent associations of CMR radiomics features with sex, age, diabetes, high cholesterol, hypertension, and smoking.

Amongst healthy participants, whilst adjusting for sex and body size, men had larger more elongated ventricles with dimmer, more homogenous, and less texturally complex appearance of the myocardium compared to women. In healthy ageing, we observed smaller ventricular sizes and greater variation in myocardial signal intensity levels with increasing age, independent of sex and body size.

The pattern of associations with myocardial signal intensity features were broadly similar across vascular risk factors; all were associated with dimmer less varied myocardial signal intensities, greater uniformity of local intensity levels, and greater relative presence of low signal intensity areas. These independent associations with signal intensity phenotypes appeared most prominent with first hypertension and second diabetes. Both diabetes and high cholesterol were associated with smaller ventricular sizes, which appeared of greater magnitude for diabetes. Hypertension was associated with an overall less spherical, more elongated LV shape. Associations with smoking were of smaller magnitude than with other risk factors. Broadly, smoking was associated with significant alteration of RV, but not LV shape features.

In general, these relationships appeared consistent for men and women and across ages. Trends with healthy ageing appeared consistent for men and women, and sex interactions, generally, indicated greater rapidity of age-related phenotypic alterations in men. The associations of diabetes with smaller ventricular size were a prominent feature for diabetic men, but not for women, in whom myocardial intensity features dominated. The association of hypertension with myocardial signal intensity phenotypes also varied by sex with hypertensive women showing a greater decrease in global variance than men.

### Comparison With Existing Work

Our findings of larger ventricular sizes in healthy men compared to women (after adjustment for body size) and reduced ventricular size in healthy ageing are consistent with previous studies using conventional CMR measures (29, 30). Our additional observations relating to greater elongation of male hearts as well as myocardial signal intensity variations have not been previously described. Notably the differences in signal intensity patterns of male hearts resemble alterations we observed in association with vascular risk factors. That is, both male sex and vascular risk factors were associated with dimmer myocardial signal intensities, less variation in intensity patterns, and a more homogeneous appearance of the myocardium. This indicates that, in general, adverse cardiovascular exposures have some common manifestations in radiomics myocardial signal intensity features, perhaps indicating a shared pathophysiological process. Indeed, in a previous study of the associations between meat intake and cardiovascular phenotypes, we observed association of greater red and processed meat intake (adverse exposures) with dimmer and less varied myocardial signal intensities (31). The observation of these same phenotypes in healthy men suggests either undiagnosed vascular risk factors in men, or generally a poorer exposure profile in men than women with regards non-classical risk factors.

The cardiovascular phenotyping of vascular risk factors using conventional analysis of non-invasive imaging has been widely described. Our findings of smaller ventricular sizes associated with diabetes and high cholesterol are consistent with previous studies of the UK Biobank and the Multi-ethnic Study of Atherosclerosis (MESA) cohorts, using conventional CMR analysis (7, 32). In addition, we demonstrate association of male sex and hypertension with alteration of the overall ventricular geometry toward a more elongated shape.

Myocardial intensity alterations were a prominent phenotype of diabetes and hypertension in our study, indicating that myocardial level alterations are key features of these conditions. Previous studies using echocardiography have demonstrated alteration of myocardial acoustic properties, an indicator of myocardial fibrosis, in diabetes and the correlation of this feature with diabetic disease severity and associated complications (33, 34). Similarly, CMR studies using global contrast enhanced myocardial T1 mapping methods, have demonstrated that greater myocardial fibrosis (shorter T1 on contrast enhanced T1 mapping) in patients with diabetes is associated with poorer global longitudinal strain and diastolic dysfunction (35). There are also multiple reports of myocardial scarring and diffuse fibrosis associated with hypertension detectable using contrast and non-parametric mapping CMR techniques (36–39). Thus, it appears likely that myocardial fibrosis is a key component of the pathophysiology of both diabetic and hypertensive cardiomyopathies and that this may be detected using non-invasive imaging. The myocardial intensity alterations in our results also extended to high cholesterol, male sex, and (to a lesser extent) smoking. In a large study of the MESA cohort, Turkbey et al. (37) report associations of male sex, hypertension, and smoking with myocardial fibrosis detected by late gadolinium enhancement CMR images. The myocardial signal intensities in magnetic resonance imaging reflect the magnetic properties of underlying tissue, which in turn are determined by tissue characteristics (16). Thus, it is likely that our observations of signal intensity alterations reflect myocardial tissue characteristics, considered in the context of previous work, these may indicate diffuse myocardial fibrosis as a common pathophysiological process for the conditions considered.

Overall, the patterns of associations were consistent for men and women. There was evidence of potential sex differential alterations for selected features in diabetes and hypertension. In general, myocardial intensity alterations appeared a more important manifestation of these conditions in women than men, possibly indicate greater myocardial fibrosis in women. This observation is consistent with clinical observations of greater propensity for heart failure and specifically heart failure preserved ejection fraction syndromes in women, particularly in the context of diabetes and hypertension (40–43).

In summary, our findings with CMR radiomics analysis support previous reports using echocardiography and conventional CMR and provide more granular quantification of myocardial alterations and novel shape features associated with classical vascular risk factors in a low-risk group without clinically manifest cardiovascular disease.

### Technical Considerations

We adopted several technical approaches for increasing the clarity and statistical power of our results, but these approaches come with assumptions and limitations. First, to derive interpretable groups of related radiomics features, we clustered the features by their correlation in the healthy cohort. In doing this, we assumed that the healthy human population provided the best baseline to define the relationship between radiomics features. However, this approach skewed our identified clusters to group features that naturally correlate in human populations rather than features that correlate *definitionally*. For example, myocardial intensity variance in end systole is in the Global Intensity cluster while myocardial intensity variance in end diastole is in the Global Variance cluster. If we had derived our clusters from digital phantoms instead (44), these two measures of intensity variance would have clustered together. We ultimately argue that clustering by human data works well for interpretability but encourage future studies to consider clustering on phantoms for better “ground truth” associations, although this may not always be feasible.

Another assumption of our work is that controlling for a linear association with BSA is sufficient to control for the relationship between radiomics features and body size. The confounding association between radiomics features and ROI size is well-known (45, 46), and we accounted for this by adjusting our linear regression for participants' BSA. However, it is also likely that radiomics features have complex non-linear relationships with BSA. Therefore, a set of adjustments with non-linear BSA terms in our linear modelling could produce better controls for BSA. However, an optimal approach to body size adjustment of radiomics features is yet to be established and adjustment for BSA in the context of the present study was deemed adequate.

### Strengths and Limitations

The large well-characterised cohort in this study permitted reliable ascertainment of diseases and risk factors of interest. CMR image acquisition and segmentation was performed uniformly for the whole dataset minimising related technical variations. We demonstrate the feasibility of CMR radiomics and its application as a tool for deep cardiovascular phenotyping. Whilst previous studies do not consider confounding, we present associations adjusted for all vascular risk factors, body size, age, and sex. However, there may be other important confounds not considered here. This may be particularly relevant in understanding sex differences in associations, as we know that men and women differ in many other important ways not considered in our models. Associations of non-classical risk factors with radiomics phenotypes and their potential modifying effects on the relationships described in the present study is warranted. For instance, exploration of the influence of environmental, socio-demographic, and early life exposures on cardiac phenotypes may provide novel insights into the impact of these factors on cardiovascular health. The UK Biobank comprised a narrow age range, which may have limited our ability to detect age related alterations in CMR metrics. Exploration of age-related radiomics changes in a cohort with broader spectrum of ages is warranted. Furthermore, validation of our findings in different cohorts and within multi-centre settings is indicated in future work. A key avenue for future research is examining the correlation and incremental clinical value of CMR radiomics, particularly the signal intensity based features, against conventional measures of myocardial tissue character (e.g., native T1, late gadolinium enhancement). Due to the observational nature of the study, we cannot exclude residual confounding or infer causation (in either direction) from our results. Finally, there is need for dedicated studies to understand the biological and clinical significance of these radiomics phenotypes. Understanding the nature of these disease associations can be helpful for future studies with non-classical exposures, where the importance to cardiovascular health may not be so well-understood. Additionally, investigating the incremental utility of radiomics analysis to predict incident health outcomes is a key research question in development of the technique as a novel imaging biomarker.

## Conclusions

In this study we characterise novel associations of sex, age, and major vascular risk factors with cardiovascular radiomics phenotypes. These observations provide new insights into the impact of these risk factors on cardiovascular health, including potential sex differential patterns of remodelling. Further studies into the nature and clinical significance of the defined phenotypes are needed.

## Data Availability Statement

This research was conducted using the UKB resource under access application 2964. UK Biobank will make the data available to all bona fide researchers for all types of health-related research that is in the public interest, without preferential or exclusive access for any persons. All researchers will be subject to the same application process and approval criteria as specified by UK Biobank. For more details on the access procedure, see the UK Biobank website: http://www.ukbiobank.ac.uk/register-apply/. Requests to access the datasets should be directed to http://www.ukbiobank.ac.uk/register-apply/.

## Ethics Statement

The studies involving human participants were reviewed and approved by this study complies with the Declaration of Helsinki; the work was covered by the Ethical approval for UK Biobank studies from the NHS National Research Ethics Service on 17th June 2011 (Ref 11/NW/0382) and extended on 18 June 2021 (Ref 21/NW/0157) with written informed consent obtained from all participants.

## Author Contributions

SP, KL, NH, and ZR-E conceived the idea. AJ led and conducted the analysis. PG extracted and prepared the radiomics features. CM contributed to data preparation. ZR-E and AJ wrote the manuscript. All authors have read, provided critical feedback, and approved the final version of the manuscript.

## Funding

This project was enabled through access to the MRC eMedLab Medical Bioinformatics infrastructure, supported by the Medical Research Council (www.mrc.ac.uk; MR/L016311/1). PM and SP acknowledge support from the National Institute for Health Research (NIHR) Barts Biomedical Research Centre. SP acknowledges support from the SmartHeart EPSRC programme grant (www.nihr.ac.uk; EP/P001009/1) and from the CAP-AI programme, Londons first AI enabling programme focused on stimulating growth in the capital's AI Sector. CAP-AI is led by Capital Enterprise in partnership with Barts Health NHS Trust and Digital Catapult and was funded by the European Regional Development Fund and Barts Charity. SP acts as a paid consultant to and is a shareholder of Circle Cardiovascular Imaging Inc., Calgary, Canada and Servier. SP acknowledges the British Heart Foundation for funding the manual analysis to create a cardiovascular magnetic resonance imaging reference standard for the UK Biobank imaging resource in 5000 CMR scans (www.bhf.org.uk; PG/14/89/31194). PG, KL, and SP have received funding from the European Union's 2020 research and innovation programme under grant agreement No 825903 (euCanSHare project). KL received funding from the Spanish Ministry of Science, Innovation and Universities under grant agreement RTI2018-099898-B-I00. NH acknowledges support from UK Medical Research Council (MC_UU_12011/1) and NIHR Southampton Biomedical Research Centre, University of Southampton and University Hospital Southampton. NA recognise the National Institute for Health Research (NIHR) Integrated Academic Training programme which supports his Academic Clinical Lectureship posts. ZR-E was supported by British Heart Foundation Clinical Research Training Fellowship No. FS/17/81/33318. AJ was supported by a Fulbright Predoctoral Research Award (2019-2020).

## Conflict of Interest

SP provides consultancy to and owns stock of Cardiovascular Imaging Inc, Calgary, Alberta, Canada. The remaining authors declare that the research was conducted in the absence of any commercial or financial relationships that could be construed as a potential conflict of interest. The handling editor declared a past co-authorship with one of the authors SP.

## Publisher's Note

All claims expressed in this article are solely those of the authors and do not necessarily represent those of their affiliated organizations, or those of the publisher, the editors and the reviewers. Any product that may be evaluated in this article, or claim that may be made by its manufacturer, is not guaranteed or endorsed by the publisher.
