# Supplementary material for: Cardiac Magnetic Resonance Radiomics Reveal Differential Impact of Sex, Age, and Vascular Risk Factors on Cardiac Structure and Myocardial Tissue
Source: Front Cardiovasc Med. 2021 Dec 22;8:763361. doi: 10.3389/fcvm.2021.763361 (PMC8727756; doi:10.3389/fcvm.2021.763361)
Supplement: Supplementary file 1 [file Data_Sheet_1.docx]

**Supplementary Table 1. Radiomics features included in this study grouped by feature category and cluster grouping**

| Type | Subtype | ROI | Phase | Feature | Cluster |
| --- | --- | --- | --- | --- | --- |
| Shape | – | LV | ED | Elongation | Shape |
| shape | ­ | LV | ES | Elongation | Shape |
| shape | – | RV | ED | Elongation | Shape |
| shape | – | RV | ES | Elongation | Shape |
| shape | – | LV | ED | Flatness | Shape |
| shape | – | LV | ES | Flatness | Shape |
| shape | – | RV | ED | Flatness | Shape |
| shape | – | RV | ES | Flatness | Shape |
| shape | – | LV | ED | LeastAxis | Size |
| shape | – | LV | ES | LeastAxis | Size |
| shape | – | RV | ED | LeastAxis | Size |
| shape | – | RV | ES | LeastAxis | Size |
| shape | – | LV | ED | MajorAxis | Size |
| shape | – | LV | ES | MajorAxis | Size |
| shape | – | RV | ED | MajorAxis | Size |
| shape | – | RV | ES | MajorAxis | Size |
| shape | – | LV | ED | Max2DdiameterColumn | Size |
| shape | – | LV | ES | Max2DdiameterColumn | Size |
| shape | – | RV | ED | Max2DdiameterColumn | Size |
| shape | – | RV | ES | Max2DdiameterColumn | Size |
| shape | – | LV | ED | Max2DdiameterRow | Size |
| shape | – | LV | ES | Max2DdiameterRow | Size |
| shape | – | RV | ED | Max2DdiameterRow | Size |
| shape | – | RV | ES | Max2DdiameterRow | Size |
| shape | – | LV | ED | Max2DdiameterSlice | Size |
| shape | – | LV | ES | Max2DdiameterSlice | Size |
| shape | – | RV | ED | Max2DdiameterSlice | Size |
| shape | – | RV | ES | Max2DdiameterSlice | Size |
| shape | – | LV | ED | Max3Ddiameter | Size |
| shape | – | LV | ES | Max3Ddiameter | Size |
| shape | – | RV | ED | Max3Ddiameter | Size |
| shape | – | RV | ES | Max3Ddiameter | Size |
| shape | – | LV | ED | MinorAxis | Size |
| shape | – | LV | ES | MinorAxis | Size |
| shape | – | RV | ED | MinorAxis | Size |
| shape | – | RV | ES | MinorAxis | Size |
| shape | – | LV | ED | Sphericity | Shape |
| shape | – | LV | ES | Sphericity | Shape |
| shape | – | RV | ED | Sphericity | Shape |
| shape | – | RV | ES | Sphericity | Shape |
| shape | – | LV | ED | SurfaceArea | Size |
| shape | – | LV | ES | SurfaceArea | Size |
| shape | – | RV | ED | SurfaceArea | Size |
| shape | – | RV | ES | SurfaceArea | Size |
| shape | – | LV | ED | SurfaceAreatoVolumeRatio | Shape |
| shape | – | LV | ES | SurfaceAreatoVolumeRatio | Shape |
| shape | – | RV | ED | SurfaceAreatoVolumeRatio | Shape |
| shape | – | RV | ES | SurfaceAreatoVolumeRatio | Shape |
| shape | – | LV | ED | Volume | Size |
| shape | – | LV | ES | Volume | Size |
| shape | – | RV | ED | Volume | Size |
| shape | – | RV | ES | Volume | Size |
| first-order | – | MYO | ED | Energy | Global Intensity |
| first-order | – | MYO | ES | Energy | Global Intensity |
| first-order | – | MYO | ED | Entropy | Global Variance |
| first-order | – | MYO | ES | Entropy | Global Intensity |
| first-order | – | MYO | ED | InterquartileRange | Global Variance |
| first-order | – | MYO | ES | InterquartileRange | Global Intensity |
| first-order | – | MYO | ED | Kurtosis | Shape |
| first-order | – | MYO | ES | Kurtosis | Shape |
| first-order | – | MYO | ED | Maximum | Global Intensity |
| first-order | – | MYO | ES | Maximum | Shape |
| first-order | – | MYO | ED | Mean | Global Intensity |
| first-order | – | MYO | ES | Mean | Global Intensity |
| first-order | – | MYO | ED | MeanAbsoluteDeviation | Global Variance |
| first-order | – | MYO | ES | MeanAbsoluteDeviation | Global Intensity |
| first-order | – | MYO | ED | Median | Global Intensity |
| first-order | – | MYO | ES | Median | Global Intensity |
| first-order | – | MYO | ED | Minimum | Shape |
| first-order | – | MYO | ES | Minimum | Shape |
| first-order | – | MYO | ED | Percentile10 | Global Intensity |
| first-order | – | MYO | ES | Percentile10 | Global Intensity |
| first-order | – | MYO | ED | Percentile90 | Global Intensity |
| first-order | – | MYO | ES | Percentile90 | Global Intensity |
| first-order | – | MYO | ED | Range | Global Intensity |
| first-order | – | MYO | ES | Range | Shape |
| first-order | – | MYO | ED | RobustMeanAbsoluteDeviation | Global Variance |
| first-order | – | MYO | ES | RobustMeanAbsoluteDeviation | Global Intensity |
| first-order | – | MYO | ED | RootMeanSquared | Global Intensity |
| first-order | – | MYO | ES | RootMeanSquared | Global Intensity |
| first-order | – | MYO | ED | Skewness | Shape |
| first-order | – | MYO | ES | Skewness | Shape |
| first-order | – | MYO | ED | TotalEnergy | Global Intensity |
| first-order | – | MYO | ES | TotalEnergy | Global Intensity |
| first-order | – | MYO | ED | Uniformity | Local Uniformity |
| first-order | – | MYO | ES | Uniformity | Local Uniformity |
| first-order | – | MYO | ED | Variance | Global Variance |
| first-order | – | MYO | ES | Variance | Global Intensity |
| texture | glcm | MYO | ED | Autocorrelation | Global Intensity |
| texture | glcm | MYO | ES | Autocorrelation | Global Intensity |
| texture | glcm | MYO | ED | ClusterProminence | Shape |
| texture | glcm | MYO | ES | ClusterProminence | Shape |
| texture | glcm | MYO | ED | ClusterShade | Shape |
| texture | glcm | MYO | ES | ClusterShade | Shape |
| texture | glcm | MYO | ED | ClusterTendency | Global Variance |
| texture | glcm | MYO | ES | ClusterTendency | Global Intensity |
| texture | glcm | MYO | ED | Contrast | Global Variance |
| texture | glcm | MYO | ES | Contrast | Global Variance |
| texture | glcm | MYO | ED | Correlation | Global Intensity |
| texture | glcm | MYO | ES | Correlation | Local Uniformity |
| texture | glcm | MYO | ED | DifferenceAverage | Global Variance |
| texture | glcm | MYO | ES | DifferenceAverage | Global Variance |
| texture | glcm | MYO | ED | DifferenceEntropy | Global Variance |
| texture | glcm | MYO | ES | DifferenceEntropy | Global Variance |
| texture | glcm | MYO | ED | DifferenceVariance | Global Variance |
| texture | glcm | MYO | ES | DifferenceVariance | Global Variance |
| texture | glcm | MYO | ED | InformalMeasureofCorrelation1 | Shape |
| texture | glcm | MYO | ES | InformalMeasureofCorrelation1 | Shape |
| texture | glcm | MYO | ED | InformalMeasureofCorrelation2 | Global Intensity |
| texture | glcm | MYO | ES | InformalMeasureofCorrelation2 | Local Uniformity |
| texture | glcm | MYO | ED | InverseDifference | Local Uniformity |
| texture | glcm | MYO | ES | InverseDifference | Local Uniformity |
| texture | glcm | MYO | ED | InverseDifferenceMoment | Local Uniformity |
| texture | glcm | MYO | ES | InverseDifferenceMoment | Local Uniformity |
| texture | glcm | MYO | ED | InverseDifferenceMomentNormalized | Local Uniformity |
| texture | glcm | MYO | ES | InverseDifferenceMomentNormalized | Local Uniformity |
| texture | glcm | MYO | ED | InverseDifferenceNormalized | Local Uniformity |
| texture | glcm | MYO | ES | InverseDifferenceNormalized | Local Uniformity |
| texture | glcm | MYO | ED | InverseVariance | Global Variance |
| texture | glcm | MYO | ES | InverseVariance | Global Variance |
| texture | glcm | MYO | ED | JointAverage | Global Intensity |
| texture | glcm | MYO | ES | JointAverage | Global Intensity |
| texture | glcm | MYO | ED | JointEnergy | Local Uniformity |
| texture | glcm | MYO | ES | JointEnergy | Local Uniformity |
| texture | glcm | MYO | ED | JointEntropy | Global Variance |
| texture | glcm | MYO | ES | JointEntropy | Global Intensity |
| texture | glcm | MYO | ED | MaximumProbability | Local Uniformity |
| texture | glcm | MYO | ES | MaximumProbability | Local Uniformity |
| texture | glcm | MYO | ED | SumAverage | Global Intensity |
| texture | glcm | MYO | ES | SumAverage | Global Intensity |
| texture | glcm | MYO | ED | SumEntropy | Global Variance |
| texture | glcm | MYO | ES | SumEntropy | Global Intensity |
| texture | glcm | MYO | ED | SumofSquares | Global Variance |
| texture | glcm | MYO | ES | SumofSquares | Global Intensity |
| texture | gldm | MYO | ED | DependenceEntropy | Global Intensity |
| texture | gldm | MYO | ES | DependenceEntropy | Local Uniformity |
| texture | gldm | MYO | ED | DependenceNonUniformity | Size |
| texture | gldm | MYO | ES | DependenceNonUniformity | Local Uniformity |
| texture | gldm | MYO | ED | DependenceNonUniformityNormalized | Global Variance |
| texture | gldm | MYO | ES | DependenceNonUniformityNormalized | Global Variance |
| texture | gldm | MYO | ED | DependenceVariance | Local Uniformity |
| texture | gldm | MYO | ES | DependenceVariance | Local Uniformity |
| texture | gldm | MYO | ED | GrayLevelNonUniformity | Local Uniformity |
| texture | gldm | MYO | ES | GrayLevelNonUniformity | Local Uniformity |
| texture | gldm | MYO | ED | GrayLevelVariance | Global Variance |
| texture | gldm | MYO | ES | GrayLevelVariance | Global Intensity |
| texture | gldm | MYO | ED | HighGrayLevelEmphasis | Global Intensity |
| texture | gldm | MYO | ES | HighGrayLevelEmphasis | Global Intensity |
| texture | gldm | MYO | ED | LargeDependenceEmphasis | Local Uniformity |
| texture | gldm | MYO | ES | LargeDependenceEmphasis | Local Uniformity |
| texture | gldm | MYO | ED | LargeDependenceHighGrayLevelEmphasis | Local Uniformity |
| texture | gldm | MYO | ES | LargeDependenceHighGrayLevelEmphasis | Local Uniformity |
| texture | gldm | MYO | ED | LargeDependenceLowGrayLevelEmphasis | Local Dimness |
| texture | gldm | MYO | ES | LargeDependenceLowGrayLevelEmphasis | Local Dimness |
| texture | gldm | MYO | ED | LowGrayLevelEmphasis | Local Dimness |
| texture | gldm | MYO | ES | LowGrayLevelEmphasis | Local Dimness |
| texture | gldm | MYO | ED | SmallDependenceEmphasis | Global Variance |
| texture | gldm | MYO | ES | SmallDependenceEmphasis | Global Variance |
| texture | gldm | MYO | ED | SmallDependenceHighGrayLevelEmphasis | Global Intensity |
| texture | gldm | MYO | ES | SmallDependenceHighGrayLevelEmphasis | Global Intensity |
| texture | gldm | MYO | ED | SmallDependenceLowGrayLevelEmphasis | Local Dimness |
| texture | gldm | MYO | ES | SmallDependenceLowGrayLevelEmphasis | Local Dimness |
| texture | glrlm | MYO | ED | GrayLevelNonUniformity | Local Uniformity |
| texture | glrlm | MYO | ES | GrayLevelNonUniformity | Local Uniformity |
| texture | glrlm | MYO | ED | GrayLevelNonUniformityNormalized | Local Uniformity |
| texture | glrlm | MYO | ES | GrayLevelNonUniformityNormalized | Local Uniformity |
| texture | glrlm | MYO | ED | GrayLevelVariance | Global Variance |
| texture | glrlm | MYO | ES | GrayLevelVariance | Global Intensity |
| texture | glrlm | MYO | ED | HighGrayLevelRunEmphasis | Global Intensity |
| texture | glrlm | MYO | ES | HighGrayLevelRunEmphasis | Global Intensity |
| texture | glrlm | MYO | ED | LongRunEmphasis | Local Uniformity |
| texture | glrlm | MYO | ES | LongRunEmphasis | Local Uniformity |
| texture | glrlm | MYO | ED | LongRunHighGrayLevelEmphasis | Local Uniformity |
| texture | glrlm | MYO | ES | LongRunHighGrayLevelEmphasis | Local Uniformity |
| texture | glrlm | MYO | ED | LongRunLowGrayLevelEmphasis | Local Dimness |
| texture | glrlm | MYO | ES | LongRunLowGrayLevelEmphasis | Local Dimness |
| texture | glrlm | MYO | ED | LowGrayLevelRunEmphasis | Local Dimness |
| texture | glrlm | MYO | ES | LowGrayLevelRunEmphasis | Local Dimness |
| texture | glrlm | MYO | ED | RunEntropy | Global Intensity |
| texture | glrlm | MYO | ES | RunEntropy | Local Uniformity |
| texture | glrlm | MYO | ED | RunLengthNonUniformity | Size |
| texture | glrlm | MYO | ES | RunLengthNonUniformity | Size |
| texture | glrlm | MYO | ED | RunLengthNonUniformityNormalized | Global Variance |
| texture | glrlm | MYO | ES | RunLengthNonUniformityNormalized | Global Variance |
| texture | glrlm | MYO | ED | RunPercentage | Global Variance |
| texture | glrlm | MYO | ES | RunPercentage | Global Variance |
| texture | glrlm | MYO | ED | RunVariance | Local Uniformity |
| texture | glrlm | MYO | ES | RunVariance | Local Uniformity |
| texture | glrlm | MYO | ED | ShortRunEmphasis | Global Variance |
| texture | glrlm | MYO | ES | ShortRunEmphasis | Global Variance |
| texture | glrlm | MYO | ED | ShortRunHighGrayLevelEmphasis | Global Intensity |
| texture | glrlm | MYO | ES | ShortRunHighGrayLevelEmphasis | Global Intensity |
| texture | glrlm | MYO | ED | ShortRunLowGrayLevelEmphasis | Local Dimness |
| texture | glrlm | MYO | ES | ShortRunLowGrayLevelEmphasis | Local Dimness |
| texture | glszm | MYO | ED | GrayLevelNonUniformity | Size |
| texture | glszm | MYO | ES | GrayLevelNonUniformity | Size |
| texture | glszm | MYO | ED | GrayLevelNonUniformityNormalized | Local Uniformity |
| texture | glszm | MYO | ES | GrayLevelNonUniformityNormalized | Local Uniformity |
| texture | glszm | MYO | ED | GrayLevelVariance | Global Variance |
| texture | glszm | MYO | ES | GrayLevelVariance | Shape |
| texture | glszm | MYO | ED | HighGrayLevelZoneEmphasis | Global Intensity |
| texture | glszm | MYO | ES | HighGrayLevelZoneEmphasis | Global Intensity |
| texture | glszm | MYO | ED | LargeAreaEmphasis | Local Uniformity |
| texture | glszm | MYO | ES | LargeAreaEmphasis | Local Uniformity |
| texture | glszm | MYO | ED | LargeAreaHighGrayLevelEmphasis | Local Uniformity |
| texture | glszm | MYO | ES | LargeAreaHighGrayLevelEmphasis | Local Uniformity |
| texture | glszm | MYO | ED | LargeAreaLowGrayLevelEmphasis | Local Dimness |
| texture | glszm | MYO | ES | LargeAreaLowGrayLevelEmphasis | Local Dimness |
| texture | glszm | MYO | ED | LowGrayLevelZoneEmphasis | Local Dimness |
| texture | glszm | MYO | ES | LowGrayLevelZoneEmphasis | Local Dimness |
| texture | glszm | MYO | ED | SizeZoneNonUniformity | Size |
| texture | glszm | MYO | ES | SizeZoneNonUniformity | Size |
| texture | glszm | MYO | ED | SizeZoneNonUniformityNormalized | Shape |
| texture | glszm | MYO | ES | SizeZoneNonUniformityNormalized | Shape |
| texture | glszm | MYO | ED | SmallAreaEmphasis | Shape |
| texture | glszm | MYO | ES | SmallAreaEmphasis | Shape |
| texture | glszm | MYO | ED | SmallAreaHighGrayLevelEmphasis | Global Intensity |
| texture | glszm | MYO | ES | SmallAreaHighGrayLevelEmphasis | Global Intensity |
| texture | glszm | MYO | ED | SmallAreaLowGrayLevelEmphasis | Local Dimness |
| texture | glszm | MYO | ES | SmallAreaLowGrayLevelEmphasis | Local Dimness |
| texture | glszm | MYO | ED | ZoneEntropy | Global Variance |
| texture | glszm | MYO | ES | ZoneEntropy | Global Intensity |
| texture | glszm | MYO | ED | ZonePercentage | Global Variance |
| texture | glszm | MYO | ES | ZonePercentage | Global Variance |
| texture | glszm | MYO | ED | ZoneVariance | Local Uniformity |
| texture | glszm | MYO | ES | ZoneVariance | Local Uniformity |
| texture | ngtdm | MYO | ED | Busyness | Local Dimness |
| texture | ngtdm | MYO | ES | Busyness | Local Dimness |
| texture | ngtdm | MYO | ED | Coarseness | Shape |
| texture | ngtdm | MYO | ES | Coarseness | Shape |
| texture | ngtdm | MYO | ED | Complexity | Global Intensity |
| texture | ngtdm | MYO | ES | Complexity | Shape |
| texture | ngtdm | MYO | ED | Contrast | Global Variance |
| texture | ngtdm | MYO | ES | Contrast | Global Variance |
| texture | ngtdm | MYO | ED | Strength | Global Intensity |
| texture | ngtdm | MYO | ES | Strength | Shape |

***Supplementary Table 1.*** *ED: end-diastole, ES: end-systole; MYO: myocardium; LV: left ventricle; RV: right ventricle.*

**Supplementary Table 2. Approach to ascertainment of cardiovascular disease**

| **Source** | **UK Biobank Field** | **ICD code** | | **Description** | |
| --- | --- | --- | --- | --- | --- |
| **Ischaemic heart disease** | | |  | |  |
| Self-report | 20002 |  | | angina | |
|  |  |  | | heart attack/myocardial infarction | |
| Algorithm | 42000 |  | | Date of myocardial infarction | |
| ICD10 | 41270, 41280, 41259, 41234 | I20 | | I20 Angina pectoris | |
|  |  | I21 | | I21 Acute myocardial infarction | |
|  |  | I22 | | I22 Subsequent myocardial infarction | |
|  |  | I23 | | I23 Certain current complications following acute myocardial infarction | |
|  |  | I24 | | I24 Other acute ischaemic heart diseases | |
|  |  | I25 | | I25 Chronic ischaemic heart disease | |
| First occurrences | 131296 |  | | Angina pectoris | |
|  | 131298 |  | | Acute myocardial infarction | |
|  | 131300 |  | | Subsequent myocardial infarction | |
|  | 131302 |  | | Certain current complications following acute myocardial infarction | |
|  | 131304 |  | | Other acute ischaemic heart diseases | |
|  | 131306 |  | | Chronic ischaemic heart disease | |
| Diagnosed by doctor | 6150 | 2 | | Angina | |
|  | 3627 |  | | Age angina diagnosed | |
|  | 6150 | 1 | | Heart attack | |
|  | 3894 |  | | Age heart attack diagnosed | |
| ICD9 | 41271, 41281, 41259, 41234 | 410 | | 410 Acute myocardial infarction | |
|  |  | 411 | | 411 Other acute and subacute forms of ischaemic heart disease | |
|  |  | 412 | | 412 Old myocardial infarction | |
| **Valvular heart disease** | | |  | |  |
| Self-report | 20002 |  | | mitral stenosis | |
|  |  |  | | mitral valve disease | |
|  |  |  | | heart valve problem/heart murmur | |
|  |  |  | | mitral regurgitation / incompetence | |
|  |  |  | | aortic valve disease | |
|  |  |  | | aortic stenosis | |
|  |  |  | | aortic regurgitation / incompetence | |
| ICD10 | 41270, 41280, 41259, 41234 | I340 | | I34.0 Mitral (valve) insufficiency | |
|  |  | I342 | | I34.2 Nonrheumatic mitral (valve) stenosis | |
|  |  | I348 | | I34.8 Other nonrheumatic mitral valve disorders | |
|  |  | I349 | | I34.9 Nonrheumatic mitral valve disorder, unspecified | |
|  |  | I35 | | I35 Nonrheumatic aortic valve disorders | |
|  |  | I36 | | I36 Nonrheumatic tricuspid valve disorders | |
|  |  | I37 | | I37 Pulmonary valve disorders | |
|  |  | I38 | | I38 Endocarditis, valve unspecified | |
|  |  | I390 | | I39.0 Mitral valve disorders in diseases classified elsewhere | |
|  |  | I391 | | I39.1 Aortic valve disorders in diseases classified elsewhere | |
|  |  | I393 | | I39.3 Pulmonary valve disorders in diseases classified elsewhere | |
|  |  | I394 | | I39.4 Multiple valve disorders in diseases classified elsewhere | |
|  |  | I398 | | I39.8 Endocarditis, valve unspecified, in diseases classified elsewhere | |
| First occurrences | 131322 |  | | Nonrheumatic mitral valve disorders | |
|  | 131324 |  | | Nonrheumatic aortic valve disorders | |
|  | 131326 |  | | Nonrheumatic tricuspid valve disorders | |
|  | 131328 |  | | Pulmonary valve disorders | |
|  | 131330 |  | | Endocarditis, valve unspecified | |
|  | 131332 |  | | Endocarditis and heart valve disorders in diseases classified elsewhere | |
| ICD10 | 41270, 41280, 41259, 41234 | I05 | | I05 Rheumatic mitral valve diseases | |
|  |  | I06 | | I06 Rheumatic aortic valve diseases | |
|  |  | I07 | | I07 Rheumatic tricuspid valve diseases | |
|  |  | I08 | | I08 Multiple valve diseases | |
| First occurrences | 131276 |  | | Rheumatic mitral valve diseases | |
|  | 131278 |  | | Rheumatic aortic valve diseases | |
|  | 131280 |  | | Rheumatic tricuspid valve diseases | |
|  | 131282 |  | | Multiple valve diseases | |
| **Non-ischaemic cardiomyopathies** | | |  | |  |
| Self-report | 20002 |  | | cardiomyopathy | |
|  |  |  | | hypertrophic cardiomyopathy (hcm / hocm) | |
| ICD10 | 41270, 41280, 41259, 41234 | I42 | | I42 Cardiomyopathy | |
|  |  | I43 | | I43 Cardiomyopathy in diseases classified elsewhere | |
| First occurrences | 131338 |  | | Cardiomyopathy | |
|  | 131340 |  | | Cardiomyopathy in diseases classified elsewhere | |
| ICD10 | 41270, 41280, 41259, 41234 | I11 | | I11 Hypertensive heart disease | |
|  |  | I13 | | I13 Hypertensive heart and renal disease | |
| First occurrences | 131288 |  | | Hypertensive heart disease | |
|  | 131292 |  | | Hypertensive heart and renal disease | |
| **Cardiac arrhythmia** | | |  | |  |
| Self-report | 20002 |  | | sick sinus syndrome | |
|  |  |  | | svt / supraventricular tachycardia | |
|  |  |  | | atrial fibrillation | |
|  |  |  | | atrial flutter | |
|  |  |  | | heart arrhythmia | |
|  |  |  | | irregular heart beat | |
| ICD10 | 41270, 41280, 41259, 41234 | I441 | | I44.1 Atrioventricular block, second degree | |
|  |  | I442 | | I44.2 Atrioventricular block, complete | |
|  |  | I453 | | I45.3 Trifascicular block | |
|  |  | I456 | | I45.6 Preexcitation syndrome | |
|  |  | I460 | | I46.0 Cardiac arrest with successful resuscitation | |
|  |  | I461 | | I46.1 Sudden cardiac death, so described | |
|  |  | I469 | | I46.9 Cardiac arrest, unspecified | |
|  |  | I470 | | I47.0 Reentry ventricular arrhythmia | |
|  |  | I471 | | I47.1 Supraventricular tachycardia | |
|  |  | I472 | | I47.2 Ventricular tachycardia | |
|  |  | I479 | | I47.9 Paroxysmal tachycardia, unspecified | |
|  |  | I480 | | I48.0 Paroxysmal atrial fibrillation | |
|  |  | I481 | | I48.1 Persistent atrial fibrillation | |
|  |  | I482 | | I48.2 Chronic atrial fibrillation | |
|  |  | I483 | | I48.3 Typical atrial flutter | |
|  |  | I484 | | I48.4 Atypical atrial flutter | |
|  |  | I489 | | I48.9 Atrial fibrillation and atrial flutter, unspecified | |
|  |  | I490 | | I49.0 Ventricular fibrillation and flutter | |
|  |  | I495 | | I49.5 Sick sinus syndrome | |
| First occurrences | 131346 |  | | Cardiac arrest | |
|  | 131348 |  | | Paroxysmal tachycardia | |
|  | 131350 |  | | Atrial fibrillation and flutter | |

***Supplementary Table 2.*** *ICD: international classification of disease*

**Supplementary Table 3. Approach to ascertainment of vascular risk factors**

| **Source** | **UK Field ID** | **Code** | **Description** |
| --- | --- | --- | --- |
| **Diabetes** |  |  |  |
| Diagnosed by doctor | 2443 |  | Diabetes diagnosed by doctor |
|  | [2976](https://biobank.ndph.ox.ac.uk/showcase/field.cgi?id=2976) |  | Age diabetes diagnosed by doctor |
| Self-report | 20002 |  | diabetes |
|  |  |  | type 1 diabetes |
|  |  |  | type 2 diabetes |
| ICD10 | 41270, 41280, 41259, 41234 | E10 | Type 1 diabetes mellitus |
|  |  | E11 | Type 2 diabetes mellitus |
|  |  | E13 | Other specified diabetes mellitus |
|  |  | E14 | Unspecified diabetes mellitus |
|  |  | G590 | Diabetic mononeuropathy |
|  |  | G632 | Diabetic polyneuropathy |
|  |  | H280 | Diabetic cataract |
|  |  | H360 | Diabetic retinopathy |
|  |  | M142 | Diabetic arthropathy |
|  |  | N083 | Glomerular disorders in diabetes mellitus |
|  |  | O240 | Diabetes mellitus in pregnancy: Pre-existing type 1 diabetes mellitus |
|  |  | O241 | Diabetes mellitus in pregnancy: Pre-existing type 2 diabetes mellitus |
|  |  | O243 | Diabetes mellitus in pregnancy: Pre-existing diabetes mellitus, unspecified |
|  |  | O244 | Diabetes mellitus arising in pregnancy |
|  |  | O249 | Diabetes mellitus in pregnancy, unspecified |
|  |  | Y423 | Insulin and oral hypoglycaemic [antidiabetic] drugs |
| First occurrences | 130706 |  | Insulin-dependent diabetes mellitus |
|  | 130708 |  | Non-insulin-dependent diabetes mellitus |
|  | 130712 |  | Other specified diabetes mellitus |
|  | 130714 |  | Unspecified diabetes mellitus |
| Medications | 6177, 6153 | 3 | Insulin |
| Biochemistry | 30750 | > 48 | Glycated haemoglobin (HbA1c) |
| **High cholesterol** |  |  |  |
| ICD10 | 41270, 41280, 41259, 41234 | E780 | Pure hypercholesterolaemia |
|  |  | E782 | Mixed hyperlipidaemia |
|  |  | E783 | Hyperchylomicronaemia |
|  |  | E784 | Other hyperlipidaemia |
|  |  | E785 | Hyperlipidaemia, unspecified |
| First occurrences | 130814 |  | Disorders of lipoprotein metabolism and other lipidaemias |
| Medications | 6177, 6153 | 1 | Cholesterol lowering medication |
| Biochemistry | 30690 | > 7 | Cholesterol |
| **Hypertension** |  |  |  |
| Self-report | 20002 |  | essential hypertension |
|  |  |  | hypertension |
| ICD10 | 41270, 41280, 41259, 41234 | I10 | Essential (primary) hypertension |
| Medications | 6177, 6153 | 2 | Blood pressure medication |
| First occurrences | 131286 |  | Essential (primary) hypertension |
| Diagnosed by doctor | 6150 | 4 | High blood pressure |
|  | 2966 |  | Age high blood pressure diagnosed |
| **Smoking** |  |  |  |
| Self-report | 1239 | 1 or 2 | Current tobacco smoking |
|  | 20116 | 2 | Smoking status |

***Supplementary Table 3.*** *ICD: international classification of disease*

**Supplementary Table 4. Associations of sex and age with radiomics features from linear regression models in the healthy subset**

|  |  | **Beta** | | | | **Adjusted p-value** | | | |
| --- | --- | --- | --- | --- | --- | --- | --- | --- | --- |
| **Feature** | **Cluster** | **Sex** | **BSA** | **Age** | **Sex***  **Age** | **Sex** | **BSA** | **Age** | **Sex*age** |
| InterquartileRange MYO ES | Global Intensity | -0.051 | -0.042 | -0.145 | 0.034 | 1.000E+00 | 2.190E-02 | 5.518E-68 | 1.000E+00 |
| RobustMeanAbsoluteDeviation MYO ES | Global Intensity | -0.071 | -0.038 | -0.137 | 0.036 | 3.094E-01 | 9.159E-02 | 1.434E-60 | 1.000E+00 |
| MeanAbsoluteDeviation MYO ES | Global Intensity | -0.228 | -0.044 | -0.099 | 0.039 | 8.134E-23 | 1.040E-02 | 5.672E-32 | 1.000E+00 |
| SumEntropy glcm MYO ES | Global Intensity | -0.259 | -0.063 | -0.093 | 0.028 | 5.997E-30 | 8.212E-07 | 1.612E-28 | 1.000E+00 |
| Entropy MYO ES | Global Intensity | -0.349 | -0.095 | -0.093 | 0.035 | 2.340E-56 | 7.851E-17 | 3.048E-29 | 1.000E+00 |
| Energy MYO ES | Global Intensity | 0.577 | 0.108 | -0.080 | -0.062 | 1.000E-100 | 6.954E-24 | 4.828E-23 | 1.416E-02 |
| TotalEnergy MYO ES | Global Intensity | 0.542 | 0.182 | -0.068 | -0.055 | 1.000E-100 | 9.963E-72 | 2.800E-17 | 6.870E-02 |
| ClusterTendency glcm MYO ES | Global Intensity | -0.101 | -0.025 | -0.068 | 0.026 | 1.140E-03 | 1.000E+00 | 3.224E-14 | 1.000E+00 |
| Autocorrelation glcm MYO ES | Global Intensity | -0.027 | -0.135 | -0.061 | 0.005 | 1.000E+00 | 1.615E-33 | 1.837E-11 | 1.000E+00 |
| SumAverage glcm MYO ES | Global Intensity | -0.048 | -0.142 | -0.060 | 0.002 | 1.000E+00 | 6.608E-37 | 4.967E-11 | 1.000E+00 |
| JointAverage glcm MYO ES | Global Intensity | -0.048 | -0.142 | -0.060 | 0.002 | 1.000E+00 | 6.608E-37 | 4.967E-11 | 1.000E+00 |
| JointEntropy glcm MYO ES | Global Intensity | -0.465 | -0.079 | -0.058 | 0.041 | 1.000E-100 | 1.090E-11 | 3.020E-11 | 1.000E+00 |
| Variance MYO ES | Global Intensity | -0.304 | -0.037 | -0.053 | 0.040 | 2.326E-41 | 1.461E-01 | 9.966E-09 | 1.000E+00 |
| HighGrayLevelEmphasis gldm MYO ES | Global Intensity | -0.047 | -0.130 | -0.052 | 0.001 | 1.000E+00 | 9.791E-31 | 3.871E-08 | 1.000E+00 |
| GrayLevelVariance gldm MYO ES | Global Intensity | -0.324 | -0.053 | -0.052 | 0.040 | 3.554E-47 | 1.826E-04 | 4.083E-08 | 1.000E+00 |
| GrayLevelVariance glrlm MYO ES | Global Intensity | -0.319 | -0.049 | -0.046 | 0.038 | 8.761E-46 | 1.211E-03 | 2.622E-06 | 1.000E+00 |
| HighGrayLevelRunEmphasis glrlm MYO ES | Global Intensity | -0.039 | -0.116 | -0.045 | 0.001 | 1.000E+00 | 1.952E-24 | 6.886E-06 | 1.000E+00 |
| SumofSquares glcm MYO ES | Global Intensity | -0.283 | -0.041 | -0.042 | 0.034 | 1.086E-35 | 3.124E-02 | 6.545E-05 | 1.000E+00 |
| Median MYO ES | Global Intensity | -0.207 | -0.243 | -0.041 | 0.029 | 2.116E-20 | 1.000E-100 | 2.561E-05 | 1.000E+00 |
| Percentile90 MYO ES | Global Intensity | -0.205 | -0.196 | -0.040 | 0.024 | 2.330E-19 | 1.511E-74 | 8.851E-05 | 1.000E+00 |
| SmallAreaHighGrayLevelEmphasis glszm MYO ES | Global Intensity | -0.220 | -0.064 | -0.033 | 0.040 | 3.452E-21 | 7.867E-07 | 1.030E-02 | 1.000E+00 |
| ZoneEntropy glszm MYO ES | Global Intensity | -0.196 | 0.017 | -0.033 | 0.021 | 2.788E-16 | 1.000E+00 | 1.550E-02 | 1.000E+00 |
| RootMeanSquared MYO ES | Global Intensity | -0.219 | -0.227 | -0.029 | 0.026 | 1.150E-22 | 1.000E-100 | 6.290E-02 | 1.000E+00 |
| ShortRunHighGrayLevelEmphasis glrlm MYO ES | Global Intensity | -0.220 | -0.113 | -0.028 | 0.020 | 1.338E-21 | 8.604E-24 | 1.005E-01 | 1.000E+00 |
| Mean MYO ES | Global Intensity | -0.209 | -0.233 | -0.026 | 0.025 | 1.799E-20 | 1.000E-100 | 1.790E-01 | 1.000E+00 |
| HighGrayLevelZoneEmphasis glszm MYO ES | Global Intensity | -0.203 | -0.051 | -0.016 | 0.024 | 1.027E-17 | 5.193E-04 | 1.000E+00 | 1.000E+00 |
| SmallDependenceHighGrayLevelEmphasis gldm MYO ES | Global Intensity | -0.545 | -0.097 | 0.003 | 0.055 | 1.000E-100 | 1.472E-18 | 1.000E+00 | 9.473E-02 |
| Percentile10 MYO ES | Global Intensity | -0.158 | -0.267 | 0.021 | 0.010 | 1.371E-11 | 1.000E-100 | 1.000E+00 | 1.000E+00 |
| Energy MYO ED | Global Intensity | 0.306 | 0.179 | 0.065 | -0.066 | 9.804E-45 | 2.050E-63 | 1.762E-14 | 6.662E-03 |
| Strength ngtdm MYO ED | Global Intensity | -0.618 | -0.127 | 0.067 | -0.006 | 1.000E-100 | 3.083E-34 | 2.628E-16 | 1.000E+00 |
| Complexity ngtdm MYO ED | Global Intensity | -0.747 | -0.055 | 0.073 | 0.009 | 1.000E-100 | 8.166E-06 | 9.275E-20 | 1.000E+00 |
| Range MYO ED | Global Intensity | -0.689 | -0.056 | 0.078 | 0.016 | 1.000E-100 | 8.572E-06 | 3.635E-22 | 1.000E+00 |
| Maximum MYO ED | Global Intensity | -0.668 | -0.057 | 0.079 | 0.022 | 1.000E-100 | 4.771E-06 | 2.165E-22 | 1.000E+00 |
| SmallDependenceHighGrayLevelEmphasis gldm MYO ED | Global Intensity | -0.901 | -0.126 | 0.080 | -0.027 | 1.000E-100 | 4.785E-40 | 6.271E-28 | 1.000E+00 |
| TotalEnergy MYO ED | Global Intensity | 0.256 | 0.267 | 0.083 | -0.056 | 1.047E-32 | 1.000E-100 | 6.977E-25 | 7.001E-02 |
| SmallAreaHighGrayLevelEmphasis glszm MYO ED | Global Intensity | -0.594 | -0.112 | 0.083 | -0.028 | 1.000E-100 | 5.933E-26 | 1.763E-25 | 1.000E+00 |
| Correlation glcm MYO ED | Global Intensity | 0.214 | 0.127 | 0.090 | -0.075 | 1.257E-20 | 1.775E-30 | 1.017E-26 | 6.724E-04 |
| JointAverage glcm MYO ED | Global Intensity | -0.197 | -0.124 | 0.096 | -0.049 | 3.433E-17 | 7.394E-29 | 3.218E-30 | 6.079E-01 |
| SumAverage glcm MYO ED | Global Intensity | -0.197 | -0.124 | 0.096 | -0.049 | 3.433E-17 | 7.394E-29 | 3.218E-30 | 6.079E-01 |
| Autocorrelation glcm MYO ED | Global Intensity | -0.219 | -0.122 | 0.100 | -0.053 | 1.708E-21 | 6.999E-28 | 1.811E-33 | 2.371E-01 |
| HighGrayLevelZoneEmphasis glszm MYO ED | Global Intensity | -0.574 | -0.094 | 0.102 | -0.043 | 1.000E-100 | 1.020E-17 | 9.327E-38 | 1.000E+00 |
| HighGrayLevelRunEmphasis glrlm MYO ED | Global Intensity | -0.376 | -0.101 | 0.113 | -0.064 | 3.409E-66 | 1.566E-19 | 7.587E-44 | 1.120E-02 |
| ShortRunHighGrayLevelEmphasis glrlm MYO ED | Global Intensity | -0.551 | -0.107 | 0.115 | -0.053 | 1.000E-100 | 1.537E-23 | 8.873E-48 | 1.425E-01 |
| HighGrayLevelEmphasis gldm MYO ED | Global Intensity | -0.349 | -0.110 | 0.116 | -0.065 | 3.493E-57 | 3.340E-23 | 9.534E-46 | 8.706E-03 |
| Percentile10 MYO ED | Global Intensity | 0.004 | -0.182 | 0.117 | -0.037 | 1.000E+00 | 1.838E-62 | 1.598E-45 | 1.000E+00 |
| RunEntropy glrlm MYO ED | Global Intensity | -0.131 | 0.103 | 0.120 | -0.128 | 6.496E-07 | 5.218E-19 | 4.839E-46 | 1.091E-12 |
| InformalMeasureofCorrelation2 glcm MYO ED | Global Intensity | -0.152 | 0.001 | 0.146 | -0.072 | 1.141E-09 | 1.000E+00 | 1.233E-68 | 2.546E-03 |
| Percentile90 MYO ED | Global Intensity | -0.770 | -0.114 | 0.146 | -0.032 | 1.000E-100 | 3.416E-30 | 1.577E-87 | 1.000E+00 |
| RootMeanSquared MYO ED | Global Intensity | -0.541 | -0.155 | 0.154 | -0.037 | 1.000E-100 | 6.254E-52 | 2.148E-90 | 1.000E+00 |
| Mean MYO ED | Global Intensity | -0.465 | -0.166 | 0.155 | -0.038 | 1.000E-100 | 2.324E-58 | 2.165E-89 | 1.000E+00 |
| Median MYO ED | Global Intensity | -0.341 | -0.196 | 0.166 | -0.045 | 4.091E-58 | 1.329E-79 | 9.465E-100 | 8.186E-01 |
| DependenceEntropy gldm MYO ED | Global Intensity | -0.012 | 0.114 | 0.176 | -0.082 | 1.000E+00 | 5.237E-24 | 1.000E-100 | 8.465E-05 |
| Contrast ngtdm MYO ES | Global Variance | -0.303 | -0.062 | -0.014 | 0.025 | 3.680E-41 | 1.636E-06 | 1.000E+00 | 1.000E+00 |
| DependenceNonUniformityNormalized gldm MYO ES | Global Variance | -0.471 | -0.110 | -0.009 | 0.041 | 1.000E-100 | 6.744E-24 | 1.000E+00 | 1.000E+00 |
| InverseVariance glcm MYO ES | Global Variance | -0.731 | -0.067 | 0.005 | 0.042 | 1.000E-100 | 3.745E-09 | 1.000E+00 | 1.000E+00 |
| RunPercentage glrlm MYO ES | Global Variance | -0.822 | -0.116 | 0.007 | 0.066 | 1.000E-100 | 3.245E-31 | 1.000E+00 | 1.004E-03 |
| RunLengthNonUniformityNormalized glrlm MYO ES | Global Variance | -0.799 | -0.086 | 0.023 | 0.060 | 1.000E-100 | 2.697E-16 | 4.388E-01 | 1.148E-02 |
| ShortRunEmphasis glrlm MYO ES | Global Variance | -0.838 | -0.083 | 0.023 | 0.067 | 1.000E-100 | 1.915E-15 | 3.124E-01 | 1.077E-03 |
| DifferenceEntropy glcm MYO ES | Global Variance | -0.783 | -0.085 | 0.024 | 0.059 | 1.000E-100 | 6.676E-16 | 2.790E-01 | 1.619E-02 |
| DifferenceAverage glcm MYO ES | Global Variance | -0.745 | -0.079 | 0.025 | 0.048 | 1.000E-100 | 5.932E-13 | 1.741E-01 | 2.971E-01 |
| SmallDependenceEmphasis gldm MYO ES | Global Variance | -0.844 | -0.102 | 0.029 | 0.074 | 1.000E-100 | 4.660E-24 | 1.152E-02 | 7.559E-05 |
| Contrast glcm MYO ES | Global Variance | -0.656 | -0.070 | 0.034 | 0.045 | 1.000E-100 | 1.011E-09 | 1.899E-03 | 7.422E-01 |
| DifferenceVariance glcm MYO ES | Global Variance | -0.647 | -0.069 | 0.035 | 0.051 | 1.000E-100 | 2.244E-09 | 1.279E-03 | 2.317E-01 |
| ZonePercentage glszm MYO ED | Global Variance | -1.121 | -0.126 | 0.037 | 0.032 | 1.000E-100 | 1.545E-48 | 1.350E-06 | 1.000E+00 |
| ZonePercentage glszm MYO ES | Global Variance | -0.793 | -0.101 | 0.039 | 0.067 | 1.000E-100 | 6.788E-23 | 2.024E-05 | 1.311E-03 |
| SmallDependenceEmphasis gldm MYO ED | Global Variance | -1.138 | -0.135 | 0.041 | 0.041 | 1.000E-100 | 3.457E-57 | 7.909E-09 | 2.585E-01 |
| DifferenceVariance glcm MYO ED | Global Variance | -1.014 | -0.081 | 0.058 | 0.027 | 1.000E-100 | 8.705E-17 | 4.489E-15 | 1.000E+00 |
| RunLengthNonUniformityNormalized glrlm MYO ED | Global Variance | -1.106 | -0.124 | 0.072 | 0.064 | 1.000E-100 | 5.071E-46 | 2.532E-27 | 1.253E-04 |
| ShortRunEmphasis glrlm MYO ED | Global Variance | -1.122 | -0.124 | 0.073 | 0.078 | 1.000E-100 | 1.127E-46 | 3.634E-28 | 1.893E-07 |
| DifferenceEntropy glcm MYO ED | Global Variance | -1.129 | -0.092 | 0.079 | 0.057 | 1.000E-100 | 1.729E-24 | 1.016E-31 | 2.614E-03 |
| Contrast glcm MYO ED | Global Variance | -1.037 | -0.092 | 0.079 | 0.012 | 1.000E-100 | 1.720E-22 | 3.664E-29 | 1.000E+00 |
| Contrast ngtdm MYO ED | Global Variance | -0.938 | -0.051 | 0.081 | -0.012 | 1.000E-100 | 1.555E-05 | 1.354E-27 | 1.000E+00 |
| RunPercentage glrlm MYO ED | Global Variance | -1.095 | -0.132 | 0.083 | 0.073 | 1.000E-100 | 1.820E-52 | 1.506E-36 | 3.148E-06 |
| ZoneEntropy glszm MYO ED | Global Variance | -0.696 | 0.022 | 0.085 | -0.006 | 1.000E-100 | 1.000E+00 | 1.174E-25 | 1.000E+00 |
| DependenceNonUniformityNormalized gldm MYO ED | Global Variance | -0.953 | -0.139 | 0.086 | 0.012 | 1.000E-100 | 2.777E-51 | 8.366E-35 | 1.000E+00 |
| SumofSquares glcm MYO ED | Global Variance | -0.956 | -0.054 | 0.097 | -0.010 | 1.000E-100 | 2.440E-06 | 8.042E-40 | 1.000E+00 |
| DifferenceAverage glcm MYO ED | Global Variance | -1.090 | -0.101 | 0.098 | 0.032 | 1.000E-100 | 8.889E-29 | 1.930E-48 | 1.000E+00 |
| GrayLevelVariance glszm MYO ED | Global Variance | -0.738 | -0.070 | 0.102 | -0.009 | 1.000E-100 | 4.235E-10 | 7.958E-40 | 1.000E+00 |
| ClusterTendency glcm MYO ED | Global Variance | -0.881 | -0.033 | 0.102 | -0.021 | 1.000E-100 | 1.906E-01 | 9.526E-42 | 1.000E+00 |
| GrayLevelVariance glrlm MYO ED | Global Variance | -0.937 | -0.025 | 0.102 | -0.040 | 1.000E-100 | 1.000E+00 | 3.837E-43 | 1.000E+00 |
| Variance MYO ED | Global Variance | -0.955 | -0.026 | 0.106 | -0.041 | 1.000E-100 | 1.000E+00 | 9.117E-47 | 1.000E+00 |
| GrayLevelVariance gldm MYO ED | Global Variance | -0.951 | -0.034 | 0.107 | -0.038 | 1.000E-100 | 8.599E-02 | 1.594E-47 | 1.000E+00 |
| InverseVariance glcm MYO ED | Global Variance | -0.868 | -0.094 | 0.128 | 0.075 | 1.000E-100 | 9.533E-21 | 3.039E-69 | 2.886E-05 |
| MeanAbsoluteDeviation MYO ED | Global Variance | -1.077 | -0.021 | 0.130 | -0.024 | 1.000E-100 | 1.000E+00 | 4.544E-76 | 1.000E+00 |
| JointEntropy glcm MYO ED | Global Variance | -1.042 | -0.081 | 0.133 | 0.023 | 1.000E-100 | 1.802E-17 | 4.203E-83 | 1.000E+00 |
| Entropy MYO ED | Global Variance | -1.057 | -0.075 | 0.138 | 0.002 | 1.000E-100 | 1.244E-14 | 7.028E-90 | 1.000E+00 |
| SumEntropy glcm MYO ED | Global Variance | -0.987 | -0.066 | 0.138 | 0.023 | 1.000E-100 | 2.000E-10 | 6.444E-85 | 1.000E+00 |
| RobustMeanAbsoluteDeviation MYO ED | Global Variance | -1.030 | 0.000 | 0.153 | -0.039 | 1.000E-100 | 1.000E+00 | 2.218E-100 | 1.000E+00 |
| InterquartileRange MYO ED | Global Variance | -0.991 | 0.005 | 0.162 | -0.047 | 1.000E-100 | 1.000E+00 | 1.000E-100 | 2.445E-01 |
| Busyness ngtdm MYO ED | Local Dimness | 0.618 | 0.216 | -0.104 | -0.039 | 1.000E-100 | 1.000E-100 | 1.053E-44 | 1.000E+00 |
| LargeDependenceLowGrayLevelEmphasis gldm MYO ED | Local Dimness | 0.594 | 0.145 | -0.094 | -0.029 | 1.000E-100 | 6.885E-45 | 2.742E-33 | 1.000E+00 |
| LongRunLowGrayLevelEmphasis glrlm MYO ED | Local Dimness | 0.530 | 0.135 | -0.089 | -0.035 | 1.000E-100 | 8.839E-38 | 4.836E-29 | 1.000E+00 |
| LargeAreaLowGrayLevelEmphasis glszm MYO ED | Local Dimness | 0.732 | 0.144 | -0.089 | -0.110 | 1.000E-100 | 1.318E-47 | 6.064E-32 | 8.675E-12 |
| LowGrayLevelEmphasis gldm MYO ED | Local Dimness | 0.010 | 0.097 | -0.076 | 0.050 | 1.000E+00 | 7.070E-17 | 4.249E-18 | 5.169E-01 |
| LowGrayLevelRunEmphasis glrlm MYO ED | Local Dimness | 0.030 | 0.094 | -0.074 | 0.046 | 1.000E+00 | 1.322E-15 | 3.293E-17 | 1.000E+00 |
| ShortRunLowGrayLevelEmphasis glrlm MYO ED | Local Dimness | -0.117 | 0.075 | -0.066 | 0.061 | 2.781E-05 | 1.661E-09 | 2.149E-13 | 4.692E-02 |
| LowGrayLevelZoneEmphasis glszm MYO ED | Local Dimness | 0.218 | 0.079 | -0.055 | 0.036 | 6.130E-21 | 7.302E-11 | 2.288E-09 | 1.000E+00 |
| SmallAreaLowGrayLevelEmphasis glszm MYO ED | Local Dimness | 0.265 | 0.081 | -0.054 | 0.030 | 2.678E-31 | 1.235E-11 | 4.371E-09 | 1.000E+00 |
| SmallDependenceLowGrayLevelEmphasis gldm MYO ED | Local Dimness | -0.534 | -0.003 | -0.040 | 0.087 | 1.000E-100 | 1.000E+00 | 9.110E-05 | 1.009E-05 |
| LargeAreaLowGrayLevelEmphasis glszm MYO ES | Local Dimness | 0.571 | 0.180 | -0.008 | -0.064 | 1.000E-100 | 2.585E-70 | 1.000E+00 | 4.159E-03 |
| Busyness ngtdm MYO ES | Local Dimness | 0.461 | 0.236 | -0.002 | -0.043 | 1.000E-100 | 1.000E-100 | 1.000E+00 | 1.000E+00 |
| SmallAreaLowGrayLevelEmphasis glszm MYO ES | Local Dimness | -0.013 | 0.063 | 0.005 | 0.021 | 1.000E+00 | 1.718E-06 | 1.000E+00 | 1.000E+00 |
| LowGrayLevelZoneEmphasis glszm MYO ES | Local Dimness | -0.024 | 0.062 | 0.006 | 0.012 | 1.000E+00 | 2.796E-06 | 1.000E+00 | 1.000E+00 |
| ShortRunLowGrayLevelEmphasis glrlm MYO ES | Local Dimness | -0.122 | 0.092 | 0.026 | 0.021 | 9.141E-06 | 5.965E-15 | 3.146E-01 | 1.000E+00 |
| SmallDependenceLowGrayLevelEmphasis gldm MYO ES | Local Dimness | -0.471 | 0.013 | 0.027 | 0.053 | 2.094E-100 | 1.000E+00 | 2.031E-01 | 2.162E-01 |
| LowGrayLevelRunEmphasis glrlm MYO ES | Local Dimness | 0.040 | 0.115 | 0.030 | 0.011 | 1.000E+00 | 5.154E-24 | 6.353E-02 | 1.000E+00 |
| LongRunLowGrayLevelEmphasis glrlm MYO ES | Local Dimness | 0.491 | 0.154 | 0.032 | -0.025 | 1.000E-100 | 4.359E-49 | 6.210E-03 | 1.000E+00 |
| LowGrayLevelEmphasis gldm MYO ES | Local Dimness | 0.056 | 0.125 | 0.037 | 0.013 | 1.000E+00 | 2.422E-28 | 1.296E-03 | 1.000E+00 |
| LargeDependenceLowGrayLevelEmphasis gldm MYO ES | Local Dimness | 0.426 | 0.166 | 0.043 | -0.012 | 6.431E-89 | 9.535E-56 | 5.249E-06 | 1.000E+00 |
| Uniformity MYO ED | Local Uniformity | 0.967 | 0.082 | -0.146 | -0.029 | 1.000E-100 | 6.601E-17 | 6.246E-95 | 1.000E+00 |
| InformalMeasureofCorrelation2 glcm MYO ES | Local Uniformity | 0.614 | -0.002 | -0.143 | -0.035 | 1.000E-100 | 1.000E+00 | 1.069E-71 | 1.000E+00 |
| MaximumProbability glcm MYO ED | Local Uniformity | 0.783 | 0.103 | -0.142 | -0.049 | 1.000E-100 | 3.155E-24 | 1.047E-81 | 1.679E-01 |
| Correlation glcm MYO ES | Local Uniformity | 0.705 | 0.049 | -0.141 | -0.034 | 1.000E-100 | 2.473E-04 | 2.871E-74 | 1.000E+00 |
| JointEnergy glcm MYO ED | Local Uniformity | 0.881 | 0.083 | -0.139 | -0.064 | 1.000E-100 | 4.596E-16 | 1.555E-81 | 1.983E-03 |
| GrayLevelNonUniformityNormalized glrlm MYO ED | Local Uniformity | 1.032 | 0.056 | -0.138 | -0.006 | 1.000E-100 | 2.206E-07 | 3.254E-86 | 1.000E+00 |
| GrayLevelNonUniformity gldm MYO ED | Local Uniformity | 1.087 | 0.271 | -0.136 | -0.129 | 1.000E-100 | 1.000E-100 | 1.000E-100 | 1.095E-29 |
| GrayLevelNonUniformity glrlm MYO ED | Local Uniformity | 1.070 | 0.316 | -0.132 | -0.088 | 1.000E-100 | 1.000E-100 | 1.000E-100 | 3.787E-15 |
| InverseDifference glcm MYO ED | Local Uniformity | 1.075 | 0.103 | -0.110 | -0.046 | 1.000E-100 | 9.670E-30 | 1.842E-60 | 1.203E-01 |
| DependenceEntropy gldm MYO ES | Local Uniformity | 0.219 | 0.032 | -0.109 | -0.016 | 4.255E-21 | 6.613E-01 | 9.142E-39 | 1.000E+00 |
| InverseDifferenceMoment glcm MYO ED | Local Uniformity | 1.085 | 0.103 | -0.107 | -0.042 | 1.000E-100 | 5.495E-30 | 5.802E-58 | 2.865E-01 |
| RunEntropy glrlm MYO ES | Local Uniformity | 0.653 | 0.067 | -0.101 | -0.049 | 1.000E-100 | 8.323E-09 | 1.345E-37 | 2.957E-01 |
| ZoneVariance glszm MYO ED | Local Uniformity | 1.041 | 0.172 | -0.099 | -0.154 | 1.000E-100 | 4.880E-90 | 1.119E-52 | 6.999E-32 |
| LargeAreaEmphasis glszm MYO ED | Local Uniformity | 1.040 | 0.171 | -0.098 | -0.153 | 1.000E-100 | 2.050E-88 | 1.449E-51 | 2.308E-31 |
| DependenceNonUniformity gldm MYO ES | Local Uniformity | 0.845 | 0.367 | -0.092 | -0.104 | 1.000E-100 | 1.000E-100 | 3.011E-55 | 4.521E-17 |
| DependenceVariance gldm MYO ED | Local Uniformity | 0.904 | 0.151 | -0.089 | -0.065 | 1.000E-100 | 1.179E-59 | 3.056E-36 | 4.828E-04 |
| LargeDependenceEmphasis gldm MYO ED | Local Uniformity | 1.056 | 0.134 | -0.086 | -0.088 | 1.000E-100 | 7.260E-52 | 8.535E-38 | 2.850E-09 |
| RunVariance glrlm MYO ED | Local Uniformity | 1.044 | 0.142 | -0.086 | -0.119 | 1.000E-100 | 2.311E-58 | 1.397E-37 | 8.059E-18 |
| LongRunEmphasis glrlm MYO ED | Local Uniformity | 1.057 | 0.136 | -0.081 | -0.116 | 1.000E-100 | 7.867E-54 | 1.582E-33 | 6.571E-17 |
| LargeAreaHighGrayLevelEmphasis glszm MYO ES | Local Uniformity | 0.877 | 0.130 | -0.075 | -0.139 | 1.000E-100 | 7.315E-42 | 3.225E-24 | 5.756E-21 |
| InverseDifferenceNormalized glcm MYO ED | Local Uniformity | 0.835 | 0.082 | -0.071 | -0.047 | 1.000E-100 | 5.020E-15 | 7.002E-20 | 2.775E-01 |
| GrayLevelNonUniformityNormalized glszm MYO ED | Local Uniformity | 0.793 | 0.082 | -0.068 | -0.019 | 1.000E-100 | 1.233E-14 | 5.195E-18 | 1.000E+00 |
| LargeAreaHighGrayLevelEmphasis glszm MYO ED | Local Uniformity | 1.053 | 0.135 | -0.068 | -0.144 | 1.000E-100 | 1.688E-52 | 3.402E-23 | 3.273E-26 |
| LargeDependenceHighGrayLevelEmphasis gldm MYO ES | Local Uniformity | 0.459 | -0.069 | -0.064 | -0.054 | 1.838E-94 | 3.757E-08 | 7.693E-13 | 1.840E-01 |
| LongRunHighGrayLevelEmphasis glrlm MYO ES | Local Uniformity | 0.632 | -0.039 | -0.053 | -0.083 | 1.000E-100 | 5.126E-02 | 5.351E-09 | 3.853E-05 |
| InverseDifferenceMomentNormalized glcm MYO ES | Local Uniformity | 0.316 | 0.039 | -0.050 | -0.014 | 1.021E-44 | 7.785E-02 | 1.948E-07 | 1.000E+00 |
| LargeAreaEmphasis glszm MYO ES | Local Uniformity | 0.864 | 0.210 | -0.038 | -0.119 | 1.000E-100 | 1.000E-100 | 2.806E-06 | 1.906E-16 |
| ZoneVariance glszm MYO ES | Local Uniformity | 0.867 | 0.215 | -0.038 | -0.119 | 1.000E-100 | 1.000E-100 | 3.613E-06 | 1.212E-16 |
| InverseDifferenceNormalized glcm MYO ES | Local Uniformity | 0.575 | 0.061 | -0.036 | -0.027 | 1.000E-100 | 9.434E-07 | 9.070E-04 | 1.000E+00 |
| GrayLevelNonUniformity glrlm MYO ES | Local Uniformity | 0.818 | 0.410 | -0.024 | -0.089 | 1.000E-100 | 1.000E-100 | 3.536E-03 | 3.791E-13 |
| InverseDifferenceMomentNormalized glcm MYO ED | Local Uniformity | 0.621 | 0.056 | -0.023 | -0.032 | 1.000E-100 | 9.531E-06 | 5.705E-01 | 1.000E+00 |
| InverseDifferenceMoment glcm MYO ES | Local Uniformity | 0.758 | 0.079 | -0.021 | -0.048 | 1.000E-100 | 3.655E-13 | 1.000E+00 | 3.009E-01 |
| InverseDifference glcm MYO ES | Local Uniformity | 0.759 | 0.078 | -0.019 | -0.048 | 1.000E-100 | 9.924E-13 | 1.000E+00 | 3.454E-01 |
| GrayLevelNonUniformity gldm MYO ES | Local Uniformity | 0.882 | 0.342 | -0.012 | -0.093 | 1.000E-100 | 1.000E-100 | 1.000E+00 | 3.299E-13 |
| LargeDependenceEmphasis gldm MYO ES | Local Uniformity | 0.804 | 0.123 | 0.003 | -0.068 | 1.000E-100 | 4.541E-35 | 1.000E+00 | 7.061E-04 |
| LongRunEmphasis glrlm MYO ES | Local Uniformity | 0.869 | 0.122 | 0.004 | -0.084 | 1.000E-100 | 1.832E-36 | 1.000E+00 | 7.153E-07 |
| RunVariance glrlm MYO ES | Local Uniformity | 0.880 | 0.137 | 0.005 | -0.089 | 1.000E-100 | 3.988E-47 | 1.000E+00 | 5.640E-08 |
| LargeDependenceHighGrayLevelEmphasis gldm MYO ED | Local Uniformity | 0.824 | 0.017 | 0.007 | -0.096 | 1.000E-100 | 1.000E+00 | 1.000E+00 | 4.681E-08 |
| DependenceVariance gldm MYO ES | Local Uniformity | 0.523 | 0.120 | 0.037 | -0.049 | 1.000E-100 | 1.867E-29 | 2.917E-04 | 3.457E-01 |
| LongRunHighGrayLevelEmphasis glrlm MYO ED | Local Uniformity | 0.647 | 0.005 | 0.038 | -0.129 | 1.000E-100 | 1.000E+00 | 2.241E-04 | 4.300E-14 |
| MaximumProbability glcm MYO ES | Local Uniformity | 0.331 | 0.047 | 0.057 | -0.023 | 1.685E-49 | 2.584E-03 | 2.691E-10 | 1.000E+00 |
| JointEnergy glcm MYO ES | Local Uniformity | 0.382 | 0.070 | 0.068 | -0.025 | 2.728E-67 | 8.475E-09 | 3.149E-15 | 1.000E+00 |
| GrayLevelNonUniformityNormalized glszm MYO ES | Local Uniformity | 0.211 | 0.094 | 0.092 | -0.041 | 9.459E-20 | 5.636E-16 | 2.311E-27 | 1.000E+00 |
| GrayLevelNonUniformityNormalized glrlm MYO ES | Local Uniformity | 0.269 | 0.087 | 0.103 | -0.028 | 6.587E-33 | 8.592E-14 | 2.355E-35 | 1.000E+00 |
| Uniformity MYO ES | Local Uniformity | 0.256 | 0.085 | 0.106 | -0.026 | 1.739E-29 | 4.912E-13 | 6.412E-37 | 1.000E+00 |
| Elongation RV ES | Shape | 0.298 | 0.052 | -0.135 | 0.023 | 3.118E-40 | 1.990E-04 | 2.638E-60 | 1.000E+00 |
| InformalMeasureofCorrelation1 glcm MYO ED | Shape | -0.467 | -0.085 | -0.112 | 0.114 | 1.000E-100 | 5.766E-14 | 1.417E-43 | 1.024E-10 |
| Sphericity LV ES | Shape | -0.268 | -0.205 | -0.111 | 0.053 | 1.139E-34 | 4.592E-84 | 2.262E-43 | 1.803E-01 |
| Skewness MYO ED | Shape | -0.278 | 0.103 | -0.071 | 0.055 | 6.251E-34 | 6.516E-19 | 1.052E-15 | 1.982E-01 |
| Sphericity LV ED | Shape | -0.210 | -0.208 | -0.067 | 0.039 | 1.454E-20 | 6.450E-85 | 2.628E-15 | 1.000E+00 |
| GrayLevelVariance glszm MYO ES | Shape | -0.479 | -0.084 | -0.058 | 0.059 | 1.000E-100 | 2.179E-13 | 3.827E-11 | 4.680E-02 |
| Kurtosis MYO ED | Shape | 0.172 | 0.021 | -0.050 | 0.040 | 1.988E-12 | 1.000E+00 | 2.265E-07 | 1.000E+00 |
| SmallAreaEmphasis glszm MYO ES | Shape | 0.000 | -0.001 | -0.047 | 0.042 | 1.000E+00 | 1.000E+00 | 1.928E-06 | 1.000E+00 |
| SizeZoneNonUniformityNormalized glszm MYO ES | Shape | 0.004 | -0.010 | -0.046 | 0.043 | 1.000E+00 | 1.000E+00 | 4.479E-06 | 1.000E+00 |
| Elongation RV ED | Shape | 0.104 | 0.032 | -0.032 | 0.000 | 6.620E-04 | 8.190E-01 | 2.751E-02 | 1.000E+00 |
| SizeZoneNonUniformityNormalized glszm MYO ED | Shape | -0.089 | -0.062 | -0.031 | 0.017 | 1.369E-02 | 3.316E-06 | 3.180E-02 | 1.000E+00 |
| SmallAreaEmphasis glszm MYO ED | Shape | -0.099 | -0.054 | -0.030 | 0.016 | 2.074E-03 | 1.835E-04 | 5.810E-02 | 1.000E+00 |
| Flatness LV ES | Shape | -0.282 | 0.012 | -0.019 | 0.011 | 5.980E-35 | 1.000E+00 | 1.000E+00 | 1.000E+00 |
| Flatness RV ES | Shape | 0.174 | 0.093 | -0.008 | 0.057 | 5.751E-13 | 2.050E-15 | 1.000E+00 | 1.096E-01 |
| Range MYO ES | Shape | -0.400 | -0.051 | -0.008 | 0.046 | 3.346E-73 | 4.599E-04 | 1.000E+00 | 9.293E-01 |
| ClusterProminence glcm MYO ES | Shape | -0.064 | 0.010 | -0.001 | 0.011 | 1.000E+00 | 1.000E+00 | 1.000E+00 | 1.000E+00 |
| Elongation LV ED | Shape | -0.394 | 0.018 | -0.001 | -0.009 | 3.620E-69 | 1.000E+00 | 1.000E+00 | 1.000E+00 |
| Maximum MYO ES | Shape | -0.388 | -0.061 | 0.002 | 0.048 | 6.861E-69 | 2.985E-06 | 1.000E+00 | 6.066E-01 |
| Complexity ngtdm MYO ES | Shape | -0.472 | -0.021 | 0.015 | 0.046 | 1.000E-100 | 1.000E+00 | 1.000E+00 | 8.830E-01 |
| Strength ngtdm MYO ES | Shape | -0.437 | -0.115 | 0.024 | 0.042 | 1.355E-90 | 5.977E-26 | 6.458E-01 | 1.000E+00 |
| Elongation LV ES | Shape | -0.279 | 0.039 | 0.025 | 0.021 | 5.186E-34 | 8.755E-02 | 5.599E-01 | 1.000E+00 |
| ClusterShade glcm MYO ED | Shape | -0.680 | 0.036 | 0.028 | 0.000 | 1.000E-100 | 1.062E-01 | 6.624E-02 | 1.000E+00 |
| Minimum MYO ED | Shape | -0.102 | -0.025 | 0.029 | 0.037 | 9.533E-04 | 1.000E+00 | 8.877E-02 | 1.000E+00 |
| ClusterShade glcm MYO ES | Shape | -0.079 | 0.053 | 0.031 | 0.006 | 9.017E-02 | 3.045E-04 | 4.738E-02 | 1.000E+00 |
| Coarseness ngtdm MYO ES | Shape | -0.520 | -0.372 | 0.032 | 0.034 | 1.000E-100 | 1.000E-100 | 3.835E-04 | 1.000E+00 |
| Minimum MYO ES | Shape | -0.062 | -0.047 | 0.035 | 0.019 | 1.000E+00 | 3.425E-03 | 4.904E-03 | 1.000E+00 |
| ClusterProminence glcm MYO ED | Shape | -0.405 | 0.008 | 0.039 | -0.022 | 9.974E-74 | 1.000E+00 | 4.243E-04 | 1.000E+00 |
| Sphericity RV ES | Shape | -0.098 | 0.134 | 0.048 | -0.020 | 2.048E-03 | 1.646E-32 | 1.353E-06 | 1.000E+00 |
| Flatness LV ED | Shape | -0.345 | 0.018 | 0.059 | -0.073 | 7.221E-53 | 1.000E+00 | 9.567E-11 | 1.596E-03 |
| Sphericity RV ED | Shape | -0.170 | 0.142 | 0.064 | -0.014 | 3.446E-12 | 1.560E-36 | 1.708E-12 | 1.000E+00 |
| SurfaceAreatoVolumeRatio RV ED | Shape | -0.566 | -0.439 | 0.107 | -0.020 | 1.000E-100 | 1.000E-100 | 2.734E-64 | 1.000E+00 |
| Coarseness ngtdm MYO ED | Shape | -0.740 | -0.362 | 0.110 | -0.054 | 1.000E-100 | 1.000E-100 | 2.223E-69 | 2.962E-03 |
| Kurtosis MYO ES | Shape | -0.298 | 0.029 | 0.114 | 0.005 | 2.286E-39 | 1.000E+00 | 9.404E-42 | 1.000E+00 |
| SurfaceAreatoVolumeRatio RV ES | Shape | -0.679 | -0.361 | 0.119 | -0.030 | 1.000E-100 | 1.000E-100 | 2.069E-75 | 1.000E+00 |
| InformalMeasureofCorrelation1 glcm MYO ES | Shape | -0.848 | -0.008 | 0.129 | 0.073 | 1.000E-100 | 1.000E+00 | 5.202E-65 | 2.076E-04 |
| Skewness MYO ES | Shape | -0.189 | 0.164 | 0.133 | -0.001 | 1.471E-15 | 1.370E-49 | 3.112E-57 | 1.000E+00 |
| Flatness RV ED | Shape | -0.167 | 0.152 | 0.186 | 0.002 | 4.716E-12 | 4.825E-43 | 1.000E-100 | 1.000E+00 |
| SurfaceAreatoVolumeRatio LV ED | Shape | -0.458 | -0.312 | 0.189 | -0.058 | 1.000E-100 | 1.000E-100 | 1.000E-100 | 9.757E-03 |
| SurfaceAreatoVolumeRatio LV ES | Shape | -0.543 | -0.252 | 0.202 | -0.076 | 1.000E-100 | 1.000E-100 | 1.000E-100 | 1.725E-05 |
| MajorAxis RV ED | Size | 0.712 | 0.279 | -0.199 | 0.002 | 1.000E-100 | 1.000E-100 | 1.000E-100 | 1.000E+00 |
| MinorAxis RV ES | Size | 0.746 | 0.232 | -0.193 | -0.009 | 1.000E-100 | 1.000E-100 | 1.000E-100 | 1.000E+00 |
| MinorAxis RV ED | Size | 0.666 | 0.246 | -0.190 | -0.003 | 1.000E-100 | 1.000E-100 | 1.000E-100 | 1.000E+00 |
| SurfaceArea RV ED | Size | 0.812 | 0.382 | -0.172 | -0.025 | 1.000E-100 | 1.000E-100 | 1.000E-100 | 1.000E+00 |
| Max2DdiameterColumn RV ED | Size | 0.537 | 0.395 | -0.171 | 0.007 | 1.000E-100 | 1.000E-100 | 1.000E-100 | 1.000E+00 |
| SurfaceArea RV ES | Size | 0.905 | 0.338 | -0.167 | -0.031 | 1.000E-100 | 1.000E-100 | 1.000E-100 | 1.000E+00 |
| Max2DdiameterColumn LV ED | Size | 0.717 | 0.376 | -0.167 | 0.023 | 1.000E-100 | 1.000E-100 | 1.000E-100 | 1.000E+00 |
| MinorAxis LV ED | Size | 0.413 | 0.448 | -0.166 | 0.024 | 1.000E-100 | 1.000E-100 | 1.000E-100 | 1.000E+00 |
| Max2DdiameterColumn RV ES | Size | 0.561 | 0.362 | -0.166 | 0.018 | 1.000E-100 | 1.000E-100 | 1.000E-100 | 1.000E+00 |
| Volume LV ED | Size | 0.654 | 0.458 | -0.163 | -0.026 | 1.000E-100 | 1.000E-100 | 1.000E-100 | 1.000E+00 |
| Volume RV ED | Size | 0.787 | 0.428 | -0.158 | -0.046 | 1.000E-100 | 1.000E-100 | 1.000E-100 | 5.157E-03 |
| Max3Ddiameter RV ED | Size | 0.760 | 0.327 | -0.156 | -0.007 | 1.000E-100 | 1.000E-100 | 1.000E-100 | 1.000E+00 |
| Volume RV ES | Size | 0.867 | 0.357 | -0.153 | -0.058 | 1.000E-100 | 1.000E-100 | 1.000E-100 | 6.990E-05 |
| Max2DdiameterRow RV ED | Size | 0.703 | 0.258 | -0.147 | -0.026 | 1.000E-100 | 1.000E-100 | 1.192E-100 | 1.000E+00 |
| Max2DdiameterRow LV ED | Size | 0.663 | 0.351 | -0.146 | 0.022 | 1.000E-100 | 1.000E-100 | 1.000E-100 | 1.000E+00 |
| Volume LV ES | Size | 0.730 | 0.370 | -0.142 | -0.027 | 1.000E-100 | 1.000E-100 | 1.000E-100 | 1.000E+00 |
| Max2DdiameterRow LV ES | Size | 0.721 | 0.309 | -0.142 | 0.011 | 1.000E-100 | 1.000E-100 | 1.000E-100 | 1.000E+00 |
| Max2DdiameterSlice RV ED | Size | 0.629 | 0.180 | -0.140 | -0.005 | 1.000E-100 | 1.124E-73 | 1.844E-78 | 1.000E+00 |
| SurfaceArea LV ED | Size | 0.662 | 0.486 | -0.139 | -0.019 | 1.000E-100 | 1.000E-100 | 1.000E-100 | 1.000E+00 |
| Max3Ddiameter LV ED | Size | 0.655 | 0.360 | -0.137 | 0.019 | 1.000E-100 | 1.000E-100 | 1.458E-98 | 1.000E+00 |
| Max2DdiameterColumn LV ES | Size | 0.760 | 0.315 | -0.136 | 0.009 | 1.000E-100 | 1.000E-100 | 7.085E-98 | 1.000E+00 |
| LeastAxis LV ES | Size | 0.613 | 0.372 | -0.133 | 0.003 | 1.000E-100 | 1.000E-100 | 6.157E-92 | 1.000E+00 |
| Max2DdiameterRow RV ES | Size | 0.746 | 0.142 | -0.133 | -0.035 | 1.000E-100 | 3.672E-47 | 1.755E-72 | 1.000E+00 |
| MajorAxis LV ED | Size | 0.726 | 0.352 | -0.132 | 0.023 | 1.000E-100 | 1.000E-100 | 1.043E-96 | 1.000E+00 |
| Max3Ddiameter LV ES | Size | 0.710 | 0.319 | -0.121 | 0.005 | 1.000E-100 | 1.000E-100 | 4.884E-75 | 1.000E+00 |
| SurfaceArea LV ES | Size | 0.757 | 0.402 | -0.117 | -0.018 | 1.000E-100 | 1.000E-100 | 7.522E-88 | 1.000E+00 |
| Max3Ddiameter RV ES | Size | 0.773 | 0.320 | -0.113 | -0.025 | 1.000E-100 | 1.000E-100 | 1.289E-69 | 1.000E+00 |
| Max2DdiameterSlice LV ED | Size | 0.335 | 0.365 | -0.104 | 0.010 | 7.756E-65 | 1.000E-100 | 2.583E-45 | 1.000E+00 |
| LeastAxis LV ED | Size | 0.494 | 0.448 | -0.102 | -0.042 | 1.000E-100 | 1.000E-100 | 1.170E-55 | 1.910E-01 |
| GrayLevelNonUniformity glszm MYO ED | Size | -0.052 | 0.341 | -0.101 | -0.001 | 1.000E+00 | 1.000E-100 | 1.171E-36 | 1.000E+00 |
| SizeZoneNonUniformity glszm MYO ED | Size | -0.052 | 0.341 | -0.101 | -0.001 | 1.000E+00 | 1.000E-100 | 1.171E-36 | 1.000E+00 |
| MajorAxis LV ES | Size | 0.790 | 0.325 | -0.101 | -0.003 | 1.000E-100 | 1.000E-100 | 1.137E-56 | 1.000E+00 |
| LeastAxis RV ES | Size | 0.781 | 0.344 | -0.095 | 0.030 | 1.000E-100 | 1.000E-100 | 1.816E-51 | 1.000E+00 |
| MajorAxis RV ES | Size | 0.690 | 0.295 | -0.093 | -0.025 | 1.000E-100 | 1.000E-100 | 6.769E-41 | 1.000E+00 |
| MinorAxis LV ES | Size | 0.594 | 0.389 | -0.089 | 0.019 | 1.000E-100 | 1.000E-100 | 1.029E-40 | 1.000E+00 |
| Max2DdiameterSlice RV ES | Size | 0.629 | 0.205 | -0.074 | -0.025 | 1.000E-100 | 1.003E-96 | 4.421E-22 | 1.000E+00 |
| RunLengthNonUniformity glrlm MYO ES | Size | 0.205 | 0.312 | -0.053 | -0.028 | 3.516E-21 | 1.000E-100 | 8.540E-10 | 1.000E+00 |
| RunLengthNonUniformity glrlm MYO ED | Size | 0.122 | 0.400 | -0.037 | -0.010 | 2.109E-07 | 1.000E-100 | 1.468E-04 | 1.000E+00 |
| DependenceNonUniformity gldm MYO ED | Size | 0.297 | 0.385 | -0.037 | -0.060 | 1.816E-50 | 1.000E-100 | 6.759E-05 | 6.778E-03 |
| Max2DdiameterSlice LV ES | Size | 0.553 | 0.352 | -0.027 | -0.006 | 1.000E-100 | 1.000E-100 | 2.018E-02 | 1.000E+00 |
| LeastAxis RV ED | Size | 0.550 | 0.446 | -0.013 | 0.020 | 1.000E-100 | 1.000E-100 | 1.000E+00 | 1.000E+00 |
| GrayLevelNonUniformity glszm MYO ES | Size | 0.090 | 0.308 | 0.052 | -0.010 | 4.025E-03 | 1.000E-100 | 4.537E-09 | 1.000E+00 |
| SizeZoneNonUniformity glszm MYO ES | Size | 0.090 | 0.308 | 0.052 | -0.010 | 4.025E-03 | 1.000E-100 | 4.537E-09 | 1.000E+00 |

**Supplementary Table 5. Associations of vascular risk factors with radiomics features from linear regression models**

|  |  | **Beta** | | | | **Adjusted p-value** | | | |
| --- | --- | --- | --- | --- | --- | --- | --- | --- | --- |
| **Feature** | **Cluster** | **Diabetes** | **High chol.** | **Hypertension** | **Smoker** | **Diabetes** | **High chol.** | **Hypertension** | **Smoker** |
| InterquartileRange MYO ES | Global Intensity | -0.296 | -0.105 | -0.136 | -0.209 | 4.136E-25 | 3.446E-12 | 4.988E-21 | 4.599E-09 |
| RobustMeanAbsoluteDeviation MYO ES | Global Intensity | -0.296 | -0.100 | -0.136 | -0.189 | 4.765E-25 | 5.695E-11 | 6.003E-21 | 3.015E-07 |
| Percentile90 MYO ES | Global Intensity | -0.303 | -0.138 | -0.116 | -0.166 | 2.437E-28 | 3.297E-23 | 2.325E-16 | 6.928E-06 |
| RootMeanSquared MYO ES | Global Intensity | -0.274 | -0.145 | -0.104 | -0.160 | 1.378E-23 | 3.783E-26 | 3.026E-13 | 1.555E-05 |
| MeanAbsoluteDeviation MYO ES | Global Intensity | -0.281 | -0.091 | -0.152 | -0.160 | 1.224E-22 | 7.065E-09 | 1.091E-26 | 6.379E-05 |
| Mean MYO ES | Global Intensity | -0.270 | -0.146 | -0.100 | -0.160 | 6.205E-23 | 1.541E-26 | 4.371E-12 | 1.691E-05 |
| Median MYO ES | Global Intensity | -0.255 | -0.147 | -0.096 | -0.157 | 1.663E-20 | 3.923E-27 | 2.731E-11 | 2.528E-05 |
| Entropy MYO ES | Global Intensity | -0.281 | -0.104 | -0.163 | -0.153 | 8.168E-24 | 1.216E-12 | 3.050E-32 | 1.139E-04 |
| SumEntropy glcm MYO ES | Global Intensity | -0.296 | -0.102 | -0.136 | -0.143 | 1.574E-25 | 1.184E-11 | 1.588E-21 | 7.904E-04 |
| Percentile10 MYO ES | Global Intensity | -0.225 | -0.139 | -0.071 | -0.127 | 6.863E-16 | 2.942E-24 | 8.821E-06 | 4.146E-03 |
| JointEntropy glcm MYO ES | Global Intensity | -0.247 | -0.090 | -0.150 | -0.126 | 2.023E-18 | 2.242E-09 | 1.911E-27 | 6.903E-03 |
| Percentile10 MYO ED | Global Intensity | -0.195 | -0.114 | 0.027 | -0.115 | 9.570E-11 | 9.512E-15 | 1.000E+00 | 4.434E-02 |
| Mean MYO ED | Global Intensity | -0.165 | -0.086 | -0.036 | -0.099 | 1.667E-08 | 3.438E-09 | 1.000E+00 | 1.552E-01 |
| Percentile90 MYO ED | Global Intensity | -0.145 | -0.060 | -0.086 | -0.098 | 4.895E-07 | 1.806E-04 | 3.374E-10 | 8.771E-02 |
| RootMeanSquared MYO ED | Global Intensity | -0.160 | -0.079 | -0.047 | -0.097 | 4.594E-08 | 9.278E-08 | 4.338E-02 | 1.716E-01 |
| Maximum MYO ED | Global Intensity | -0.148 | -0.047 | -0.062 | -0.085 | 2.268E-06 | 6.174E-02 | 4.390E-04 | 8.913E-01 |
| Median MYO ED | Global Intensity | -0.156 | -0.091 | -0.018 | -0.084 | 2.646E-07 | 3.761E-10 | 1.000E+00 | 9.777E-01 |
| SmallAreaHighGrayLevelEmphasis glszm MYO ES | Global Intensity | -0.179 | -0.103 | -0.148 | -0.083 | 1.073E-08 | 1.221E-11 | 7.327E-25 | 1.000E+00 |
| Variance MYO ES | Global Intensity | -0.209 | -0.057 | -0.129 | -0.082 | 3.957E-12 | 6.485E-03 | 7.682E-19 | 1.000E+00 |
| GrayLevelVariance gldm MYO ES | Global Intensity | -0.223 | -0.065 | -0.130 | -0.080 | 5.246E-14 | 4.480E-04 | 2.212E-19 | 1.000E+00 |
| GrayLevelVariance glrlm MYO ES | Global Intensity | -0.205 | -0.063 | -0.128 | -0.079 | 1.072E-11 | 8.213E-04 | 1.682E-18 | 1.000E+00 |
| Energy MYO ES | Global Intensity | -0.357 | -0.158 | 0.083 | -0.073 | 4.173E-40 | 4.202E-31 | 4.877E-08 | 1.000E+00 |
| ClusterTendency glcm MYO ES | Global Intensity | -0.257 | -0.063 | -0.102 | -0.072 | 2.532E-18 | 1.101E-03 | 3.571E-11 | 1.000E+00 |
| SumofSquares glcm MYO ES | Global Intensity | -0.229 | -0.059 | -0.115 | -0.070 | 1.104E-14 | 3.674E-03 | 1.337E-14 | 1.000E+00 |
| SmallDependenceHighGrayLevelEmphasis gldm MYO ES | Global Intensity | -0.103 | -0.068 | -0.193 | -0.067 | 1.970E-02 | 4.327E-05 | 4.514E-47 | 1.000E+00 |
| Complexity ngtdm MYO ED | Global Intensity | -0.099 | -0.031 | -0.087 | -0.065 | 2.206E-02 | 1.000E+00 | 2.443E-09 | 1.000E+00 |
| Range MYO ED | Global Intensity | -0.126 | -0.044 | -0.068 | -0.062 | 2.213E-04 | 1.729E-01 | 2.586E-05 | 1.000E+00 |
| SmallDependenceHighGrayLevelEmphasis gldm MYO ED | Global Intensity | -0.060 | -0.040 | -0.151 | -0.060 | 1.000E+00 | 1.465E-01 | 2.152E-36 | 1.000E+00 |
| Strength ngtdm MYO ED | Global Intensity | -0.021 | -0.001 | -0.070 | -0.056 | 1.000E+00 | 1.000E+00 | 7.232E-06 | 1.000E+00 |
| HighGrayLevelZoneEmphasis glszm MYO ES | Global Intensity | -0.198 | -0.096 | -0.144 | -0.055 | 9.023E-11 | 6.046E-10 | 1.832E-23 | 1.000E+00 |
| TotalEnergy MYO ES | Global Intensity | -0.337 | -0.146 | 0.109 | -0.052 | 4.006E-37 | 1.631E-27 | 5.334E-15 | 1.000E+00 |
| ZoneEntropy glszm MYO ES | Global Intensity | -0.234 | -0.086 | -0.106 | -0.050 | 5.528E-15 | 1.146E-07 | 4.452E-12 | 1.000E+00 |
| SmallAreaHighGrayLevelEmphasis glszm MYO ED | Global Intensity | -0.115 | -0.066 | -0.090 | -0.031 | 1.509E-03 | 5.579E-05 | 5.047E-10 | 1.000E+00 |
| HighGrayLevelZoneEmphasis glszm MYO ED | Global Intensity | -0.105 | -0.060 | -0.086 | -0.030 | 1.020E-02 | 8.211E-04 | 3.913E-09 | 1.000E+00 |
| Energy MYO ED | Global Intensity | -0.317 | -0.123 | 0.116 | -0.027 | 6.972E-30 | 1.240E-17 | 9.140E-16 | 1.000E+00 |
| ShortRunHighGrayLevelEmphasis glrlm MYO ES | Global Intensity | -0.123 | -0.075 | -0.143 | -0.013 | 1.327E-03 | 8.897E-06 | 2.090E-23 | 1.000E+00 |
| Autocorrelation glcm MYO ES | Global Intensity | -0.109 | -0.075 | -0.103 | -0.008 | 1.639E-02 | 1.233E-05 | 1.274E-11 | 1.000E+00 |
| SumAverage glcm MYO ES | Global Intensity | -0.107 | -0.074 | -0.102 | -0.007 | 2.111E-02 | 1.339E-05 | 2.122E-11 | 1.000E+00 |
| JointAverage glcm MYO ES | Global Intensity | -0.107 | -0.074 | -0.102 | -0.007 | 2.111E-02 | 1.339E-05 | 2.122E-11 | 1.000E+00 |
| HighGrayLevelEmphasis gldm MYO ES | Global Intensity | -0.116 | -0.076 | -0.108 | -0.007 | 5.354E-03 | 8.425E-06 | 9.410E-13 | 1.000E+00 |
| HighGrayLevelRunEmphasis glrlm MYO ES | Global Intensity | -0.125 | -0.077 | -0.109 | -0.006 | 1.183E-03 | 6.114E-06 | 4.289E-13 | 1.000E+00 |
| TotalEnergy MYO ED | Global Intensity | -0.284 | -0.107 | 0.147 | -0.001 | 4.013E-25 | 8.860E-14 | 5.689E-27 | 1.000E+00 |
| ShortRunHighGrayLevelEmphasis glrlm MYO ED | Global Intensity | -0.034 | -0.041 | -0.080 | 0.018 | 1.000E+00 | 3.204E-01 | 1.240E-07 | 1.000E+00 |
| RunEntropy glrlm MYO ED | Global Intensity | -0.128 | -0.028 | 0.076 | 0.026 | 8.458E-04 | 1.000E+00 | 8.900E-06 | 1.000E+00 |
| HighGrayLevelRunEmphasis glrlm MYO ED | Global Intensity | -0.027 | -0.045 | -0.047 | 0.043 | 1.000E+00 | 1.749E-01 | 1.056E-01 | 1.000E+00 |
| HighGrayLevelEmphasis gldm MYO ED | Global Intensity | -0.015 | -0.043 | -0.042 | 0.054 | 1.000E+00 | 2.937E-01 | 3.350E-01 | 1.000E+00 |
| InformalMeasureofCorrelation2 glcm MYO ED | Global Intensity | -0.067 | -0.020 | 0.047 | 0.057 | 1.000E+00 | 1.000E+00 | 1.661E-01 | 1.000E+00 |
| DependenceEntropy gldm MYO ED | Global Intensity | -0.127 | -0.016 | 0.125 | 0.076 | 8.516E-04 | 1.000E+00 | 2.323E-17 | 1.000E+00 |
| JointAverage glcm MYO ED | Global Intensity | 0.011 | -0.045 | -0.025 | 0.078 | 1.000E+00 | 2.408E-01 | 1.000E+00 | 1.000E+00 |
| SumAverage glcm MYO ED | Global Intensity | 0.011 | -0.045 | -0.025 | 0.078 | 1.000E+00 | 2.408E-01 | 1.000E+00 | 1.000E+00 |
| Autocorrelation glcm MYO ED | Global Intensity | 0.005 | -0.045 | -0.025 | 0.078 | 1.000E+00 | 2.232E-01 | 1.000E+00 | 1.000E+00 |
| Correlation glcm MYO ED | Global Intensity | -0.055 | -0.015 | 0.101 | 0.086 | 1.000E+00 | 1.000E+00 | 2.439E-11 | 1.000E+00 |
| ZonePercentage glszm MYO ED | Global Variance | -0.033 | -0.012 | -0.180 | -0.108 | 1.000E+00 | 1.000E+00 | 7.978E-61 | 2.229E-03 |
| DifferenceVariance glcm MYO ED | Global Variance | -0.089 | -0.024 | -0.151 | -0.104 | 3.106E-02 | 1.000E+00 | 8.493E-36 | 2.255E-02 |
| SmallDependenceEmphasis gldm MYO ED | Global Variance | -0.033 | -0.012 | -0.189 | -0.104 | 1.000E+00 | 1.000E+00 | 3.619E-69 | 3.751E-03 |
| DifferenceEntropy glcm MYO ED | Global Variance | -0.073 | -0.019 | -0.168 | -0.098 | 1.897E-01 | 1.000E+00 | 1.601E-50 | 2.227E-02 |
| Contrast glcm MYO ED | Global Variance | -0.069 | -0.020 | -0.155 | -0.084 | 5.837E-01 | 1.000E+00 | 5.107E-39 | 3.171E-01 |
| SmallDependenceEmphasis gldm MYO ES | Global Variance | 0.001 | -0.021 | -0.214 | -0.081 | 1.000E+00 | 1.000E+00 | 5.872E-66 | 9.165E-01 |
| ZonePercentage glszm MYO ES | Global Variance | 0.007 | -0.012 | -0.207 | -0.080 | 1.000E+00 | 1.000E+00 | 1.445E-59 | 1.000E+00 |
| ShortRunEmphasis glrlm MYO ED | Global Variance | -0.015 | 0.005 | -0.187 | -0.080 | 1.000E+00 | 1.000E+00 | 2.374E-64 | 2.982E-01 |
| DifferenceEntropy glcm MYO ES | Global Variance | -0.113 | -0.052 | -0.152 | -0.076 | 1.415E-03 | 7.812E-03 | 3.122E-31 | 1.000E+00 |
| RunLengthNonUniformityNormalized glrlm MYO ED | Global Variance | -0.008 | 0.005 | -0.188 | -0.075 | 1.000E+00 | 1.000E+00 | 1.279E-63 | 6.370E-01 |
| RunPercentage glrlm MYO ES | Global Variance | -0.049 | -0.032 | -0.191 | -0.071 | 1.000E+00 | 1.000E+00 | 3.087E-52 | 1.000E+00 |
| DifferenceAverage glcm MYO ED | Global Variance | -0.050 | -0.016 | -0.158 | -0.071 | 1.000E+00 | 1.000E+00 | 8.811E-44 | 1.000E+00 |
| Contrast ngtdm MYO ES | Global Variance | -0.095 | -0.011 | -0.073 | -0.068 | 1.066E-01 | 1.000E+00 | 1.786E-05 | 1.000E+00 |
| RunPercentage glrlm MYO ED | Global Variance | -0.003 | 0.004 | -0.177 | -0.066 | 1.000E+00 | 1.000E+00 | 1.337E-56 | 1.000E+00 |
| Contrast ngtdm MYO ED | Global Variance | -0.015 | 0.020 | -0.144 | -0.065 | 1.000E+00 | 1.000E+00 | 2.525E-30 | 1.000E+00 |
| DependenceNonUniformityNormalized gldm MYO ES | Global Variance | -0.060 | -0.020 | -0.113 | -0.058 | 1.000E+00 | 1.000E+00 | 4.327E-15 | 1.000E+00 |
| DifferenceVariance glcm MYO ES | Global Variance | -0.108 | -0.031 | -0.120 | -0.058 | 8.044E-03 | 1.000E+00 | 1.285E-17 | 1.000E+00 |
| MeanAbsoluteDeviation MYO ED | Global Variance | -0.077 | -0.003 | -0.128 | -0.058 | 2.267E-01 | 1.000E+00 | 8.830E-26 | 1.000E+00 |
| DifferenceAverage glcm MYO ES | Global Variance | -0.108 | -0.048 | -0.136 | -0.057 | 4.985E-03 | 3.345E-02 | 2.429E-24 | 1.000E+00 |
| InverseVariance glcm MYO ES | Global Variance | -0.109 | -0.053 | -0.120 | -0.053 | 4.462E-03 | 8.907E-03 | 1.223E-18 | 1.000E+00 |
| GrayLevelVariance glrlm MYO ED | Global Variance | -0.078 | 0.000 | -0.115 | -0.052 | 3.527E-01 | 1.000E+00 | 1.734E-18 | 1.000E+00 |
| Entropy MYO ED | Global Variance | -0.069 | -0.012 | -0.130 | -0.051 | 5.397E-01 | 1.000E+00 | 7.498E-28 | 1.000E+00 |
| ShortRunEmphasis glrlm MYO ES | Global Variance | -0.018 | -0.022 | -0.175 | -0.050 | 1.000E+00 | 1.000E+00 | 1.511E-42 | 1.000E+00 |
| Contrast glcm MYO ES | Global Variance | -0.103 | -0.035 | -0.120 | -0.050 | 1.789E-02 | 1.000E+00 | 7.666E-18 | 1.000E+00 |
| Variance MYO ED | Global Variance | -0.072 | 0.002 | -0.122 | -0.049 | 7.270E-01 | 1.000E+00 | 4.535E-21 | 1.000E+00 |
| GrayLevelVariance gldm MYO ED | Global Variance | -0.074 | 0.000 | -0.121 | -0.049 | 5.521E-01 | 1.000E+00 | 5.873E-21 | 1.000E+00 |
| JointEntropy glcm MYO ED | Global Variance | -0.060 | -0.016 | -0.130 | -0.048 | 1.000E+00 | 1.000E+00 | 1.672E-27 | 1.000E+00 |
| RunLengthNonUniformityNormalized glrlm MYO ES | Global Variance | -0.017 | -0.022 | -0.174 | -0.046 | 1.000E+00 | 1.000E+00 | 2.832E-41 | 1.000E+00 |
| SumofSquares glcm MYO ED | Global Variance | -0.074 | -0.012 | -0.124 | -0.046 | 5.231E-01 | 1.000E+00 | 1.926E-22 | 1.000E+00 |
| DependenceNonUniformityNormalized gldm MYO ED | Global Variance | 0.026 | -0.001 | -0.162 | -0.039 | 1.000E+00 | 1.000E+00 | 6.479E-43 | 1.000E+00 |
| RobustMeanAbsoluteDeviation MYO ED | Global Variance | -0.071 | -0.001 | -0.102 | -0.038 | 6.816E-01 | 1.000E+00 | 2.350E-15 | 1.000E+00 |
| SumEntropy glcm MYO ED | Global Variance | -0.067 | -0.018 | -0.110 | -0.037 | 9.525E-01 | 1.000E+00 | 2.184E-18 | 1.000E+00 |
| ZoneEntropy glszm MYO ED | Global Variance | -0.078 | -0.021 | -0.080 | -0.026 | 7.330E-01 | 1.000E+00 | 2.554E-07 | 1.000E+00 |
| InterquartileRange MYO ED | Global Variance | -0.066 | 0.002 | -0.089 | -0.025 | 1.000E+00 | 1.000E+00 | 4.178E-11 | 1.000E+00 |
| ClusterTendency glcm MYO ED | Global Variance | -0.073 | -0.008 | -0.105 | -0.025 | 7.510E-01 | 1.000E+00 | 8.814E-15 | 1.000E+00 |
| InverseVariance glcm MYO ED | Global Variance | 0.002 | 0.001 | -0.094 | -0.009 | 1.000E+00 | 1.000E+00 | 1.094E-12 | 1.000E+00 |
| GrayLevelVariance glszm MYO ED | Global Variance | -0.053 | 0.001 | -0.066 | 0.012 | 1.000E+00 | 1.000E+00 | 5.966E-05 | 1.000E+00 |
| SmallDependenceLowGrayLevelEmphasis gldm MYO ED | Local Dimness | -0.005 | 0.055 | -0.066 | -0.092 | 1.000E+00 | 1.369E-02 | 2.394E-04 | 7.023E-01 |
| ShortRunLowGrayLevelEmphasis glrlm MYO ED | Local Dimness | -0.004 | 0.053 | -0.010 | -0.071 | 1.000E+00 | 3.221E-02 | 1.000E+00 | 1.000E+00 |
| LowGrayLevelEmphasis gldm MYO ED | Local Dimness | -0.014 | 0.046 | 0.008 | -0.071 | 1.000E+00 | 1.946E-01 | 1.000E+00 | 1.000E+00 |
| LowGrayLevelRunEmphasis glrlm MYO ED | Local Dimness | -0.006 | 0.049 | 0.013 | -0.061 | 1.000E+00 | 9.730E-02 | 1.000E+00 | 1.000E+00 |
| SmallDependenceLowGrayLevelEmphasis gldm MYO ES | Local Dimness | 0.144 | 0.078 | -0.034 | -0.035 | 2.821E-05 | 2.927E-06 | 1.000E+00 | 1.000E+00 |
| ShortRunLowGrayLevelEmphasis glrlm MYO ES | Local Dimness | 0.122 | 0.065 | 0.048 | -0.016 | 2.399E-03 | 5.755E-04 | 1.193E-01 | 1.000E+00 |
| LargeDependenceLowGrayLevelEmphasis gldm MYO ED | Local Dimness | -0.021 | 0.022 | 0.094 | -0.010 | 1.000E+00 | 1.000E+00 | 3.892E-11 | 1.000E+00 |
| LowGrayLevelEmphasis gldm MYO ES | Local Dimness | 0.094 | 0.060 | 0.076 | -0.006 | 1.370E-01 | 3.711E-03 | 8.286E-06 | 1.000E+00 |
| LowGrayLevelRunEmphasis glrlm MYO ES | Local Dimness | 0.113 | 0.064 | 0.077 | -0.004 | 9.148E-03 | 7.737E-04 | 5.933E-06 | 1.000E+00 |
| LowGrayLevelZoneEmphasis glszm MYO ED | Local Dimness | 0.030 | 0.057 | 0.062 | -0.003 | 1.000E+00 | 7.559E-03 | 1.281E-03 | 1.000E+00 |
| SmallAreaLowGrayLevelEmphasis glszm MYO ED | Local Dimness | 0.023 | 0.059 | 0.070 | 0.002 | 1.000E+00 | 3.045E-03 | 6.244E-05 | 1.000E+00 |
| LongRunLowGrayLevelEmphasis glrlm MYO ED | Local Dimness | -0.010 | 0.028 | 0.090 | 0.004 | 1.000E+00 | 1.000E+00 | 6.168E-10 | 1.000E+00 |
| SmallAreaLowGrayLevelEmphasis glszm MYO ES | Local Dimness | 0.154 | 0.082 | 0.094 | 0.005 | 6.235E-06 | 7.893E-07 | 3.245E-09 | 1.000E+00 |
| LowGrayLevelZoneEmphasis glszm MYO ES | Local Dimness | 0.157 | 0.088 | 0.093 | 0.019 | 3.292E-06 | 5.800E-08 | 4.379E-09 | 1.000E+00 |
| Busyness ngtdm MYO ED | Local Dimness | -0.071 | -0.001 | 0.096 | 0.041 | 1.000E+00 | 1.000E+00 | 2.623E-12 | 1.000E+00 |
| LargeDependenceLowGrayLevelEmphasis gldm MYO ES | Local Dimness | 0.087 | 0.057 | 0.139 | 0.044 | 2.185E-01 | 2.987E-03 | 9.815E-24 | 1.000E+00 |
| Busyness ngtdm MYO ES | Local Dimness | 0.022 | 0.018 | 0.176 | 0.052 | 1.000E+00 | 1.000E+00 | 4.271E-41 | 1.000E+00 |
| LongRunLowGrayLevelEmphasis glrlm MYO ES | Local Dimness | 0.083 | 0.056 | 0.153 | 0.058 | 3.558E-01 | 4.983E-03 | 2.219E-29 | 1.000E+00 |
| LargeAreaLowGrayLevelEmphasis glszm MYO ED | Local Dimness | -0.006 | -0.002 | 0.103 | 0.072 | 1.000E+00 | 1.000E+00 | 9.209E-14 | 1.000E+00 |
| LargeAreaLowGrayLevelEmphasis glszm MYO ES | Local Dimness | 0.062 | 0.009 | 0.183 | 0.095 | 1.000E+00 | 1.000E+00 | 2.178E-44 | 2.442E-01 |
| InformalMeasureofCorrelation2 glcm MYO ES | Local Uniformity | -0.343 | -0.093 | -0.030 | -0.145 | 6.383E-36 | 6.829E-10 | 1.000E+00 | 3.801E-04 |
| DependenceEntropy gldm MYO ES | Local Uniformity | -0.348 | -0.111 | -0.044 | -0.133 | 1.462E-34 | 2.013E-13 | 3.550E-01 | 4.909E-03 |
| Correlation glcm MYO ES | Local Uniformity | -0.308 | -0.079 | 0.004 | -0.110 | 1.453E-30 | 2.330E-07 | 1.000E+00 | 4.216E-02 |
| RunEntropy glrlm MYO ES | Local Uniformity | -0.256 | -0.079 | 0.066 | -0.070 | 8.889E-21 | 3.380E-07 | 7.800E-05 | 1.000E+00 |
| InverseDifferenceMomentNormalized glcm MYO ES | Local Uniformity | -0.077 | -0.048 | 0.004 | -0.008 | 1.000E+00 | 1.150E-01 | 1.000E+00 | 1.000E+00 |
| GrayLevelNonUniformityNormalized glszm MYO ED | Local Uniformity | 0.051 | 0.010 | 0.074 | 0.010 | 1.000E+00 | 1.000E+00 | 9.074E-07 | 1.000E+00 |
| MaximumProbability glcm MYO ED | Local Uniformity | 0.064 | 0.026 | 0.074 | 0.017 | 1.000E+00 | 1.000E+00 | 3.607E-07 | 1.000E+00 |
| InverseDifferenceNormalized glcm MYO ES | Local Uniformity | -0.001 | -0.008 | 0.066 | 0.019 | 1.000E+00 | 1.000E+00 | 1.250E-04 | 1.000E+00 |
| JointEnergy glcm MYO ED | Local Uniformity | 0.053 | 0.013 | 0.086 | 0.027 | 1.000E+00 | 1.000E+00 | 2.757E-10 | 1.000E+00 |
| LargeDependenceHighGrayLevelEmphasis gldm MYO ES | Local Uniformity | -0.063 | -0.052 | 0.032 | 0.029 | 1.000E+00 | 3.960E-02 | 1.000E+00 | 1.000E+00 |
| DependenceVariance gldm MYO ED | Local Uniformity | -0.007 | 0.008 | 0.143 | 0.035 | 1.000E+00 | 1.000E+00 | 3.234E-32 | 1.000E+00 |
| Uniformity MYO ED | Local Uniformity | 0.069 | 0.015 | 0.105 | 0.039 | 7.120E-01 | 1.000E+00 | 6.587E-17 | 1.000E+00 |
| DependenceNonUniformity gldm MYO ES | Local Uniformity | -0.224 | -0.072 | 0.186 | 0.053 | 6.914E-26 | 2.582E-10 | 1.327E-72 | 1.000E+00 |
| InverseDifferenceNormalized glcm MYO ED | Local Uniformity | -0.039 | -0.023 | 0.138 | 0.054 | 1.000E+00 | 1.000E+00 | 2.709E-26 | 1.000E+00 |
| GrayLevelNonUniformityNormalized glrlm MYO ED | Local Uniformity | 0.073 | 0.009 | 0.118 | 0.054 | 3.797E-01 | 1.000E+00 | 5.968E-22 | 1.000E+00 |
| GrayLevelNonUniformity gldm MYO ED | Local Uniformity | -0.101 | -0.037 | 0.166 | 0.055 | 2.206E-05 | 1.934E-02 | 3.526E-66 | 1.000E+00 |
| LongRunHighGrayLevelEmphasis glrlm MYO ES | Local Uniformity | -0.056 | -0.038 | 0.073 | 0.056 | 1.000E+00 | 9.590E-01 | 9.557E-06 | 1.000E+00 |
| InverseDifference glcm MYO ED | Local Uniformity | 0.035 | 0.011 | 0.149 | 0.056 | 1.000E+00 | 1.000E+00 | 2.381E-38 | 1.000E+00 |
| InverseDifference glcm MYO ES | Local Uniformity | 0.108 | 0.052 | 0.135 | 0.057 | 4.264E-03 | 1.055E-02 | 3.340E-24 | 1.000E+00 |
| InverseDifferenceMoment glcm MYO ES | Local Uniformity | 0.107 | 0.051 | 0.137 | 0.057 | 5.195E-03 | 1.229E-02 | 7.878E-25 | 1.000E+00 |
| LargeDependenceEmphasis gldm MYO ED | Local Uniformity | 0.003 | -0.004 | 0.168 | 0.060 | 1.000E+00 | 1.000E+00 | 4.571E-49 | 1.000E+00 |
| InverseDifferenceMoment glcm MYO ED | Local Uniformity | 0.038 | 0.012 | 0.153 | 0.060 | 1.000E+00 | 1.000E+00 | 4.941E-41 | 1.000E+00 |
| GrayLevelNonUniformity glrlm MYO ED | Local Uniformity | -0.144 | -0.049 | 0.167 | 0.061 | 3.486E-13 | 1.608E-05 | 1.843E-73 | 7.489E-01 |
| InverseDifferenceMomentNormalized glcm MYO ED | Local Uniformity | -0.047 | -0.029 | 0.118 | 0.064 | 1.000E+00 | 1.000E+00 | 5.683E-17 | 1.000E+00 |
| RunVariance glrlm MYO ED | Local Uniformity | 0.006 | -0.001 | 0.173 | 0.075 | 1.000E+00 | 1.000E+00 | 4.545E-52 | 7.707E-01 |
| LongRunEmphasis glrlm MYO ED | Local Uniformity | 0.013 | -0.002 | 0.177 | 0.080 | 1.000E+00 | 1.000E+00 | 1.356E-54 | 3.642E-01 |
| LargeDependenceEmphasis gldm MYO ES | Local Uniformity | 0.067 | 0.036 | 0.191 | 0.080 | 1.000E+00 | 8.631E-01 | 6.825E-52 | 9.903E-01 |
| ZoneVariance glszm MYO ED | Local Uniformity | -0.029 | -0.025 | 0.157 | 0.081 | 1.000E+00 | 1.000E+00 | 2.442E-44 | 2.746E-01 |
| LargeAreaEmphasis glszm MYO ED | Local Uniformity | -0.027 | -0.025 | 0.156 | 0.082 | 1.000E+00 | 1.000E+00 | 5.620E-44 | 2.396E-01 |
| DependenceVariance gldm MYO ES | Local Uniformity | 0.121 | 0.045 | 0.139 | 0.085 | 7.552E-04 | 1.320E-01 | 3.070E-24 | 1.000E+00 |
| LongRunEmphasis glrlm MYO ES | Local Uniformity | 0.060 | 0.040 | 0.204 | 0.094 | 1.000E+00 | 2.479E-01 | 4.905E-61 | 1.591E-01 |
| RunVariance glrlm MYO ES | Local Uniformity | 0.065 | 0.042 | 0.217 | 0.097 | 1.000E+00 | 9.344E-02 | 7.332E-71 | 8.627E-02 |
| LargeAreaHighGrayLevelEmphasis glszm MYO ES | Local Uniformity | -0.043 | -0.035 | 0.189 | 0.101 | 1.000E+00 | 8.653E-01 | 9.208E-53 | 5.674E-02 |
| LargeAreaHighGrayLevelEmphasis glszm MYO ED | Local Uniformity | -0.035 | -0.041 | 0.164 | 0.105 | 1.000E+00 | 5.827E-02 | 2.646E-45 | 1.061E-02 |
| MaximumProbability glcm MYO ES | Local Uniformity | 0.250 | 0.084 | 0.097 | 0.108 | 4.408E-18 | 1.616E-07 | 1.429E-10 | 1.150E-01 |
| LargeDependenceHighGrayLevelEmphasis gldm MYO ED | Local Uniformity | 0.012 | -0.036 | 0.149 | 0.119 | 1.000E+00 | 1.000E+00 | 1.075E-28 | 1.122E-02 |
| LargeAreaEmphasis glszm MYO ES | Local Uniformity | 0.035 | -0.002 | 0.242 | 0.126 | 1.000E+00 | 1.000E+00 | 3.836E-96 | 3.400E-04 |
| JointEnergy glcm MYO ES | Local Uniformity | 0.263 | 0.093 | 0.116 | 0.126 | 2.255E-20 | 1.333E-09 | 8.552E-16 | 8.556E-03 |
| ZoneVariance glszm MYO ES | Local Uniformity | 0.035 | -0.002 | 0.242 | 0.127 | 1.000E+00 | 1.000E+00 | 1.385E-97 | 2.143E-04 |
| LongRunHighGrayLevelEmphasis glrlm MYO ED | Local Uniformity | 0.008 | -0.041 | 0.133 | 0.128 | 1.000E+00 | 4.738E-01 | 2.935E-21 | 5.385E-03 |
| GrayLevelNonUniformity glrlm MYO ES | Local Uniformity | -0.045 | -0.012 | 0.228 | 0.142 | 1.000E+00 | 1.000E+00 | 1.422E-116 | 4.751E-08 |
| GrayLevelNonUniformity gldm MYO ES | Local Uniformity | 0.038 | 0.013 | 0.243 | 0.146 | 1.000E+00 | 1.000E+00 | 1.714E-123 | 6.136E-08 |
| GrayLevelNonUniformityNormalized glszm MYO ES | Local Uniformity | 0.286 | 0.119 | 0.133 | 0.146 | 6.947E-24 | 3.399E-16 | 2.761E-20 | 4.924E-04 |
| Uniformity MYO ES | Local Uniformity | 0.309 | 0.108 | 0.132 | 0.157 | 3.340E-28 | 2.461E-13 | 3.116E-20 | 6.801E-05 |
| GrayLevelNonUniformityNormalized glrlm MYO ES | Local Uniformity | 0.282 | 0.107 | 0.144 | 0.160 | 2.030E-23 | 5.331E-13 | 3.484E-24 | 4.034E-05 |
| Flatness RV ES | Shape | -0.114 | -0.010 | -0.027 | -0.155 | 7.007E-03 | 1.000E+00 | 1.000E+00 | 1.659E-04 |
| Elongation RV ES | Shape | -0.146 | -0.021 | -0.021 | -0.152 | 1.825E-05 | 1.000E+00 | 1.000E+00 | 2.095E-04 |
| Minimum MYO ED | Shape | -0.165 | -0.036 | 0.013 | -0.151 | 5.449E-07 | 1.000E+00 | 1.000E+00 | 4.190E-04 |
| Elongation RV ED | Shape | -0.132 | -0.004 | 0.017 | -0.136 | 4.888E-04 | 1.000E+00 | 1.000E+00 | 4.269E-03 |
| Flatness RV ED | Shape | -0.005 | 0.073 | 0.102 | -0.135 | 1.000E+00 | 2.033E-05 | 1.229E-11 | 3.032E-03 |
| InformalMeasureofCorrelation1 glcm MYO ED | Shape | 0.027 | -0.002 | -0.153 | -0.134 | 1.000E+00 | 1.000E+00 | 5.640E-28 | 2.456E-03 |
| Minimum MYO ES | Shape | -0.178 | -0.057 | 0.022 | -0.129 | 2.658E-08 | 9.311E-03 | 1.000E+00 | 9.919E-03 |
| Maximum MYO ES | Shape | -0.207 | -0.108 | -0.094 | -0.115 | 3.407E-12 | 3.585E-13 | 7.645E-10 | 4.232E-02 |
| Sphericity RV ED | Shape | 0.068 | 0.120 | 0.043 | -0.112 | 1.000E+00 | 1.058E-15 | 3.952E-01 | 8.997E-02 |
| Sphericity LV ED | Shape | -0.101 | -0.114 | -0.144 | -0.107 | 3.410E-02 | 2.380E-15 | 6.990E-25 | 9.104E-02 |
| Skewness MYO ED | Shape | -0.074 | 0.002 | -0.015 | -0.102 | 1.000E+00 | 1.000E+00 | 1.000E+00 | 2.819E-01 |
| Sphericity RV ES | Shape | -0.004 | 0.060 | -0.029 | -0.102 | 1.000E+00 | 3.308E-03 | 1.000E+00 | 2.838E-01 |
| GrayLevelVariance glszm MYO ES | Shape | -0.143 | -0.083 | -0.127 | -0.089 | 1.648E-05 | 9.251E-08 | 1.876E-19 | 7.557E-01 |
| Range MYO ES | Shape | -0.169 | -0.099 | -0.109 | -0.085 | 8.289E-08 | 5.136E-11 | 1.612E-13 | 1.000E+00 |
| SmallAreaEmphasis glszm MYO ES | Shape | -0.004 | -0.026 | -0.015 | -0.084 | 1.000E+00 | 1.000E+00 | 1.000E+00 | 1.000E+00 |
| Strength ngtdm MYO ES | Shape | -0.049 | -0.036 | -0.108 | -0.077 | 1.000E+00 | 1.000E+00 | 1.201E-13 | 1.000E+00 |
| SizeZoneNonUniformityNormalized glszm MYO ES | Shape | 0.004 | -0.026 | -0.012 | -0.076 | 1.000E+00 | 1.000E+00 | 1.000E+00 | 1.000E+00 |
| Complexity ngtdm MYO ES | Shape | -0.142 | -0.065 | -0.094 | -0.058 | 3.456E-05 | 4.217E-04 | 7.712E-10 | 1.000E+00 |
| Coarseness ngtdm MYO ES | Shape | 0.147 | 0.067 | -0.169 | -0.055 | 1.475E-07 | 6.199E-06 | 3.188E-43 | 1.000E+00 |
| Flatness LV ED | Shape | -0.048 | -0.058 | -0.043 | -0.048 | 1.000E+00 | 5.753E-03 | 3.501E-01 | 1.000E+00 |
| Elongation LV ED | Shape | 0.009 | -0.046 | -0.054 | -0.039 | 1.000E+00 | 1.854E-01 | 1.940E-02 | 1.000E+00 |
| Sphericity LV ES | Shape | -0.042 | -0.115 | -0.197 | -0.038 | 1.000E+00 | 4.123E-16 | 1.133E-48 | 1.000E+00 |
| ClusterShade glcm MYO ED | Shape | -0.069 | 0.022 | -0.063 | -0.025 | 1.000E+00 | 1.000E+00 | 6.051E-04 | 1.000E+00 |
| Kurtosis MYO ED | Shape | -0.047 | -0.002 | 0.047 | -0.022 | 1.000E+00 | 1.000E+00 | 1.584E-01 | 1.000E+00 |
| Coarseness ngtdm MYO ED | Shape | 0.224 | 0.083 | -0.149 | -0.019 | 7.492E-24 | 1.246E-12 | 2.326E-42 | 1.000E+00 |
| Flatness LV ES | Shape | -0.014 | -0.072 | -0.169 | -0.018 | 1.000E+00 | 3.737E-05 | 3.564E-32 | 1.000E+00 |
| Skewness MYO ES | Shape | -0.008 | 0.019 | 0.008 | -0.016 | 1.000E+00 | 1.000E+00 | 1.000E+00 | 1.000E+00 |
| Elongation LV ES | Shape | 0.008 | -0.032 | -0.103 | -0.007 | 1.000E+00 | 1.000E+00 | 1.897E-11 | 1.000E+00 |
| SmallAreaEmphasis glszm MYO ED | Shape | -0.076 | -0.025 | 0.007 | 0.010 | 1.000E+00 | 1.000E+00 | 1.000E+00 | 1.000E+00 |
| SizeZoneNonUniformityNormalized glszm MYO ED | Shape | -0.071 | -0.024 | 0.009 | 0.012 | 1.000E+00 | 1.000E+00 | 1.000E+00 | 1.000E+00 |
| ClusterProminence glcm MYO ED | Shape | -0.044 | 0.026 | -0.034 | 0.013 | 1.000E+00 | 1.000E+00 | 1.000E+00 | 1.000E+00 |
| SurfaceAreatoVolumeRatio LV ES | Shape | 0.213 | 0.171 | 0.140 | 0.041 | 2.154E-16 | 1.031E-42 | 6.725E-29 | 1.000E+00 |
| Kurtosis MYO ES | Shape | 0.074 | 0.006 | -0.001 | 0.042 | 1.000E+00 | 1.000E+00 | 1.000E+00 | 1.000E+00 |
| ClusterProminence glcm MYO ES | Shape | -0.051 | 0.017 | -0.005 | 0.059 | 1.000E+00 | 1.000E+00 | 1.000E+00 | 1.000E+00 |
| ClusterShade glcm MYO ES | Shape | -0.061 | 0.022 | 0.001 | 0.064 | 1.000E+00 | 1.000E+00 | 1.000E+00 | 1.000E+00 |
| SurfaceAreatoVolumeRatio RV ES | Shape | 0.173 | 0.077 | 0.086 | 0.102 | 1.407E-13 | 2.700E-10 | 3.010E-13 | 7.267E-03 |
| SurfaceAreatoVolumeRatio LV ED | Shape | 0.341 | 0.181 | 0.067 | 0.103 | 1.790E-43 | 8.740E-49 | 4.827E-06 | 3.900E-02 |
| InformalMeasureofCorrelation1 glcm MYO ES | Shape | 0.297 | 0.079 | -0.015 | 0.115 | 4.388E-29 | 1.841E-07 | 1.000E+00 | 1.770E-02 |
| SurfaceAreatoVolumeRatio RV ED | Shape | 0.232 | 0.054 | 0.010 | 0.162 | 4.676E-26 | 5.766E-05 | 1.000E+00 | 3.329E-09 |
| LeastAxis RV ED | Size | -0.252 | -0.088 | 0.022 | -0.129 | 3.422E-30 | 2.513E-14 | 1.000E+00 | 2.179E-05 |
| Volume RV ED | Size | -0.313 | -0.124 | -0.035 | -0.121 | 2.838E-61 | 1.071E-37 | 3.462E-02 | 3.077E-06 |
| Max2DdiameterColumn RV ED | Size | -0.277 | -0.115 | 0.023 | -0.117 | 9.128E-35 | 1.575E-23 | 1.000E+00 | 5.697E-04 |
| MinorAxis RV ED | Size | -0.313 | -0.134 | -0.052 | -0.117 | 1.178E-38 | 4.663E-28 | 1.933E-03 | 2.771E-03 |
| MinorAxis RV ES | Size | -0.204 | -0.098 | -0.056 | -0.097 | 1.096E-16 | 4.487E-15 | 2.318E-04 | 4.751E-02 |
| GrayLevelNonUniformity glszm MYO ED | Size | -0.256 | -0.075 | 0.009 | -0.092 | 4.932E-20 | 3.657E-06 | 1.000E+00 | 4.998E-01 |
| SizeZoneNonUniformity glszm MYO ED | Size | -0.256 | -0.075 | 0.009 | -0.092 | 4.932E-20 | 3.657E-06 | 1.000E+00 | 4.998E-01 |
| SurfaceArea RV ED | Size | -0.322 | -0.152 | -0.047 | -0.091 | 1.617E-60 | 1.898E-53 | 2.974E-04 | 7.625E-03 |
| Max2DdiameterColumn RV ES | Size | -0.198 | -0.090 | -0.013 | -0.079 | 2.831E-16 | 2.754E-13 | 1.000E+00 | 4.689E-01 |
| LeastAxis RV ES | Size | -0.191 | -0.108 | -0.074 | -0.079 | 1.340E-17 | 2.462E-22 | 2.566E-10 | 2.257E-01 |
| LeastAxis LV ED | Size | -0.303 | -0.130 | 0.011 | -0.067 | 4.309E-41 | 6.156E-30 | 1.000E+00 | 1.000E+00 |
| Volume RV ES | Size | -0.208 | -0.109 | -0.093 | -0.063 | 2.820E-24 | 7.983E-27 | 2.228E-19 | 1.000E+00 |
| MinorAxis LV ED | Size | -0.233 | -0.115 | -0.001 | -0.059 | 1.362E-23 | 6.084E-23 | 1.000E+00 | 1.000E+00 |
| Max2DdiameterSlice LV ED | Size | -0.169 | -0.051 | 0.035 | -0.056 | 8.408E-10 | 5.758E-03 | 9.917E-01 | 1.000E+00 |
| Max2DdiameterRow RV ED | Size | -0.158 | -0.081 | -0.035 | -0.055 | 2.363E-09 | 8.733E-10 | 6.399E-01 | 1.000E+00 |
| SurfaceArea RV ES | Size | -0.211 | -0.125 | -0.087 | -0.047 | 1.520E-25 | 8.300E-36 | 3.741E-17 | 1.000E+00 |
| Volume LV ED | Size | -0.311 | -0.134 | 0.010 | -0.044 | 7.059E-55 | 2.449E-40 | 1.000E+00 | 1.000E+00 |
| Max2DdiameterSlice LV ES | Size | -0.153 | -0.022 | 0.044 | -0.040 | 1.711E-08 | 1.000E+00 | 5.100E-02 | 1.000E+00 |
| Max3Ddiameter LV ED | Size | -0.232 | -0.062 | 0.034 | -0.033 | 1.211E-24 | 2.018E-06 | 3.541E-01 | 1.000E+00 |
| LeastAxis LV ES | Size | -0.177 | -0.120 | -0.074 | -0.030 | 3.711E-13 | 1.833E-24 | 7.574E-09 | 1.000E+00 |
| Max2DdiameterRow LV ED | Size | -0.250 | -0.079 | 0.028 | -0.029 | 1.262E-29 | 2.076E-11 | 1.000E+00 | 1.000E+00 |
| Max3Ddiameter LV ES | Size | -0.179 | -0.046 | 0.061 | -0.025 | 1.413E-13 | 9.678E-03 | 9.424E-06 | 1.000E+00 |
| Max3Ddiameter RV ED | Size | -0.218 | -0.119 | -0.028 | -0.019 | 3.065E-22 | 2.294E-26 | 1.000E+00 | 1.000E+00 |
| MinorAxis LV ES | Size | -0.153 | -0.077 | -0.012 | -0.019 | 1.474E-09 | 1.042E-09 | 1.000E+00 | 1.000E+00 |
| Max2DdiameterRow LV ES | Size | -0.186 | -0.062 | 0.046 | -0.016 | 1.838E-15 | 2.265E-06 | 4.385E-03 | 1.000E+00 |
| SurfaceArea LV ED | Size | -0.270 | -0.094 | 0.050 | -0.016 | 1.186E-44 | 7.170E-21 | 2.598E-05 | 1.000E+00 |
| MajorAxis LV ES | Size | -0.158 | -0.046 | 0.075 | -0.012 | 1.281E-11 | 3.147E-03 | 3.275E-10 | 1.000E+00 |
| Max2DdiameterColumn LV ES | Size | -0.171 | -0.071 | 0.043 | -0.008 | 4.914E-13 | 1.057E-08 | 1.902E-02 | 1.000E+00 |
| MajorAxis LV ED | Size | -0.211 | -0.054 | 0.051 | -0.008 | 3.762E-22 | 4.804E-05 | 2.071E-04 | 1.000E+00 |
| Max2DdiameterColumn LV ED | Size | -0.250 | -0.079 | 0.031 | -0.007 | 1.947E-32 | 1.786E-12 | 5.010E-01 | 1.000E+00 |
| Volume LV ES | Size | -0.189 | -0.122 | -0.040 | -0.004 | 1.240E-16 | 3.493E-28 | 4.352E-02 | 1.000E+00 |
| SurfaceArea LV ES | Size | -0.182 | -0.089 | 0.005 | -0.001 | 4.569E-17 | 2.732E-16 | 1.000E+00 | 1.000E+00 |
| Max2DdiameterSlice RV ED | Size | -0.268 | -0.179 | -0.119 | 0.002 | 7.753E-25 | 3.032E-44 | 2.573E-19 | 1.000E+00 |
| Max2DdiameterRow RV ES | Size | -0.075 | -0.071 | -0.044 | 0.012 | 5.825E-01 | 2.238E-06 | 8.882E-02 | 1.000E+00 |
| MajorAxis RV ED | Size | -0.241 | -0.163 | -0.087 | 0.014 | 4.782E-26 | 1.054E-47 | 3.248E-13 | 1.000E+00 |
| RunLengthNonUniformity glrlm MYO ED | Size | -0.268 | -0.072 | 0.053 | 0.021 | 1.351E-23 | 3.930E-06 | 7.309E-03 | 1.000E+00 |
| RunLengthNonUniformity glrlm MYO ES | Size | -0.194 | -0.071 | 0.039 | 0.035 | 2.464E-11 | 1.262E-05 | 7.543E-01 | 1.000E+00 |
| Max3Ddiameter RV ES | Size | -0.129 | -0.085 | 0.005 | 0.044 | 5.825E-07 | 1.313E-12 | 1.000E+00 | 1.000E+00 |
| DependenceNonUniformity gldm MYO ED | Size | -0.246 | -0.082 | 0.083 | 0.048 | 2.926E-21 | 8.181E-09 | 3.156E-09 | 1.000E+00 |
| Max2DdiameterSlice RV ES | Size | -0.122 | -0.105 | -0.055 | 0.066 | 2.113E-04 | 1.154E-14 | 2.354E-03 | 1.000E+00 |
| GrayLevelNonUniformity glszm MYO ES | Size | 0.081 | 0.038 | 0.064 | 0.081 | 4.717E-01 | 8.153E-01 | 2.138E-04 | 1.000E+00 |
| SizeZoneNonUniformity glszm MYO ES | Size | 0.081 | 0.038 | 0.064 | 0.081 | 4.717E-01 | 8.153E-01 | 2.138E-04 | 1.000E+00 |
| MajorAxis RV ES | Size | -0.089 | -0.111 | -0.051 | 0.082 | 2.155E-02 | 1.159E-19 | 1.758E-03 | 4.006E-01 |

**Supplementary Table 6. Associations of vascular with radiomics features from linear regression models: Sex interactions (all results are for VRF *sex in fully adjusted models)**

|  |  | **Beta** | | | | **Adjusted p-value** | | | |
| --- | --- | --- | --- | --- | --- | --- | --- | --- | --- |
| **Feature** | **Cluster** | **Diabetes** | **High chol.** | **Hypertension** | **Smoker** | **Diabetes** | **High chol.** | **Hypertension** | **Smoker** |
| InterquartileRange MYO ES | Global Intensity | -0.096 | -0.013 | 0.008 | -0.116 | 1.000E+00 | 1.000E+00 | 1.000E+00 | 1.000E+00 |
| RobustMeanAbsoluteDeviation MYO ES | Global Intensity | -0.079 | -0.015 | 0.011 | -0.132 | 1.000E+00 | 1.000E+00 | 1.000E+00 | 1.000E+00 |
| Percentile90 MYO ES | Global Intensity | -0.028 | -0.012 | 0.003 | -0.063 | 1.000E+00 | 1.000E+00 | 1.000E+00 | 1.000E+00 |
| RootMeanSquared MYO ES | Global Intensity | -0.018 | -0.002 | 0.002 | -0.027 | 1.000E+00 | 1.000E+00 | 1.000E+00 | 1.000E+00 |
| MeanAbsoluteDeviation MYO ES | Global Intensity | -0.033 | 0.008 | 0.037 | -0.094 | 1.000E+00 | 1.000E+00 | 1.000E+00 | 1.000E+00 |
| Mean MYO ES | Global Intensity | -0.019 | -0.003 | 0.000 | -0.024 | 1.000E+00 | 1.000E+00 | 1.000E+00 | 1.000E+00 |
| Median MYO ES | Global Intensity | -0.015 | 0.004 | 0.004 | -0.032 | 1.000E+00 | 1.000E+00 | 1.000E+00 | 1.000E+00 |
| Entropy MYO ES | Global Intensity | -0.015 | 0.008 | 0.027 | -0.047 | 1.000E+00 | 1.000E+00 | 1.000E+00 | 1.000E+00 |
| SumEntropy glcm MYO ES | Global Intensity | -0.049 | -0.015 | -0.005 | -0.056 | 1.000E+00 | 1.000E+00 | 1.000E+00 | 1.000E+00 |
| Percentile10 MYO ES | Global Intensity | -0.006 | -0.005 | -0.016 | 0.013 | 1.000E+00 | 1.000E+00 | 1.000E+00 | 1.000E+00 |
| JointEntropy glcm MYO ES | Global Intensity | 0.009 | 0.018 | 0.027 | -0.021 | 1.000E+00 | 1.000E+00 | 1.000E+00 | 1.000E+00 |
| Percentile10 MYO ED | Global Intensity | -0.043 | -0.039 | -0.074 | 0.021 | 1.000E+00 | 1.000E+00 | 9.554E-01 | 1.000E+00 |
| Mean MYO ED | Global Intensity | 0.022 | -0.002 | 0.006 | 0.048 | 1.000E+00 | 1.000E+00 | 1.000E+00 | 1.000E+00 |
| Percentile90 MYO ED | Global Intensity | 0.060 | 0.031 | 0.064 | 0.071 | 1.000E+00 | 1.000E+00 | 1.000E+00 | 1.000E+00 |
| RootMeanSquared MYO ED | Global Intensity | 0.028 | 0.004 | 0.020 | 0.053 | 1.000E+00 | 1.000E+00 | 1.000E+00 | 1.000E+00 |
| Maximum MYO ED | Global Intensity | 0.058 | 0.043 | 0.081 | 0.112 | 1.000E+00 | 1.000E+00 | 2.327E-01 | 1.000E+00 |
| Median MYO ED | Global Intensity | 0.020 | -0.009 | -0.011 | 0.029 | 1.000E+00 | 1.000E+00 | 1.000E+00 | 1.000E+00 |
| SmallAreaHighGrayLevelEmphasis glszm MYO ES | Global Intensity | -0.002 | 0.059 | 0.048 | 0.070 | 1.000E+00 | 1.000E+00 | 1.000E+00 | 1.000E+00 |
| Variance MYO ES | Global Intensity | 0.030 | 0.020 | 0.051 | -0.066 | 1.000E+00 | 1.000E+00 | 1.000E+00 | 1.000E+00 |
| GrayLevelVariance gldm MYO ES | Global Intensity | 0.024 | 0.010 | 0.036 | -0.046 | 1.000E+00 | 1.000E+00 | 1.000E+00 | 1.000E+00 |
| GrayLevelVariance glrlm MYO ES | Global Intensity | 0.080 | 0.036 | 0.052 | -0.041 | 1.000E+00 | 1.000E+00 | 1.000E+00 | 1.000E+00 |
| Energy MYO ES | Global Intensity | -0.213 | -0.126 | -0.086 | -0.047 | 7.823E-03 | 5.082E-05 | 1.184E-01 | 1.000E+00 |
| ClusterTendency glcm MYO ES | Global Intensity | -0.038 | -0.032 | -0.006 | -0.093 | 1.000E+00 | 1.000E+00 | 1.000E+00 | 1.000E+00 |
| SumofSquares glcm MYO ES | Global Intensity | 0.000 | -0.008 | 0.015 | -0.048 | 1.000E+00 | 1.000E+00 | 1.000E+00 | 1.000E+00 |
| SmallDependenceHighGrayLevelEmphasis gldm MYO ES | Global Intensity | 0.115 | 0.130 | 0.135 | 0.028 | 1.000E+00 | 2.231E-05 | 1.089E-05 | 1.000E+00 |
| Complexity ngtdm MYO ED | Global Intensity | 0.119 | 0.066 | 0.127 | 0.082 | 1.000E+00 | 1.000E+00 | 3.585E-05 | 1.000E+00 |
| Range MYO ED | Global Intensity | 0.079 | 0.052 | 0.092 | 0.107 | 1.000E+00 | 1.000E+00 | 3.948E-02 | 1.000E+00 |
| SmallDependenceHighGrayLevelEmphasis gldm MYO ED | Global Intensity | 0.133 | 0.084 | 0.159 | 0.013 | 8.480E-01 | 2.267E-02 | 1.270E-10 | 1.000E+00 |
| Strength ngtdm MYO ED | Global Intensity | 0.084 | 0.023 | 0.117 | 0.120 | 1.000E+00 | 1.000E+00 | 2.934E-04 | 1.000E+00 |
| HighGrayLevelZoneEmphasis glszm MYO ES | Global Intensity | 0.022 | 0.039 | 0.027 | 0.033 | 1.000E+00 | 1.000E+00 | 1.000E+00 | 1.000E+00 |
| TotalEnergy MYO ES | Global Intensity | -0.189 | -0.114 | -0.065 | -0.032 | 4.054E-02 | 4.338E-04 | 1.000E+00 | 1.000E+00 |
| ZoneEntropy glszm MYO ES | Global Intensity | 0.047 | 0.046 | -0.014 | -0.044 | 1.000E+00 | 1.000E+00 | 1.000E+00 | 1.000E+00 |
| SmallAreaHighGrayLevelEmphasis glszm MYO ED | Global Intensity | 0.083 | 0.032 | 0.034 | -0.018 | 1.000E+00 | 1.000E+00 | 1.000E+00 | 1.000E+00 |
| HighGrayLevelZoneEmphasis glszm MYO ED | Global Intensity | 0.054 | 0.004 | -0.004 | -0.038 | 1.000E+00 | 1.000E+00 | 1.000E+00 | 1.000E+00 |
| Energy MYO ED | Global Intensity | -0.139 | -0.083 | -0.051 | 0.020 | 1.000E+00 | 2.010E-01 | 1.000E+00 | 1.000E+00 |
| ShortRunHighGrayLevelEmphasis glrlm MYO ES | Global Intensity | 0.100 | 0.080 | 0.061 | -0.033 | 1.000E+00 | 3.722E-01 | 1.000E+00 | 1.000E+00 |
| Autocorrelation glcm MYO ES | Global Intensity | 0.108 | 0.070 | 0.041 | -0.060 | 1.000E+00 | 1.000E+00 | 1.000E+00 | 1.000E+00 |
| SumAverage glcm MYO ES | Global Intensity | 0.112 | 0.073 | 0.041 | -0.044 | 1.000E+00 | 9.546E-01 | 1.000E+00 | 1.000E+00 |
| JointAverage glcm MYO ES | Global Intensity | 0.112 | 0.073 | 0.041 | -0.044 | 1.000E+00 | 9.546E-01 | 1.000E+00 | 1.000E+00 |
| HighGrayLevelEmphasis gldm MYO ES | Global Intensity | 0.104 | 0.068 | 0.040 | -0.054 | 1.000E+00 | 1.000E+00 | 1.000E+00 | 1.000E+00 |
| HighGrayLevelRunEmphasis glrlm MYO ES | Global Intensity | 0.085 | 0.061 | 0.037 | -0.049 | 1.000E+00 | 1.000E+00 | 1.000E+00 | 1.000E+00 |
| TotalEnergy MYO ED | Global Intensity | -0.108 | -0.066 | -0.023 | 0.040 | 1.000E+00 | 1.000E+00 | 1.000E+00 | 1.000E+00 |
| ShortRunHighGrayLevelEmphasis glrlm MYO ED | Global Intensity | 0.062 | 0.007 | 0.006 | -0.076 | 1.000E+00 | 1.000E+00 | 1.000E+00 | 1.000E+00 |
| RunEntropy glrlm MYO ED | Global Intensity | 0.073 | -0.027 | 0.070 | 0.122 | 1.000E+00 | 1.000E+00 | 1.000E+00 | 1.000E+00 |
| HighGrayLevelRunEmphasis glrlm MYO ED | Global Intensity | 0.054 | -0.011 | -0.020 | -0.092 | 1.000E+00 | 1.000E+00 | 1.000E+00 | 1.000E+00 |
| HighGrayLevelEmphasis gldm MYO ED | Global Intensity | 0.064 | -0.006 | -0.017 | -0.095 | 1.000E+00 | 1.000E+00 | 1.000E+00 | 1.000E+00 |
| InformalMeasureofCorrelation2 glcm MYO ED | Global Intensity | -0.023 | -0.006 | 0.045 | 0.049 | 1.000E+00 | 1.000E+00 | 1.000E+00 | 1.000E+00 |
| DependenceEntropy gldm MYO ED | Global Intensity | 0.003 | -0.030 | -0.060 | 0.075 | 1.000E+00 | 1.000E+00 | 1.000E+00 | 1.000E+00 |
| JointAverage glcm MYO ED | Global Intensity | 0.071 | -0.001 | -0.028 | -0.124 | 1.000E+00 | 1.000E+00 | 1.000E+00 | 1.000E+00 |
| SumAverage glcm MYO ED | Global Intensity | 0.071 | -0.001 | -0.028 | -0.124 | 1.000E+00 | 1.000E+00 | 1.000E+00 | 1.000E+00 |
| Autocorrelation glcm MYO ED | Global Intensity | 0.067 | -0.002 | -0.024 | -0.126 | 1.000E+00 | 1.000E+00 | 1.000E+00 | 1.000E+00 |
| Correlation glcm MYO ED | Global Intensity | -0.073 | -0.051 | -0.034 | 0.077 | 1.000E+00 | 1.000E+00 | 1.000E+00 | 1.000E+00 |
| ZonePercentage glszm MYO ED | Global Variance | 0.112 | 0.119 | 0.214 | 0.048 | 1.000E+00 | 4.701E-07 | 9.110E-24 | 1.000E+00 |
| DifferenceVariance glcm MYO ED | Global Variance | 0.154 | 0.119 | 0.197 | 0.072 | 1.818E-01 | 9.108E-06 | 1.125E-16 | 1.000E+00 |
| SmallDependenceEmphasis gldm MYO ED | Global Variance | 0.122 | 0.125 | 0.203 | 0.057 | 7.291E-01 | 3.686E-08 | 5.856E-22 | 1.000E+00 |
| DifferenceEntropy glcm MYO ED | Global Variance | 0.129 | 0.118 | 0.147 | 0.047 | 6.789E-01 | 1.591E-06 | 3.850E-10 | 1.000E+00 |
| Contrast glcm MYO ED | Global Variance | 0.155 | 0.115 | 0.197 | 0.072 | 1.358E-01 | 1.664E-05 | 2.664E-17 | 1.000E+00 |
| SmallDependenceEmphasis gldm MYO ES | Global Variance | 0.091 | 0.150 | 0.161 | 0.039 | 1.000E+00 | 1.287E-08 | 9.656E-10 | 1.000E+00 |
| ZonePercentage glszm MYO ES | Global Variance | 0.096 | 0.152 | 0.160 | 0.009 | 1.000E+00 | 1.220E-08 | 2.917E-09 | 1.000E+00 |
| ShortRunEmphasis glrlm MYO ED | Global Variance | 0.091 | 0.113 | 0.088 | 0.023 | 1.000E+00 | 5.545E-06 | 4.173E-03 | 1.000E+00 |
| DifferenceEntropy glcm MYO ES | Global Variance | 0.095 | 0.076 | 0.072 | 0.062 | 1.000E+00 | 2.582E-01 | 5.926E-01 | 1.000E+00 |
| RunLengthNonUniformityNormalized glrlm MYO ED | Global Variance | 0.087 | 0.110 | 0.104 | 0.030 | 1.000E+00 | 1.350E-05 | 1.300E-04 | 1.000E+00 |
| RunPercentage glrlm MYO ES | Global Variance | 0.059 | 0.070 | 0.057 | 0.034 | 1.000E+00 | 5.112E-01 | 1.000E+00 | 1.000E+00 |
| DifferenceAverage glcm MYO ED | Global Variance | 0.141 | 0.113 | 0.159 | 0.059 | 2.856E-01 | 8.751E-06 | 8.939E-12 | 1.000E+00 |
| Contrast ngtdm MYO ES | Global Variance | 0.001 | -0.048 | -0.002 | -0.059 | 1.000E+00 | 1.000E+00 | 1.000E+00 | 1.000E+00 |
| RunPercentage glrlm MYO ED | Global Variance | 0.084 | 0.107 | 0.081 | 0.012 | 1.000E+00 | 2.827E-05 | 1.963E-02 | 1.000E+00 |
| Contrast ngtdm MYO ED | Global Variance | 0.099 | 0.085 | 0.153 | 0.095 | 1.000E+00 | 3.944E-02 | 5.792E-09 | 1.000E+00 |
| DependenceNonUniformityNormalized gldm MYO ES | Global Variance | -0.047 | 0.005 | 0.063 | 0.042 | 1.000E+00 | 1.000E+00 | 1.000E+00 | 1.000E+00 |
| DifferenceVariance glcm MYO ES | Global Variance | 0.096 | 0.064 | 0.068 | 0.070 | 1.000E+00 | 1.000E+00 | 1.000E+00 | 1.000E+00 |
| MeanAbsoluteDeviation MYO ED | Global Variance | 0.099 | 0.061 | 0.126 | 0.096 | 1.000E+00 | 1.000E+00 | 2.654E-06 | 1.000E+00 |
| DifferenceAverage glcm MYO ES | Global Variance | 0.094 | 0.059 | 0.064 | 0.075 | 1.000E+00 | 1.000E+00 | 1.000E+00 | 1.000E+00 |
| InverseVariance glcm MYO ES | Global Variance | 0.057 | 0.035 | 0.031 | 0.050 | 1.000E+00 | 1.000E+00 | 1.000E+00 | 1.000E+00 |
| GrayLevelVariance glrlm MYO ED | Global Variance | 0.100 | 0.044 | 0.133 | 0.076 | 1.000E+00 | 1.000E+00 | 2.393E-06 | 1.000E+00 |
| Entropy MYO ED | Global Variance | 0.111 | 0.083 | 0.104 | 0.073 | 1.000E+00 | 2.065E-02 | 2.877E-04 | 1.000E+00 |
| ShortRunEmphasis glrlm MYO ES | Global Variance | 0.105 | 0.096 | 0.074 | 0.042 | 1.000E+00 | 8.475E-03 | 3.830E-01 | 1.000E+00 |
| Contrast glcm MYO ES | Global Variance | 0.098 | 0.056 | 0.064 | 0.080 | 1.000E+00 | 1.000E+00 | 1.000E+00 | 1.000E+00 |
| Variance MYO ED | Global Variance | 0.092 | 0.043 | 0.134 | 0.075 | 1.000E+00 | 1.000E+00 | 1.448E-06 | 1.000E+00 |
| GrayLevelVariance gldm MYO ED | Global Variance | 0.097 | 0.048 | 0.134 | 0.072 | 1.000E+00 | 1.000E+00 | 1.322E-06 | 1.000E+00 |
| JointEntropy glcm MYO ED | Global Variance | 0.123 | 0.102 | 0.119 | 0.052 | 1.000E+00 | 3.426E-04 | 9.240E-06 | 1.000E+00 |
| RunLengthNonUniformityNormalized glrlm MYO ES | Global Variance | 0.089 | 0.088 | 0.080 | 0.043 | 1.000E+00 | 4.097E-02 | 1.680E-01 | 1.000E+00 |
| SumofSquares glcm MYO ED | Global Variance | 0.118 | 0.083 | 0.161 | 0.054 | 1.000E+00 | 5.754E-02 | 4.140E-10 | 1.000E+00 |
| DependenceNonUniformityNormalized gldm MYO ED | Global Variance | 0.083 | 0.099 | 0.155 | 0.035 | 1.000E+00 | 9.820E-04 | 2.489E-10 | 1.000E+00 |
| RobustMeanAbsoluteDeviation MYO ED | Global Variance | 0.112 | 0.055 | 0.122 | 0.097 | 1.000E+00 | 1.000E+00 | 1.587E-05 | 1.000E+00 |
| SumEntropy glcm MYO ED | Global Variance | 0.108 | 0.098 | 0.103 | 0.049 | 1.000E+00 | 2.000E-03 | 8.887E-04 | 1.000E+00 |
| ZoneEntropy glszm MYO ED | Global Variance | 0.053 | 0.030 | 0.035 | 0.018 | 1.000E+00 | 1.000E+00 | 1.000E+00 | 1.000E+00 |
| InterquartileRange MYO ED | Global Variance | 0.099 | 0.048 | 0.105 | 0.097 | 1.000E+00 | 1.000E+00 | 1.117E-03 | 1.000E+00 |
| ClusterTendency glcm MYO ED | Global Variance | 0.096 | 0.064 | 0.138 | 0.043 | 1.000E+00 | 1.000E+00 | 1.161E-06 | 1.000E+00 |
| InverseVariance glcm MYO ED | Global Variance | 0.083 | 0.082 | 0.020 | -0.001 | 1.000E+00 | 6.460E-02 | 1.000E+00 | 1.000E+00 |
| GrayLevelVariance glszm MYO ED | Global Variance | 0.107 | 0.047 | 0.126 | 0.054 | 1.000E+00 | 1.000E+00 | 4.518E-05 | 1.000E+00 |
| SmallDependenceLowGrayLevelEmphasis gldm MYO ED | Local Dimness | 0.015 | 0.088 | 0.155 | 0.124 | 1.000E+00 | 1.223E-01 | 3.683E-07 | 1.000E+00 |
| ShortRunLowGrayLevelEmphasis glrlm MYO ED | Local Dimness | 0.006 | 0.046 | 0.096 | 0.111 | 1.000E+00 | 1.000E+00 | 6.618E-02 | 1.000E+00 |
| LowGrayLevelEmphasis gldm MYO ED | Local Dimness | -0.029 | 0.024 | 0.076 | 0.113 | 1.000E+00 | 1.000E+00 | 9.119E-01 | 1.000E+00 |
| LowGrayLevelRunEmphasis glrlm MYO ED | Local Dimness | -0.015 | 0.027 | 0.081 | 0.107 | 1.000E+00 | 1.000E+00 | 4.577E-01 | 1.000E+00 |
| SmallDependenceLowGrayLevelEmphasis gldm MYO ES | Local Dimness | 0.042 | 0.048 | 0.059 | -0.013 | 1.000E+00 | 1.000E+00 | 1.000E+00 | 1.000E+00 |
| ShortRunLowGrayLevelEmphasis glrlm MYO ES | Local Dimness | 0.020 | -0.023 | -0.009 | 0.000 | 1.000E+00 | 1.000E+00 | 1.000E+00 | 1.000E+00 |
| LargeDependenceLowGrayLevelEmphasis gldm MYO ED | Local Dimness | -0.096 | -0.050 | 0.028 | 0.083 | 1.000E+00 | 1.000E+00 | 1.000E+00 | 1.000E+00 |
| LowGrayLevelEmphasis gldm MYO ES | Local Dimness | -0.059 | -0.064 | -0.024 | 0.007 | 1.000E+00 | 1.000E+00 | 1.000E+00 | 1.000E+00 |
| LowGrayLevelRunEmphasis glrlm MYO ES | Local Dimness | -0.016 | -0.045 | -0.016 | 0.000 | 1.000E+00 | 1.000E+00 | 1.000E+00 | 1.000E+00 |
| LowGrayLevelZoneEmphasis glszm MYO ED | Local Dimness | -0.029 | 0.019 | 0.077 | 0.078 | 1.000E+00 | 1.000E+00 | 7.147E-01 | 1.000E+00 |
| SmallAreaLowGrayLevelEmphasis glszm MYO ED | Local Dimness | -0.066 | 0.008 | 0.055 | 0.090 | 1.000E+00 | 1.000E+00 | 1.000E+00 | 1.000E+00 |
| LongRunLowGrayLevelEmphasis glrlm MYO ED | Local Dimness | -0.058 | -0.033 | 0.064 | 0.103 | 1.000E+00 | 1.000E+00 | 1.000E+00 | 1.000E+00 |
| SmallAreaLowGrayLevelEmphasis glszm MYO ES | Local Dimness | 0.008 | 0.003 | 0.013 | -0.048 | 1.000E+00 | 1.000E+00 | 1.000E+00 | 1.000E+00 |
| LowGrayLevelZoneEmphasis glszm MYO ES | Local Dimness | 0.037 | 0.000 | 0.005 | -0.052 | 1.000E+00 | 1.000E+00 | 1.000E+00 | 1.000E+00 |
| Busyness ngtdm MYO ED | Local Dimness | -0.095 | -0.018 | 0.024 | 0.095 | 1.000E+00 | 1.000E+00 | 1.000E+00 | 1.000E+00 |
| LargeDependenceLowGrayLevelEmphasis gldm MYO ES | Local Dimness | -0.080 | -0.072 | -0.007 | -0.004 | 1.000E+00 | 7.145E-01 | 1.000E+00 | 1.000E+00 |
| Busyness ngtdm MYO ES | Local Dimness | -0.107 | -0.075 | -0.015 | 0.003 | 1.000E+00 | 3.688E-01 | 1.000E+00 | 1.000E+00 |
| LongRunLowGrayLevelEmphasis glrlm MYO ES | Local Dimness | -0.055 | -0.058 | 0.031 | 0.009 | 1.000E+00 | 1.000E+00 | 1.000E+00 | 1.000E+00 |
| LargeAreaLowGrayLevelEmphasis glszm MYO ED | Local Dimness | 0.000 | -0.046 | 0.074 | 0.147 | 1.000E+00 | 1.000E+00 | 4.778E-01 | 1.000E+00 |
| LargeAreaLowGrayLevelEmphasis glszm MYO ES | Local Dimness | 0.023 | -0.052 | 0.103 | 0.085 | 1.000E+00 | 1.000E+00 | 4.530E-03 | 1.000E+00 |
| InformalMeasureofCorrelation2 glcm MYO ES | Local Uniformity | -0.227 | -0.120 | -0.079 | -0.136 | 3.187E-03 | 3.043E-04 | 4.160E-01 | 1.000E+00 |
| DependenceEntropy gldm MYO ES | Local Uniformity | -0.171 | -0.078 | -0.099 | -0.087 | 3.696E-01 | 5.508E-01 | 3.415E-02 | 1.000E+00 |
| Correlation glcm MYO ES | Local Uniformity | -0.223 | -0.123 | -0.086 | -0.151 | 2.526E-03 | 7.470E-05 | 1.011E-01 | 1.000E+00 |
| RunEntropy glrlm MYO ES | Local Uniformity | -0.067 | -0.062 | -0.024 | -0.097 | 1.000E+00 | 1.000E+00 | 1.000E+00 | 1.000E+00 |
| InverseDifferenceMomentNormalized glcm MYO ES | Local Uniformity | -0.039 | 0.028 | 0.009 | 0.029 | 1.000E+00 | 1.000E+00 | 1.000E+00 | 1.000E+00 |
| GrayLevelNonUniformityNormalized glszm MYO ED | Local Uniformity | -0.063 | -0.055 | -0.081 | -0.009 | 1.000E+00 | 1.000E+00 | 1.567E-01 | 1.000E+00 |
| MaximumProbability glcm MYO ED | Local Uniformity | -0.066 | -0.080 | -0.057 | -0.034 | 1.000E+00 | 1.043E-01 | 1.000E+00 | 1.000E+00 |
| InverseDifferenceNormalized glcm MYO ES | Local Uniformity | -0.051 | 0.000 | -0.013 | -0.003 | 1.000E+00 | 1.000E+00 | 1.000E+00 | 1.000E+00 |
| JointEnergy glcm MYO ED | Local Uniformity | -0.079 | -0.093 | -0.062 | -0.026 | 1.000E+00 | 8.888E-03 | 1.000E+00 | 1.000E+00 |
| LargeDependenceHighGrayLevelEmphasis gldm MYO ES | Local Uniformity | 0.062 | 0.014 | -0.023 | -0.066 | 1.000E+00 | 1.000E+00 | 1.000E+00 | 1.000E+00 |
| DependenceVariance gldm MYO ED | Local Uniformity | -0.079 | -0.096 | -0.075 | 0.006 | 1.000E+00 | 1.973E-03 | 1.680E-01 | 1.000E+00 |
| Uniformity MYO ED | Local Uniformity | -0.088 | -0.083 | -0.076 | -0.059 | 1.000E+00 | 3.461E-02 | 1.578E-01 | 1.000E+00 |
| DependenceNonUniformity gldm MYO ES | Local Uniformity | -0.218 | -0.120 | -0.035 | 0.002 | 1.064E-05 | 6.155E-08 | 1.000E+00 | 1.000E+00 |
| InverseDifferenceNormalized glcm MYO ED | Local Uniformity | -0.088 | -0.107 | -0.103 | -0.024 | 1.000E+00 | 7.545E-04 | 2.634E-03 | 1.000E+00 |
| GrayLevelNonUniformityNormalized glrlm MYO ED | Local Uniformity | -0.111 | -0.080 | -0.093 | -0.066 | 1.000E+00 | 4.914E-02 | 5.266E-03 | 1.000E+00 |
| GrayLevelNonUniformity gldm MYO ED | Local Uniformity | -0.132 | -0.118 | -0.013 | 0.004 | 9.169E-02 | 6.423E-09 | 1.000E+00 | 1.000E+00 |
| LongRunHighGrayLevelEmphasis glrlm MYO ES | Local Uniformity | 0.017 | -0.010 | -0.007 | -0.057 | 1.000E+00 | 1.000E+00 | 1.000E+00 | 1.000E+00 |
| InverseDifference glcm MYO ED | Local Uniformity | -0.126 | -0.107 | -0.123 | -0.043 | 9.403E-01 | 5.313E-05 | 1.437E-06 | 1.000E+00 |
| InverseDifference glcm MYO ES | Local Uniformity | -0.085 | -0.054 | -0.057 | -0.066 | 1.000E+00 | 1.000E+00 | 1.000E+00 | 1.000E+00 |
| InverseDifferenceMoment glcm MYO ES | Local Uniformity | -0.089 | -0.057 | -0.061 | -0.069 | 1.000E+00 | 1.000E+00 | 1.000E+00 | 1.000E+00 |
| LargeDependenceEmphasis gldm MYO ED | Local Uniformity | -0.072 | -0.105 | -0.048 | 0.006 | 1.000E+00 | 1.009E-04 | 1.000E+00 | 1.000E+00 |
| InverseDifferenceMoment glcm MYO ED | Local Uniformity | -0.129 | -0.109 | -0.131 | -0.047 | 7.357E-01 | 3.245E-05 | 8.874E-08 | 1.000E+00 |
| GrayLevelNonUniformity glrlm MYO ED | Local Uniformity | -0.180 | -0.104 | -0.056 | -0.024 | 1.006E-04 | 1.569E-07 | 2.790E-01 | 1.000E+00 |
| InverseDifferenceMomentNormalized glcm MYO ED | Local Uniformity | -0.065 | -0.099 | -0.115 | -0.035 | 1.000E+00 | 1.368E-02 | 1.077E-03 | 1.000E+00 |
| RunVariance glrlm MYO ED | Local Uniformity | -0.056 | -0.098 | 0.011 | 0.027 | 1.000E+00 | 5.757E-04 | 1.000E+00 | 1.000E+00 |
| LongRunEmphasis glrlm MYO ED | Local Uniformity | -0.058 | -0.101 | 0.002 | 0.028 | 1.000E+00 | 2.754E-04 | 1.000E+00 | 1.000E+00 |
| LargeDependenceEmphasis gldm MYO ES | Local Uniformity | -0.035 | -0.053 | -0.032 | -0.027 | 1.000E+00 | 1.000E+00 | 1.000E+00 | 1.000E+00 |
| ZoneVariance glszm MYO ED | Local Uniformity | -0.037 | -0.105 | 0.053 | 0.075 | 1.000E+00 | 5.733E-05 | 1.000E+00 | 1.000E+00 |
| LargeAreaEmphasis glszm MYO ED | Local Uniformity | -0.035 | -0.104 | 0.053 | 0.077 | 1.000E+00 | 8.555E-05 | 1.000E+00 | 1.000E+00 |
| DependenceVariance gldm MYO ES | Local Uniformity | 0.057 | 0.019 | -0.017 | -0.026 | 1.000E+00 | 1.000E+00 | 1.000E+00 | 1.000E+00 |
| LongRunEmphasis glrlm MYO ES | Local Uniformity | -0.045 | -0.053 | 0.019 | -0.018 | 1.000E+00 | 1.000E+00 | 1.000E+00 | 1.000E+00 |
| RunVariance glrlm MYO ES | Local Uniformity | -0.027 | -0.045 | 0.032 | -0.018 | 1.000E+00 | 1.000E+00 | 1.000E+00 | 1.000E+00 |
| LargeAreaHighGrayLevelEmphasis glszm MYO ES | Local Uniformity | -0.008 | -0.079 | 0.024 | 0.036 | 1.000E+00 | 1.048E-01 | 1.000E+00 | 1.000E+00 |
| LargeAreaHighGrayLevelEmphasis glszm MYO ED | Local Uniformity | -0.037 | -0.111 | 0.010 | 0.023 | 1.000E+00 | 2.840E-05 | 1.000E+00 | 1.000E+00 |
| MaximumProbability glcm MYO ES | Local Uniformity | 0.146 | 0.049 | 0.037 | 0.003 | 1.000E+00 | 1.000E+00 | 1.000E+00 | 1.000E+00 |
| LargeDependenceHighGrayLevelEmphasis gldm MYO ED | Local Uniformity | 0.002 | -0.090 | -0.103 | -0.076 | 1.000E+00 | 3.940E-02 | 5.103E-03 | 1.000E+00 |
| LargeAreaEmphasis glszm MYO ES | Local Uniformity | 0.013 | -0.073 | 0.086 | 0.085 | 1.000E+00 | 1.423E-01 | 1.877E-02 | 1.000E+00 |
| JointEnergy glcm MYO ES | Local Uniformity | 0.115 | 0.035 | 0.024 | 0.011 | 1.000E+00 | 1.000E+00 | 1.000E+00 | 1.000E+00 |
| ZoneVariance glszm MYO ES | Local Uniformity | 0.013 | -0.072 | 0.087 | 0.086 | 1.000E+00 | 1.755E-01 | 1.237E-02 | 1.000E+00 |
| LongRunHighGrayLevelEmphasis glrlm MYO ED | Local Uniformity | 0.004 | -0.092 | -0.063 | -0.077 | 1.000E+00 | 4.637E-02 | 1.000E+00 | 1.000E+00 |
| GrayLevelNonUniformity glrlm MYO ES | Local Uniformity | -0.127 | -0.063 | -0.034 | 0.057 | 2.285E-01 | 1.195E-01 | 1.000E+00 | 1.000E+00 |
| GrayLevelNonUniformity gldm MYO ES | Local Uniformity | 0.001 | -0.027 | 0.041 | 0.051 | 1.000E+00 | 1.000E+00 | 1.000E+00 | 1.000E+00 |
| GrayLevelNonUniformityNormalized glszm MYO ES | Local Uniformity | 0.133 | 0.002 | 0.017 | 0.026 | 1.000E+00 | 1.000E+00 | 1.000E+00 | 1.000E+00 |
| Uniformity MYO ES | Local Uniformity | 0.124 | 0.041 | 0.018 | 0.044 | 1.000E+00 | 1.000E+00 | 1.000E+00 | 1.000E+00 |
| GrayLevelNonUniformityNormalized glrlm MYO ES | Local Uniformity | 0.000 | -0.007 | -0.023 | 0.077 | 1.000E+00 | 1.000E+00 | 1.000E+00 | 1.000E+00 |
| Flatness RV ES | Shape | 0.074 | 0.109 | 0.015 | 0.077 | 1.000E+00 | 4.988E-03 | 1.000E+00 | 1.000E+00 |
| Elongation RV ES | Shape | -0.097 | -0.025 | -0.063 | 0.110 | 1.000E+00 | 1.000E+00 | 1.000E+00 | 1.000E+00 |
| Minimum MYO ED | Shape | -0.091 | -0.031 | -0.029 | 0.063 | 1.000E+00 | 1.000E+00 | 1.000E+00 | 1.000E+00 |
| Elongation RV ED | Shape | -0.153 | -0.066 | -0.078 | 0.137 | 1.000E+00 | 1.000E+00 | 7.796E-01 | 1.000E+00 |
| Flatness RV ED | Shape | -0.008 | -0.008 | -0.023 | 0.038 | 1.000E+00 | 1.000E+00 | 1.000E+00 | 1.000E+00 |
| InformalMeasureofCorrelation1 glcm MYO ED | Shape | 0.110 | 0.073 | 0.008 | -0.047 | 1.000E+00 | 7.271E-01 | 1.000E+00 | 1.000E+00 |
| Minimum MYO ES | Shape | -0.123 | -0.091 | -0.038 | -0.003 | 1.000E+00 | 1.053E-01 | 1.000E+00 | 1.000E+00 |
| Maximum MYO ES | Shape | 0.027 | 0.040 | 0.046 | 0.084 | 1.000E+00 | 1.000E+00 | 1.000E+00 | 1.000E+00 |
| Sphericity RV ED | Shape | -0.012 | -0.021 | 0.002 | -0.024 | 1.000E+00 | 1.000E+00 | 1.000E+00 | 1.000E+00 |
| Sphericity LV ED | Shape | 0.190 | 0.028 | 0.019 | 0.084 | 6.455E-02 | 1.000E+00 | 1.000E+00 | 1.000E+00 |
| Skewness MYO ED | Shape | -0.083 | -0.019 | 0.013 | 0.098 | 1.000E+00 | 1.000E+00 | 1.000E+00 | 1.000E+00 |
| Sphericity RV ES | Shape | 0.140 | 0.043 | 0.025 | 0.129 | 1.000E+00 | 1.000E+00 | 1.000E+00 | 1.000E+00 |
| GrayLevelVariance glszm MYO ES | Shape | 0.159 | 0.114 | 0.103 | 0.075 | 5.318E-01 | 9.663E-04 | 9.896E-03 | 1.000E+00 |
| Range MYO ES | Shape | 0.068 | 0.072 | 0.062 | 0.093 | 1.000E+00 | 9.538E-01 | 1.000E+00 | 1.000E+00 |
| SmallAreaEmphasis glszm MYO ES | Shape | -0.186 | 0.032 | 0.050 | 0.084 | 1.632E-01 | 1.000E+00 | 1.000E+00 | 1.000E+00 |
| Strength ngtdm MYO ES | Shape | 0.112 | 0.087 | 0.104 | 0.090 | 1.000E+00 | 1.051E-01 | 8.655E-03 | 1.000E+00 |
| SizeZoneNonUniformityNormalized glszm MYO ES | Shape | -0.178 | 0.023 | 0.046 | 0.087 | 2.793E-01 | 1.000E+00 | 1.000E+00 | 1.000E+00 |
| Complexity ngtdm MYO ES | Shape | 0.099 | 0.077 | 0.057 | 0.117 | 1.000E+00 | 5.149E-01 | 1.000E+00 | 1.000E+00 |
| Coarseness ngtdm MYO ES | Shape | 0.168 | 0.066 | 0.155 | -0.019 | 8.032E-02 | 7.504E-01 | 1.542E-09 | 1.000E+00 |
| Flatness LV ED | Shape | -0.060 | -0.065 | -0.110 | 0.115 | 1.000E+00 | 1.000E+00 | 5.279E-03 | 1.000E+00 |
| Elongation LV ED | Shape | -0.044 | -0.010 | -0.049 | 0.021 | 1.000E+00 | 1.000E+00 | 1.000E+00 | 1.000E+00 |
| Sphericity LV ES | Shape | 0.203 | 0.045 | 0.052 | 0.064 | 1.932E-02 | 1.000E+00 | 1.000E+00 | 1.000E+00 |
| ClusterShade glcm MYO ED | Shape | 0.034 | 0.020 | 0.111 | 0.007 | 1.000E+00 | 1.000E+00 | 3.348E-03 | 1.000E+00 |
| Kurtosis MYO ED | Shape | -0.078 | -0.027 | 0.012 | 0.068 | 1.000E+00 | 1.000E+00 | 1.000E+00 | 1.000E+00 |
| Coarseness ngtdm MYO ED | Shape | 0.143 | 0.021 | 0.117 | 0.093 | 1.470E-01 | 1.000E+00 | 1.583E-06 | 1.000E+00 |
| Flatness LV ES | Shape | 0.051 | 0.034 | 0.008 | 0.078 | 1.000E+00 | 1.000E+00 | 1.000E+00 | 1.000E+00 |
| Skewness MYO ES | Shape | -0.019 | -0.016 | 0.002 | 0.151 | 1.000E+00 | 1.000E+00 | 1.000E+00 | 1.000E+00 |
| Elongation LV ES | Shape | 0.043 | 0.031 | 0.016 | 0.029 | 1.000E+00 | 1.000E+00 | 1.000E+00 | 1.000E+00 |
| SmallAreaEmphasis glszm MYO ED | Shape | 0.029 | 0.031 | 0.066 | 0.072 | 1.000E+00 | 1.000E+00 | 1.000E+00 | 1.000E+00 |
| SizeZoneNonUniformityNormalized glszm MYO ED | Shape | 0.033 | 0.031 | 0.072 | 0.065 | 1.000E+00 | 1.000E+00 | 1.000E+00 | 1.000E+00 |
| ClusterProminence glcm MYO ED | Shape | 0.022 | 0.008 | 0.080 | -0.052 | 1.000E+00 | 1.000E+00 | 5.399E-01 | 1.000E+00 |
| SurfaceAreatoVolumeRatio LV ES | Shape | -0.040 | -0.046 | -0.004 | -0.007 | 1.000E+00 | 1.000E+00 | 1.000E+00 | 1.000E+00 |
| Kurtosis MYO ES | Shape | 0.038 | 0.027 | 0.011 | 0.158 | 1.000E+00 | 1.000E+00 | 1.000E+00 | 1.000E+00 |
| ClusterProminence glcm MYO ES | Shape | 0.025 | -0.017 | 0.002 | -0.147 | 1.000E+00 | 1.000E+00 | 1.000E+00 | 1.000E+00 |
| ClusterShade glcm MYO ES | Shape | 0.003 | -0.025 | -0.007 | -0.112 | 1.000E+00 | 1.000E+00 | 1.000E+00 | 1.000E+00 |
| SurfaceAreatoVolumeRatio RV ES | Shape | 0.055 | -0.026 | 0.020 | -0.078 | 1.000E+00 | 1.000E+00 | 1.000E+00 | 1.000E+00 |
| SurfaceAreatoVolumeRatio LV ED | Shape | -0.030 | -0.021 | 0.024 | -0.040 | 1.000E+00 | 1.000E+00 | 1.000E+00 | 1.000E+00 |
| InformalMeasureofCorrelation1 glcm MYO ES | Shape | 0.282 | 0.164 | 0.101 | 0.161 | 4.062E-06 | 1.284E-09 | 7.426E-03 | 1.000E+00 |
| SurfaceAreatoVolumeRatio RV ED | Shape | 0.168 | 0.023 | 0.046 | 0.008 | 1.168E-02 | 1.000E+00 | 1.000E+00 | 1.000E+00 |
| LeastAxis RV ED | Size | -0.092 | -0.002 | -0.025 | -0.041 | 1.000E+00 | 1.000E+00 | 1.000E+00 | 1.000E+00 |
| Volume RV ED | Size | -0.229 | -0.087 | -0.065 | -0.030 | 8.237E-08 | 1.088E-04 | 5.475E-02 | 1.000E+00 |
| Max2DdiameterColumn RV ED | Size | -0.122 | -0.025 | -0.013 | -0.038 | 9.953E-01 | 1.000E+00 | 1.000E+00 | 1.000E+00 |
| MinorAxis RV ED | Size | -0.225 | -0.075 | -0.090 | 0.062 | 2.121E-04 | 1.372E-01 | 1.233E-02 | 1.000E+00 |
| MinorAxis RV ES | Size | -0.214 | -0.087 | -0.096 | 0.061 | 4.662E-04 | 1.229E-02 | 2.359E-03 | 1.000E+00 |
| GrayLevelNonUniformity glszm MYO ED | Size | -0.149 | 0.001 | 0.019 | -0.039 | 9.203E-01 | 1.000E+00 | 1.000E+00 | 1.000E+00 |
| SizeZoneNonUniformity glszm MYO ED | Size | -0.149 | 0.001 | 0.019 | -0.039 | 9.203E-01 | 1.000E+00 | 1.000E+00 | 1.000E+00 |
| SurfaceArea RV ED | Size | -0.224 | -0.071 | -0.067 | -0.012 | 8.227E-07 | 1.907E-02 | 6.379E-02 | 1.000E+00 |
| Max2DdiameterColumn RV ES | Size | -0.119 | -0.023 | -0.033 | -0.047 | 1.000E+00 | 1.000E+00 | 1.000E+00 | 1.000E+00 |
| LeastAxis RV ES | Size | -0.081 | 0.033 | -0.023 | 0.003 | 1.000E+00 | 1.000E+00 | 1.000E+00 | 1.000E+00 |
| LeastAxis LV ED | Size | -0.163 | -0.078 | -0.112 | 0.049 | 3.639E-02 | 3.480E-02 | 1.936E-05 | 1.000E+00 |
| Volume RV ES | Size | -0.177 | -0.089 | -0.085 | 0.022 | 8.456E-04 | 2.020E-04 | 9.719E-04 | 1.000E+00 |
| MinorAxis LV ED | Size | -0.137 | -0.017 | -0.046 | -0.052 | 3.797E-01 | 1.000E+00 | 1.000E+00 | 1.000E+00 |
| Max2DdiameterSlice LV ED | Size | -0.181 | -0.008 | -0.029 | -0.049 | 3.554E-02 | 1.000E+00 | 1.000E+00 | 1.000E+00 |
| Max2DdiameterRow RV ED | Size | -0.126 | -0.037 | -0.049 | 0.031 | 1.000E+00 | 1.000E+00 | 1.000E+00 | 1.000E+00 |
| SurfaceArea RV ES | Size | -0.202 | -0.081 | -0.082 | 0.001 | 2.120E-05 | 1.430E-03 | 1.906E-03 | 1.000E+00 |
| Volume LV ED | Size | -0.140 | -0.070 | -0.048 | -0.010 | 6.291E-02 | 2.539E-02 | 1.000E+00 | 1.000E+00 |
| Max2DdiameterSlice LV ES | Size | -0.062 | -0.020 | -0.033 | -0.047 | 1.000E+00 | 1.000E+00 | 1.000E+00 | 1.000E+00 |
| Max3Ddiameter LV ED | Size | -0.088 | -0.031 | -0.004 | -0.062 | 1.000E+00 | 1.000E+00 | 1.000E+00 | 1.000E+00 |
| LeastAxis LV ES | Size | -0.105 | -0.020 | -0.057 | -0.010 | 1.000E+00 | 1.000E+00 | 1.000E+00 | 1.000E+00 |
| Max2DdiameterRow LV ED | Size | -0.082 | -0.023 | 0.006 | -0.063 | 1.000E+00 | 1.000E+00 | 1.000E+00 | 1.000E+00 |
| Max3Ddiameter LV ES | Size | -0.124 | -0.037 | -0.008 | -0.071 | 1.000E+00 | 1.000E+00 | 1.000E+00 | 1.000E+00 |
| Max3Ddiameter RV ED | Size | -0.156 | -0.049 | -0.027 | -0.028 | 4.452E-02 | 1.000E+00 | 1.000E+00 | 1.000E+00 |
| MinorAxis LV ES | Size | -0.100 | -0.011 | -0.034 | -0.054 | 1.000E+00 | 1.000E+00 | 1.000E+00 | 1.000E+00 |
| Max2DdiameterRow LV ES | Size | -0.150 | -0.032 | -0.013 | -0.067 | 1.010E-01 | 1.000E+00 | 1.000E+00 | 1.000E+00 |
| SurfaceArea LV ED | Size | -0.164 | -0.046 | -0.035 | -0.032 | 2.238E-03 | 1.000E+00 | 1.000E+00 | 1.000E+00 |
| MajorAxis LV ES | Size | -0.135 | -0.037 | -0.036 | -0.079 | 2.736E-01 | 1.000E+00 | 1.000E+00 | 1.000E+00 |
| Max2DdiameterColumn LV ES | Size | -0.116 | -0.045 | -0.029 | -0.081 | 1.000E+00 | 1.000E+00 | 1.000E+00 | 1.000E+00 |
| MajorAxis LV ED | Size | -0.085 | -0.004 | 0.016 | -0.069 | 1.000E+00 | 1.000E+00 | 1.000E+00 | 1.000E+00 |
| Max2DdiameterColumn LV ED | Size | -0.065 | -0.014 | 0.000 | -0.087 | 1.000E+00 | 1.000E+00 | 1.000E+00 | 1.000E+00 |
| Volume LV ES | Size | -0.105 | -0.070 | -0.056 | -0.018 | 1.000E+00 | 9.260E-02 | 1.000E+00 | 1.000E+00 |
| SurfaceArea LV ES | Size | -0.131 | -0.043 | -0.044 | -0.037 | 2.329E-01 | 1.000E+00 | 1.000E+00 | 1.000E+00 |
| Max2DdiameterSlice RV ED | Size | -0.194 | -0.056 | -0.080 | 0.038 | 1.666E-02 | 1.000E+00 | 1.690E-01 | 1.000E+00 |
| Max2DdiameterRow RV ES | Size | -0.142 | -0.056 | -0.060 | 0.036 | 8.485E-01 | 1.000E+00 | 1.000E+00 | 1.000E+00 |
| MajorAxis RV ED | Size | -0.098 | -0.014 | -0.020 | -0.066 | 1.000E+00 | 1.000E+00 | 1.000E+00 | 1.000E+00 |
| RunLengthNonUniformity glrlm MYO ED | Size | -0.147 | -0.002 | -0.047 | -0.024 | 7.780E-01 | 1.000E+00 | 1.000E+00 | 1.000E+00 |
| RunLengthNonUniformity glrlm MYO ES | Size | -0.115 | -0.031 | -0.056 | 0.006 | 1.000E+00 | 1.000E+00 | 1.000E+00 | 1.000E+00 |
| Max3Ddiameter RV ES | Size | -0.213 | -0.075 | -0.020 | -0.046 | 1.411E-04 | 4.908E-02 | 1.000E+00 | 1.000E+00 |
| DependenceNonUniformity gldm MYO ED | Size | -0.140 | -0.022 | 0.012 | -0.010 | 8.854E-01 | 1.000E+00 | 1.000E+00 | 1.000E+00 |
| Max2DdiameterSlice RV ES | Size | -0.203 | -0.113 | -0.081 | 0.057 | 8.168E-03 | 2.702E-04 | 1.354E-01 | 1.000E+00 |
| GrayLevelNonUniformity glszm MYO ES | Size | 0.063 | 0.076 | 0.048 | -0.022 | 1.000E+00 | 4.213E-01 | 1.000E+00 | 1.000E+00 |
| SizeZoneNonUniformity glszm MYO ES | Size | 0.063 | 0.076 | 0.048 | -0.022 | 1.000E+00 | 4.213E-01 | 1.000E+00 | 1.000E+00 |
| MajorAxis RV ES | Size | -0.178 | -0.082 | -0.043 | -0.079 | 1.791E-02 | 3.003E-02 | 1.000E+00 | 1.000E+00 |

**Supplementary Table 7. Associations of vascular with radiomics features from linear regression models (all results are for VRF *age in fully adjusted models)**

|  |  | **Beta** | | | | | **Adjusted p-value** | | | | |
| --- | --- | --- | --- | --- | --- | --- | --- | --- | --- | --- | --- |
| **Feature** | **Cluster** | **Diabetes** | **High chol.** | **Hypertension** | **Smoker** | **Diabetes** | | **High chol.** | **Hypertension** | **Smoker** |  |
| InterquartileRange MYO ES | Global Intensity | 0.062 | 0.047 | 0.060 | -0.019 | 1.000E+00 | | 1.181E-01 | 1.129E-03 | 1.000E+00 |  |
| RobustMeanAbsoluteDeviation MYO ES | Global Intensity | 0.063 | 0.043 | 0.057 | -0.023 | 1.000E+00 | | 2.825E-01 | 2.938E-03 | 1.000E+00 |  |
| Percentile90 MYO ES | Global Intensity | 0.003 | 0.038 | 0.032 | 0.006 | 1.000E+00 | | 6.960E-01 | 1.000E+00 | 1.000E+00 |  |
| RootMeanSquared MYO ES | Global Intensity | -0.011 | 0.040 | 0.025 | 0.018 | 1.000E+00 | | 3.754E-01 | 1.000E+00 | 1.000E+00 |  |
| MeanAbsoluteDeviation MYO ES | Global Intensity | 0.052 | 0.036 | 0.057 | -0.031 | 1.000E+00 | | 1.000E+00 | 2.827E-03 | 1.000E+00 |  |
| Mean MYO ES | Global Intensity | -0.014 | 0.040 | 0.023 | 0.021 | 1.000E+00 | | 3.851E-01 | 1.000E+00 | 1.000E+00 |  |
| Median MYO ES | Global Intensity | -0.012 | 0.042 | 0.023 | 0.029 | 1.000E+00 | | 2.012E-01 | 1.000E+00 | 1.000E+00 |  |
| Entropy MYO ES | Global Intensity | 0.045 | 0.035 | 0.052 | -0.019 | 1.000E+00 | | 1.000E+00 | 1.023E-02 | 1.000E+00 |  |
| SumEntropy glcm MYO ES | Global Intensity | 0.045 | 0.034 | 0.045 | -0.018 | 1.000E+00 | | 1.000E+00 | 1.377E-01 | 1.000E+00 |  |
| Percentile10 MYO ES | Global Intensity | -0.040 | 0.032 | 0.006 | 0.030 | 1.000E+00 | | 1.000E+00 | 1.000E+00 | 1.000E+00 |  |
| JointEntropy glcm MYO ES | Global Intensity | 0.036 | 0.030 | 0.049 | -0.010 | 1.000E+00 | | 1.000E+00 | 2.460E-02 | 1.000E+00 |  |
| Percentile10 MYO ED | Global Intensity | -0.065 | 0.024 | -0.016 | 0.008 | 1.000E+00 | | 1.000E+00 | 1.000E+00 | 1.000E+00 |  |
| Mean MYO ED | Global Intensity | -0.050 | 0.019 | -0.003 | 0.005 | 1.000E+00 | | 1.000E+00 | 1.000E+00 | 1.000E+00 |  |
| Percentile90 MYO ED | Global Intensity | -0.024 | 0.020 | 0.012 | 0.005 | 1.000E+00 | | 1.000E+00 | 1.000E+00 | 1.000E+00 |  |
| RootMeanSquared MYO ED | Global Intensity | -0.045 | 0.020 | 0.001 | 0.003 | 1.000E+00 | | 1.000E+00 | 1.000E+00 | 1.000E+00 |  |
| Maximum MYO ED | Global Intensity | -0.014 | 0.024 | 0.015 | -0.022 | 1.000E+00 | | 1.000E+00 | 1.000E+00 | 1.000E+00 |  |
| Median MYO ED | Global Intensity | -0.059 | 0.014 | -0.011 | 0.006 | 1.000E+00 | | 1.000E+00 | 1.000E+00 | 1.000E+00 |  |
| SmallAreaHighGrayLevelEmphasis glszm MYO ES | Global Intensity | 0.009 | 0.041 | 0.042 | -0.027 | 1.000E+00 | | 4.942E-01 | 3.159E-01 | 1.000E+00 |  |
| Variance MYO ES | Global Intensity | 0.037 | 0.025 | 0.047 | -0.040 | 1.000E+00 | | 1.000E+00 | 9.190E-02 | 1.000E+00 |  |
| GrayLevelVariance gldm MYO ES | Global Intensity | 0.040 | 0.026 | 0.046 | -0.036 | 1.000E+00 | | 1.000E+00 | 1.054E-01 | 1.000E+00 |  |
| GrayLevelVariance glrlm MYO ES | Global Intensity | 0.032 | 0.025 | 0.046 | -0.040 | 1.000E+00 | | 1.000E+00 | 1.135E-01 | 1.000E+00 |  |
| Energy MYO ES | Global Intensity | 0.001 | 0.046 | 0.004 | 0.009 | 1.000E+00 | | 6.681E-02 | 1.000E+00 | 1.000E+00 |  |
| ClusterTendency glcm MYO ES | Global Intensity | 0.047 | 0.025 | 0.038 | -0.039 | 1.000E+00 | | 1.000E+00 | 1.000E+00 | 1.000E+00 |  |
| SumofSquares glcm MYO ES | Global Intensity | 0.042 | 0.024 | 0.043 | -0.033 | 1.000E+00 | | 1.000E+00 | 2.558E-01 | 1.000E+00 |  |
| SmallDependenceHighGrayLevelEmphasis gldm MYO ES | Global Intensity | 0.004 | 0.032 | 0.049 | -0.035 | 1.000E+00 | | 1.000E+00 | 2.308E-02 | 1.000E+00 |  |
| Complexity ngtdm MYO ED | Global Intensity | -0.008 | 0.031 | 0.024 | -0.022 | 1.000E+00 | | 1.000E+00 | 1.000E+00 | 1.000E+00 |  |
| Range MYO ED | Global Intensity | -0.008 | 0.020 | 0.018 | -0.031 | 1.000E+00 | | 1.000E+00 | 1.000E+00 | 1.000E+00 |  |
| SmallDependenceHighGrayLevelEmphasis gldm MYO ED | Global Intensity | -0.018 | 0.007 | 0.022 | -0.024 | 1.000E+00 | | 1.000E+00 | 1.000E+00 | 1.000E+00 |  |
| Strength ngtdm MYO ED | Global Intensity | -0.004 | 0.020 | 0.010 | -0.030 | 1.000E+00 | | 1.000E+00 | 1.000E+00 | 1.000E+00 |  |
| HighGrayLevelZoneEmphasis glszm MYO ES | Global Intensity | 0.021 | 0.035 | 0.042 | -0.025 | 1.000E+00 | | 1.000E+00 | 3.211E-01 | 1.000E+00 |  |
| TotalEnergy MYO ES | Global Intensity | 0.002 | 0.042 | 0.000 | 0.014 | 1.000E+00 | | 1.940E-01 | 1.000E+00 | 1.000E+00 |  |
| ZoneEntropy glszm MYO ES | Global Intensity | 0.049 | 0.020 | 0.044 | -0.069 | 1.000E+00 | | 1.000E+00 | 1.966E-01 | 1.000E+00 |  |
| SmallAreaHighGrayLevelEmphasis glszm MYO ED | Global Intensity | -0.030 | 0.016 | 0.013 | -0.029 | 1.000E+00 | | 1.000E+00 | 1.000E+00 | 1.000E+00 |  |
| HighGrayLevelZoneEmphasis glszm MYO ED | Global Intensity | -0.019 | 0.017 | 0.014 | -0.022 | 1.000E+00 | | 1.000E+00 | 1.000E+00 | 1.000E+00 |  |
| Energy MYO ED | Global Intensity | -0.022 | 0.037 | -0.006 | 0.008 | 1.000E+00 | | 1.000E+00 | 1.000E+00 | 1.000E+00 |  |
| ShortRunHighGrayLevelEmphasis glrlm MYO ES | Global Intensity | 0.021 | 0.021 | 0.039 | -0.017 | 1.000E+00 | | 1.000E+00 | 6.387E-01 | 1.000E+00 |  |
| Autocorrelation glcm MYO ES | Global Intensity | 0.021 | 0.019 | 0.027 | -0.001 | 1.000E+00 | | 1.000E+00 | 1.000E+00 | 1.000E+00 |  |
| SumAverage glcm MYO ES | Global Intensity | 0.015 | 0.020 | 0.025 | -0.006 | 1.000E+00 | | 1.000E+00 | 1.000E+00 | 1.000E+00 |  |
| JointAverage glcm MYO ES | Global Intensity | 0.015 | 0.020 | 0.025 | -0.006 | 1.000E+00 | | 1.000E+00 | 1.000E+00 | 1.000E+00 |  |
| HighGrayLevelEmphasis gldm MYO ES | Global Intensity | 0.021 | 0.019 | 0.028 | -0.005 | 1.000E+00 | | 1.000E+00 | 1.000E+00 | 1.000E+00 |  |
| HighGrayLevelRunEmphasis glrlm MYO ES | Global Intensity | 0.021 | 0.020 | 0.029 | -0.010 | 1.000E+00 | | 1.000E+00 | 1.000E+00 | 1.000E+00 |  |
| TotalEnergy MYO ED | Global Intensity | -0.017 | 0.032 | -0.009 | 0.013 | 1.000E+00 | | 1.000E+00 | 1.000E+00 | 1.000E+00 |  |
| ShortRunHighGrayLevelEmphasis glrlm MYO ED | Global Intensity | -0.009 | 0.004 | 0.011 | -0.024 | 1.000E+00 | | 1.000E+00 | 1.000E+00 | 1.000E+00 |  |
| RunEntropy glrlm MYO ED | Global Intensity | -0.006 | 0.009 | -0.026 | 0.021 | 1.000E+00 | | 1.000E+00 | 1.000E+00 | 1.000E+00 |  |
| HighGrayLevelRunEmphasis glrlm MYO ED | Global Intensity | -0.011 | 0.003 | 0.005 | -0.024 | 1.000E+00 | | 1.000E+00 | 1.000E+00 | 1.000E+00 |  |
| HighGrayLevelEmphasis gldm MYO ED | Global Intensity | -0.012 | 0.000 | 0.002 | -0.024 | 1.000E+00 | | 1.000E+00 | 1.000E+00 | 1.000E+00 |  |
| InformalMeasureofCorrelation2 glcm MYO ED | Global Intensity | 0.004 | -0.011 | -0.005 | -0.016 | 1.000E+00 | | 1.000E+00 | 1.000E+00 | 1.000E+00 |  |
| DependenceEntropy gldm MYO ED | Global Intensity | -0.009 | 0.001 | -0.022 | -0.013 | 1.000E+00 | | 1.000E+00 | 1.000E+00 | 1.000E+00 |  |
| JointAverage glcm MYO ED | Global Intensity | -0.013 | -0.002 | 0.000 | -0.024 | 1.000E+00 | | 1.000E+00 | 1.000E+00 | 1.000E+00 |  |
| SumAverage glcm MYO ED | Global Intensity | -0.013 | -0.002 | 0.000 | -0.024 | 1.000E+00 | | 1.000E+00 | 1.000E+00 | 1.000E+00 |  |
| Autocorrelation glcm MYO ED | Global Intensity | -0.012 | -0.004 | -0.001 | -0.020 | 1.000E+00 | | 1.000E+00 | 1.000E+00 | 1.000E+00 |  |
| Correlation glcm MYO ED | Global Intensity | 0.020 | -0.007 | -0.006 | -0.010 | 1.000E+00 | | 1.000E+00 | 1.000E+00 | 1.000E+00 |  |
| ZonePercentage glszm MYO ED | Global Variance | -0.005 | 0.005 | 0.040 | -0.008 | 1.000E+00 | | 1.000E+00 | 2.726E-02 | 1.000E+00 |  |
| DifferenceVariance glcm MYO ED | Global Variance | 0.012 | 0.028 | 0.041 | -0.005 | 1.000E+00 | | 1.000E+00 | 6.314E-02 | 1.000E+00 |  |
| SmallDependenceEmphasis gldm MYO ED | Global Variance | -0.008 | 0.006 | 0.039 | -0.009 | 1.000E+00 | | 1.000E+00 | 2.722E-02 | 1.000E+00 |  |
| DifferenceEntropy glcm MYO ED | Global Variance | 0.004 | 0.020 | 0.039 | 0.003 | 1.000E+00 | | 1.000E+00 | 5.111E-02 | 1.000E+00 |  |
| Contrast glcm MYO ED | Global Variance | 0.008 | 0.022 | 0.036 | -0.001 | 1.000E+00 | | 1.000E+00 | 2.357E-01 | 1.000E+00 |  |
| SmallDependenceEmphasis gldm MYO ES | Global Variance | -0.010 | 0.020 | 0.039 | -0.024 | 1.000E+00 | | 1.000E+00 | 2.163E-01 | 1.000E+00 |  |
| ZonePercentage glszm MYO ES | Global Variance | -0.016 | 0.019 | 0.035 | -0.028 | 1.000E+00 | | 1.000E+00 | 7.419E-01 | 1.000E+00 |  |
| ShortRunEmphasis glrlm MYO ED | Global Variance | 0.006 | 0.008 | 0.041 | -0.011 | 1.000E+00 | | 1.000E+00 | 2.238E-02 | 1.000E+00 |  |
| DifferenceEntropy glcm MYO ES | Global Variance | 0.010 | 0.018 | 0.045 | -0.004 | 1.000E+00 | | 1.000E+00 | 4.803E-02 | 1.000E+00 |  |
| RunLengthNonUniformityNormalized glrlm MYO ED | Global Variance | 0.008 | 0.008 | 0.042 | -0.011 | 1.000E+00 | | 1.000E+00 | 1.745E-02 | 1.000E+00 |  |
| RunPercentage glrlm MYO ES | Global Variance | 0.018 | 0.011 | 0.042 | -0.006 | 1.000E+00 | | 1.000E+00 | 8.782E-02 | 1.000E+00 |  |
| DifferenceAverage glcm MYO ED | Global Variance | 0.004 | 0.015 | 0.034 | 0.003 | 1.000E+00 | | 1.000E+00 | 3.042E-01 | 1.000E+00 |  |
| Contrast ngtdm MYO ES | Global Variance | 0.020 | 0.001 | 0.022 | 0.037 | 1.000E+00 | | 1.000E+00 | 1.000E+00 | 1.000E+00 |  |
| RunPercentage glrlm MYO ED | Global Variance | 0.004 | 0.005 | 0.039 | -0.015 | 1.000E+00 | | 1.000E+00 | 5.428E-02 | 1.000E+00 |  |
| Contrast ngtdm MYO ED | Global Variance | 0.017 | 0.005 | 0.026 | 0.019 | 1.000E+00 | | 1.000E+00 | 1.000E+00 | 1.000E+00 |  |
| DependenceNonUniformityNormalized gldm MYO ES | Global Variance | 0.038 | 0.011 | 0.043 | 0.034 | 1.000E+00 | | 1.000E+00 | 1.696E-01 | 1.000E+00 |  |
| DifferenceVariance glcm MYO ES | Global Variance | 0.011 | 0.019 | 0.044 | -0.027 | 1.000E+00 | | 1.000E+00 | 1.193E-01 | 1.000E+00 |  |
| MeanAbsoluteDeviation MYO ED | Global Variance | 0.007 | 0.015 | 0.027 | -0.002 | 1.000E+00 | | 1.000E+00 | 1.000E+00 | 1.000E+00 |  |
| DifferenceAverage glcm MYO ES | Global Variance | 0.020 | 0.017 | 0.047 | 0.006 | 1.000E+00 | | 1.000E+00 | 3.167E-02 | 1.000E+00 |  |
| InverseVariance glcm MYO ES | Global Variance | 0.021 | 0.015 | 0.036 | 0.019 | 1.000E+00 | | 1.000E+00 | 8.363E-01 | 1.000E+00 |  |
| GrayLevelVariance glrlm MYO ED | Global Variance | 0.004 | 0.020 | 0.022 | -0.019 | 1.000E+00 | | 1.000E+00 | 1.000E+00 | 1.000E+00 |  |
| Entropy MYO ED | Global Variance | 0.000 | 0.009 | 0.023 | -0.001 | 1.000E+00 | | 1.000E+00 | 1.000E+00 | 1.000E+00 |  |
| ShortRunEmphasis glrlm MYO ES | Global Variance | 0.010 | 0.009 | 0.039 | -0.009 | 1.000E+00 | | 1.000E+00 | 2.558E-01 | 1.000E+00 |  |
| Contrast glcm MYO ES | Global Variance | 0.018 | 0.018 | 0.047 | -0.011 | 1.000E+00 | | 1.000E+00 | 4.760E-02 | 1.000E+00 |  |
| Variance MYO ED | Global Variance | 0.006 | 0.018 | 0.024 | -0.019 | 1.000E+00 | | 1.000E+00 | 1.000E+00 | 1.000E+00 |  |
| GrayLevelVariance gldm MYO ED | Global Variance | 0.004 | 0.019 | 0.024 | -0.019 | 1.000E+00 | | 1.000E+00 | 1.000E+00 | 1.000E+00 |  |
| JointEntropy glcm MYO ED | Global Variance | 0.002 | 0.008 | 0.028 | 0.000 | 1.000E+00 | | 1.000E+00 | 1.000E+00 | 1.000E+00 |  |
| RunLengthNonUniformityNormalized glrlm MYO ES | Global Variance | 0.016 | 0.009 | 0.043 | -0.008 | 1.000E+00 | | 1.000E+00 | 8.520E-02 | 1.000E+00 |  |
| SumofSquares glcm MYO ED | Global Variance | 0.009 | 0.019 | 0.030 | -0.012 | 1.000E+00 | | 1.000E+00 | 1.000E+00 | 1.000E+00 |  |
| DependenceNonUniformityNormalized gldm MYO ED | Global Variance | 0.009 | 0.004 | 0.029 | -0.002 | 1.000E+00 | | 1.000E+00 | 1.000E+00 | 1.000E+00 |  |
| RobustMeanAbsoluteDeviation MYO ED | Global Variance | 0.003 | 0.008 | 0.022 | 0.005 | 1.000E+00 | | 1.000E+00 | 1.000E+00 | 1.000E+00 |  |
| SumEntropy glcm MYO ED | Global Variance | 0.004 | 0.007 | 0.027 | -0.002 | 1.000E+00 | | 1.000E+00 | 1.000E+00 | 1.000E+00 |  |
| ZoneEntropy glszm MYO ED | Global Variance | 0.032 | 0.026 | 0.019 | -0.008 | 1.000E+00 | | 1.000E+00 | 1.000E+00 | 1.000E+00 |  |
| InterquartileRange MYO ED | Global Variance | -0.002 | 0.006 | 0.018 | 0.006 | 1.000E+00 | | 1.000E+00 | 1.000E+00 | 1.000E+00 |  |
| ClusterTendency glcm MYO ED | Global Variance | 0.009 | 0.017 | 0.026 | -0.017 | 1.000E+00 | | 1.000E+00 | 1.000E+00 | 1.000E+00 |  |
| InverseVariance glcm MYO ED | Global Variance | -0.003 | 0.000 | 0.020 | -0.003 | 1.000E+00 | | 1.000E+00 | 1.000E+00 | 1.000E+00 |  |
| GrayLevelVariance glszm MYO ED | Global Variance | 0.003 | 0.020 | 0.015 | -0.029 | 1.000E+00 | | 1.000E+00 | 1.000E+00 | 1.000E+00 |  |
| SmallDependenceLowGrayLevelEmphasis gldm MYO ED | Local Dimness | 0.021 | -0.001 | 0.019 | 0.029 | 1.000E+00 | | 1.000E+00 | 1.000E+00 | 1.000E+00 |  |
| ShortRunLowGrayLevelEmphasis glrlm MYO ED | Local Dimness | 0.022 | -0.007 | 0.001 | 0.041 | 1.000E+00 | | 1.000E+00 | 1.000E+00 | 1.000E+00 |  |
| LowGrayLevelEmphasis gldm MYO ED | Local Dimness | 0.022 | -0.005 | 0.000 | 0.045 | 1.000E+00 | | 1.000E+00 | 1.000E+00 | 1.000E+00 |  |
| LowGrayLevelRunEmphasis glrlm MYO ED | Local Dimness | 0.021 | -0.008 | -0.004 | 0.044 | 1.000E+00 | | 1.000E+00 | 1.000E+00 | 1.000E+00 |  |
| SmallDependenceLowGrayLevelEmphasis gldm MYO ES | Local Dimness | 0.008 | -0.013 | -0.002 | 0.025 | 1.000E+00 | | 1.000E+00 | 1.000E+00 | 1.000E+00 |  |
| ShortRunLowGrayLevelEmphasis glrlm MYO ES | Local Dimness | 0.006 | -0.016 | -0.015 | 0.036 | 1.000E+00 | | 1.000E+00 | 1.000E+00 | 1.000E+00 |  |
| LargeDependenceLowGrayLevelEmphasis gldm MYO ED | Local Dimness | 0.012 | -0.006 | -0.018 | 0.052 | 1.000E+00 | | 1.000E+00 | 1.000E+00 | 1.000E+00 |  |
| LowGrayLevelEmphasis gldm MYO ES | Local Dimness | 0.002 | -0.019 | -0.019 | 0.040 | 1.000E+00 | | 1.000E+00 | 1.000E+00 | 1.000E+00 |  |
| LowGrayLevelRunEmphasis glrlm MYO ES | Local Dimness | 0.002 | -0.017 | -0.018 | 0.040 | 1.000E+00 | | 1.000E+00 | 1.000E+00 | 1.000E+00 |  |
| LowGrayLevelZoneEmphasis glszm MYO ED | Local Dimness | 0.024 | -0.011 | -0.014 | 0.021 | 1.000E+00 | | 1.000E+00 | 1.000E+00 | 1.000E+00 |  |
| SmallAreaLowGrayLevelEmphasis glszm MYO ED | Local Dimness | 0.009 | -0.013 | -0.015 | 0.022 | 1.000E+00 | | 1.000E+00 | 1.000E+00 | 1.000E+00 |  |
| LongRunLowGrayLevelEmphasis glrlm MYO ED | Local Dimness | 0.016 | -0.008 | -0.014 | 0.054 | 1.000E+00 | | 1.000E+00 | 1.000E+00 | 1.000E+00 |  |
| SmallAreaLowGrayLevelEmphasis glszm MYO ES | Local Dimness | 0.013 | -0.014 | -0.012 | 0.045 | 1.000E+00 | | 1.000E+00 | 1.000E+00 | 1.000E+00 |  |
| LowGrayLevelZoneEmphasis glszm MYO ES | Local Dimness | 0.005 | -0.020 | -0.011 | 0.035 | 1.000E+00 | | 1.000E+00 | 1.000E+00 | 1.000E+00 |  |
| Busyness ngtdm MYO ED | Local Dimness | 0.024 | 0.003 | -0.003 | 0.073 | 1.000E+00 | | 1.000E+00 | 1.000E+00 | 1.000E+00 |  |
| LargeDependenceLowGrayLevelEmphasis gldm MYO ES | Local Dimness | -0.008 | -0.019 | -0.023 | 0.040 | 1.000E+00 | | 1.000E+00 | 1.000E+00 | 1.000E+00 |  |
| Busyness ngtdm MYO ES | Local Dimness | -0.021 | -0.018 | -0.027 | 0.048 | 1.000E+00 | | 1.000E+00 | 1.000E+00 | 1.000E+00 |  |
| LongRunLowGrayLevelEmphasis glrlm MYO ES | Local Dimness | 0.007 | -0.015 | -0.013 | 0.045 | 1.000E+00 | | 1.000E+00 | 1.000E+00 | 1.000E+00 |  |
| LargeAreaLowGrayLevelEmphasis glszm MYO ED | Local Dimness | -0.009 | -0.007 | -0.009 | 0.079 | 1.000E+00 | | 1.000E+00 | 1.000E+00 | 1.000E+00 |  |
| LargeAreaLowGrayLevelEmphasis glszm MYO ES | Local Dimness | -0.008 | -0.011 | -0.011 | 0.061 | 1.000E+00 | | 1.000E+00 | 1.000E+00 | 1.000E+00 |  |
| InformalMeasureofCorrelation2 glcm MYO ES | Local Uniformity | 0.053 | 0.031 | 0.014 | -0.042 | 1.000E+00 | | 1.000E+00 | 1.000E+00 | 1.000E+00 |  |
| DependenceEntropy gldm MYO ES | Local Uniformity | 0.037 | 0.035 | 0.022 | -0.044 | 1.000E+00 | | 1.000E+00 | 1.000E+00 | 1.000E+00 |  |
| Correlation glcm MYO ES | Local Uniformity | 0.053 | 0.023 | 0.006 | -0.033 | 1.000E+00 | | 1.000E+00 | 1.000E+00 | 1.000E+00 |  |
| RunEntropy glrlm MYO ES | Local Uniformity | 0.021 | 0.024 | 0.006 | -0.020 | 1.000E+00 | | 1.000E+00 | 1.000E+00 | 1.000E+00 |  |
| InverseDifferenceMomentNormalized glcm MYO ES | Local Uniformity | 0.008 | 0.020 | -0.002 | -0.061 | 1.000E+00 | | 1.000E+00 | 1.000E+00 | 1.000E+00 |  |
| GrayLevelNonUniformityNormalized glszm MYO ED | Local Uniformity | -0.013 | -0.020 | -0.022 | 0.039 | 1.000E+00 | | 1.000E+00 | 1.000E+00 | 1.000E+00 |  |
| MaximumProbability glcm MYO ED | Local Uniformity | 0.005 | -0.008 | -0.021 | 0.000 | 1.000E+00 | | 1.000E+00 | 1.000E+00 | 1.000E+00 |  |
| InverseDifferenceNormalized glcm MYO ES | Local Uniformity | -0.004 | 0.007 | -0.020 | -0.053 | 1.000E+00 | | 1.000E+00 | 1.000E+00 | 1.000E+00 |  |
| JointEnergy glcm MYO ED | Local Uniformity | 0.002 | -0.004 | -0.022 | 0.003 | 1.000E+00 | | 1.000E+00 | 1.000E+00 | 1.000E+00 |  |
| LargeDependenceHighGrayLevelEmphasis gldm MYO ES | Local Uniformity | 0.006 | 0.009 | -0.011 | 0.007 | 1.000E+00 | | 1.000E+00 | 1.000E+00 | 1.000E+00 |  |
| DependenceVariance gldm MYO ED | Local Uniformity | -0.002 | 0.000 | -0.036 | 0.016 | 1.000E+00 | | 1.000E+00 | 3.010E-01 | 1.000E+00 |  |
| Uniformity MYO ED | Local Uniformity | 0.001 | -0.007 | -0.022 | -0.003 | 1.000E+00 | | 1.000E+00 | 1.000E+00 | 1.000E+00 |  |
| DependenceNonUniformity gldm MYO ES | Local Uniformity | 0.026 | 0.021 | -0.004 | 0.001 | 1.000E+00 | | 1.000E+00 | 1.000E+00 | 1.000E+00 |  |
| InverseDifferenceNormalized glcm MYO ED | Local Uniformity | -0.012 | 0.002 | -0.026 | -0.032 | 1.000E+00 | | 1.000E+00 | 1.000E+00 | 1.000E+00 |  |
| GrayLevelNonUniformityNormalized glrlm MYO ED | Local Uniformity | 0.002 | -0.009 | -0.017 | -0.012 | 1.000E+00 | | 1.000E+00 | 1.000E+00 | 1.000E+00 |  |
| GrayLevelNonUniformity gldm MYO ED | Local Uniformity | 0.019 | 0.006 | -0.024 | -0.006 | 1.000E+00 | | 1.000E+00 | 1.000E+00 | 1.000E+00 |  |
| LongRunHighGrayLevelEmphasis glrlm MYO ES | Local Uniformity | 0.014 | 0.002 | -0.014 | -0.002 | 1.000E+00 | | 1.000E+00 | 1.000E+00 | 1.000E+00 |  |
| InverseDifference glcm MYO ED | Local Uniformity | -0.002 | -0.011 | -0.031 | -0.003 | 1.000E+00 | | 1.000E+00 | 8.118E-01 | 1.000E+00 |  |
| InverseDifference glcm MYO ES | Local Uniformity | -0.020 | -0.016 | -0.044 | -0.012 | 1.000E+00 | | 1.000E+00 | 7.212E-02 | 1.000E+00 |  |
| InverseDifferenceMoment glcm MYO ES | Local Uniformity | -0.020 | -0.016 | -0.045 | -0.011 | 1.000E+00 | | 1.000E+00 | 5.176E-02 | 1.000E+00 |  |
| LargeDependenceEmphasis gldm MYO ED | Local Uniformity | -0.004 | -0.003 | -0.040 | 0.019 | 1.000E+00 | | 1.000E+00 | 4.888E-02 | 1.000E+00 |  |
| InverseDifferenceMoment glcm MYO ED | Local Uniformity | -0.002 | -0.012 | -0.032 | -0.004 | 1.000E+00 | | 1.000E+00 | 6.088E-01 | 1.000E+00 |  |
| GrayLevelNonUniformity glrlm MYO ED | Local Uniformity | 0.025 | 0.011 | -0.012 | -0.022 | 1.000E+00 | | 1.000E+00 | 1.000E+00 | 1.000E+00 |  |
| InverseDifferenceMomentNormalized glcm MYO ED | Local Uniformity | -0.023 | -0.001 | -0.026 | -0.047 | 1.000E+00 | | 1.000E+00 | 1.000E+00 | 1.000E+00 |  |
| RunVariance glrlm MYO ED | Local Uniformity | 0.007 | -0.003 | -0.035 | 0.021 | 1.000E+00 | | 1.000E+00 | 2.560E-01 | 1.000E+00 |  |
| LongRunEmphasis glrlm MYO ED | Local Uniformity | 0.005 | -0.004 | -0.036 | 0.021 | 1.000E+00 | | 1.000E+00 | 1.797E-01 | 1.000E+00 |  |
| LargeDependenceEmphasis gldm MYO ES | Local Uniformity | -0.019 | -0.010 | -0.041 | 0.007 | 1.000E+00 | | 1.000E+00 | 1.371E-01 | 1.000E+00 |  |
| ZoneVariance glszm MYO ED | Local Uniformity | 0.009 | -0.003 | -0.030 | 0.024 | 1.000E+00 | | 1.000E+00 | 9.751E-01 | 1.000E+00 |  |
| LargeAreaEmphasis glszm MYO ED | Local Uniformity | 0.008 | -0.003 | -0.030 | 0.025 | 1.000E+00 | | 1.000E+00 | 1.000E+00 | 1.000E+00 |  |
| DependenceVariance gldm MYO ES | Local Uniformity | -0.029 | -0.011 | -0.038 | -0.024 | 1.000E+00 | | 1.000E+00 | 5.678E-01 | 1.000E+00 |  |
| LongRunEmphasis glrlm MYO ES | Local Uniformity | 0.004 | -0.012 | -0.027 | 0.003 | 1.000E+00 | | 1.000E+00 | 1.000E+00 | 1.000E+00 |  |
| RunVariance glrlm MYO ES | Local Uniformity | 0.003 | -0.013 | -0.030 | 0.000 | 1.000E+00 | | 1.000E+00 | 1.000E+00 | 1.000E+00 |  |
| LargeAreaHighGrayLevelEmphasis glszm MYO ES | Local Uniformity | 0.009 | -0.004 | -0.038 | 0.021 | 1.000E+00 | | 1.000E+00 | 2.484E-01 | 1.000E+00 |  |
| LargeAreaHighGrayLevelEmphasis glszm MYO ED | Local Uniformity | 0.007 | 0.003 | -0.034 | 0.012 | 1.000E+00 | | 1.000E+00 | 3.923E-01 | 1.000E+00 |  |
| MaximumProbability glcm MYO ES | Local Uniformity | -0.035 | -0.027 | -0.033 | 0.002 | 1.000E+00 | | 1.000E+00 | 1.000E+00 | 1.000E+00 |  |
| LargeDependenceHighGrayLevelEmphasis gldm MYO ED | Local Uniformity | -0.018 | -0.004 | -0.036 | -0.002 | 1.000E+00 | | 1.000E+00 | 7.471E-01 | 1.000E+00 |  |
| LargeAreaEmphasis glszm MYO ES | Local Uniformity | -0.002 | -0.009 | -0.037 | 0.030 | 1.000E+00 | | 1.000E+00 | 1.899E-01 | 1.000E+00 |  |
| JointEnergy glcm MYO ES | Local Uniformity | -0.038 | -0.029 | -0.037 | 0.009 | 1.000E+00 | | 1.000E+00 | 9.654E-01 | 1.000E+00 |  |
| ZoneVariance glszm MYO ES | Local Uniformity | -0.003 | -0.010 | -0.037 | 0.029 | 1.000E+00 | | 1.000E+00 | 1.541E-01 | 1.000E+00 |  |
| LongRunHighGrayLevelEmphasis glrlm MYO ED | Local Uniformity | -0.011 | -0.001 | -0.031 | -0.006 | 1.000E+00 | | 1.000E+00 | 1.000E+00 | 1.000E+00 |  |
| GrayLevelNonUniformity glrlm MYO ES | Local Uniformity | -0.015 | -0.002 | -0.032 | -0.016 | 1.000E+00 | | 1.000E+00 | 1.383E-01 | 1.000E+00 |  |
| GrayLevelNonUniformity gldm MYO ES | Local Uniformity | -0.024 | -0.010 | -0.040 | 0.000 | 1.000E+00 | | 1.000E+00 | 8.685E-03 | 1.000E+00 |  |
| GrayLevelNonUniformityNormalized glszm MYO ES | Local Uniformity | -0.045 | -0.046 | -0.037 | 0.064 | 1.000E+00 | | 1.327E-01 | 1.000E+00 | 1.000E+00 |  |
| Uniformity MYO ES | Local Uniformity | -0.050 | -0.036 | -0.044 | 0.016 | 1.000E+00 | | 1.000E+00 | 1.391E-01 | 1.000E+00 |  |
| GrayLevelNonUniformityNormalized glrlm MYO ES | Local Uniformity | -0.043 | -0.036 | -0.047 | 0.014 | 1.000E+00 | | 1.000E+00 | 6.335E-02 | 1.000E+00 |  |
| Flatness RV ES | Shape | 0.050 | 0.008 | 0.010 | -0.017 | 1.000E+00 | | 1.000E+00 | 1.000E+00 | 1.000E+00 |  |
| Elongation RV ES | Shape | 0.016 | 0.019 | 0.005 | 0.046 | 1.000E+00 | | 1.000E+00 | 1.000E+00 | 1.000E+00 |  |
| Minimum MYO ED | Shape | -0.038 | 0.027 | -0.006 | 0.036 | 1.000E+00 | | 1.000E+00 | 1.000E+00 | 1.000E+00 |  |
| Elongation RV ED | Shape | 0.008 | 0.029 | 0.002 | 0.026 | 1.000E+00 | | 1.000E+00 | 1.000E+00 | 1.000E+00 |  |
| Flatness RV ED | Shape | 0.055 | -0.003 | -0.009 | -0.014 | 1.000E+00 | | 1.000E+00 | 1.000E+00 | 1.000E+00 |  |
| InformalMeasureofCorrelation1 glcm MYO ED | Shape | -0.015 | 0.018 | 0.023 | 0.003 | 1.000E+00 | | 1.000E+00 | 1.000E+00 | 1.000E+00 |  |
| Minimum MYO ES | Shape | -0.023 | 0.025 | -0.005 | 0.026 | 1.000E+00 | | 1.000E+00 | 1.000E+00 | 1.000E+00 |  |
| Maximum MYO ES | Shape | 0.004 | 0.037 | 0.028 | -0.041 | 1.000E+00 | | 1.000E+00 | 1.000E+00 | 1.000E+00 |  |
| Sphericity RV ED | Shape | 0.026 | -0.015 | -0.035 | -0.014 | 1.000E+00 | | 1.000E+00 | 1.000E+00 | 1.000E+00 |  |
| Sphericity LV ED | Shape | 0.008 | 0.025 | 0.001 | 0.031 | 1.000E+00 | | 1.000E+00 | 1.000E+00 | 1.000E+00 |  |
| Skewness MYO ED | Shape | 0.018 | 0.034 | 0.032 | -0.026 | 1.000E+00 | | 1.000E+00 | 1.000E+00 | 1.000E+00 |  |
| Sphericity RV ES | Shape | 0.014 | 0.000 | -0.008 | -0.014 | 1.000E+00 | | 1.000E+00 | 1.000E+00 | 1.000E+00 |  |
| GrayLevelVariance glszm MYO ES | Shape | 0.017 | 0.034 | 0.046 | -0.019 | 1.000E+00 | | 1.000E+00 | 7.856E-02 | 1.000E+00 |  |
| Range MYO ES | Shape | 0.011 | 0.032 | 0.032 | -0.053 | 1.000E+00 | | 1.000E+00 | 1.000E+00 | 1.000E+00 |  |
| SmallAreaEmphasis glszm MYO ES | Shape | -0.039 | 0.027 | -0.001 | 0.005 | 1.000E+00 | | 1.000E+00 | 1.000E+00 | 1.000E+00 |  |
| Strength ngtdm MYO ES | Shape | 0.006 | 0.023 | 0.018 | -0.051 | 1.000E+00 | | 1.000E+00 | 1.000E+00 | 1.000E+00 |  |
| SizeZoneNonUniformityNormalized glszm MYO ES | Shape | -0.033 | 0.026 | -0.003 | 0.011 | 1.000E+00 | | 1.000E+00 | 1.000E+00 | 1.000E+00 |  |
| Complexity ngtdm MYO ES | Shape | 0.009 | 0.032 | 0.038 | -0.045 | 1.000E+00 | | 1.000E+00 | 7.916E-01 | 1.000E+00 |  |
| Coarseness ngtdm MYO ES | Shape | 0.012 | -0.018 | 0.011 | 0.002 | 1.000E+00 | | 1.000E+00 | 1.000E+00 | 1.000E+00 |  |
| Flatness LV ED | Shape | 0.019 | -0.001 | -0.003 | -0.022 | 1.000E+00 | | 1.000E+00 | 1.000E+00 | 1.000E+00 |  |
| Elongation LV ED | Shape | 0.032 | 0.033 | 0.029 | -0.026 | 1.000E+00 | | 1.000E+00 | 1.000E+00 | 1.000E+00 |  |
| Sphericity LV ES | Shape | 0.004 | 0.028 | 0.019 | 0.025 | 1.000E+00 | | 1.000E+00 | 1.000E+00 | 1.000E+00 |  |
| ClusterShade glcm MYO ED | Shape | 0.024 | 0.033 | 0.027 | -0.039 | 1.000E+00 | | 1.000E+00 | 1.000E+00 | 1.000E+00 |  |
| Kurtosis MYO ED | Shape | 0.005 | 0.016 | 0.007 | -0.042 | 1.000E+00 | | 1.000E+00 | 1.000E+00 | 1.000E+00 |  |
| Coarseness ngtdm MYO ED | Shape | -0.013 | -0.035 | -0.017 | 0.009 | 1.000E+00 | | 1.826E-01 | 1.000E+00 | 1.000E+00 |  |
| Flatness LV ES | Shape | 0.012 | 0.000 | 0.004 | -0.017 | 1.000E+00 | | 1.000E+00 | 1.000E+00 | 1.000E+00 |  |
| Skewness MYO ES | Shape | -0.020 | -0.012 | -0.002 | -0.057 | 1.000E+00 | | 1.000E+00 | 1.000E+00 | 1.000E+00 |  |
| Elongation LV ES | Shape | 0.022 | 0.010 | 0.019 | -0.038 | 1.000E+00 | | 1.000E+00 | 1.000E+00 | 1.000E+00 |  |
| SmallAreaEmphasis glszm MYO ED | Shape | -0.042 | 0.000 | 0.002 | -0.030 | 1.000E+00 | | 1.000E+00 | 1.000E+00 | 1.000E+00 |  |
| SizeZoneNonUniformityNormalized glszm MYO ED | Shape | -0.043 | 0.000 | 0.004 | -0.032 | 1.000E+00 | | 1.000E+00 | 1.000E+00 | 1.000E+00 |  |
| ClusterProminence glcm MYO ED | Shape | 0.007 | 0.018 | 0.010 | -0.039 | 1.000E+00 | | 1.000E+00 | 1.000E+00 | 1.000E+00 |  |
| SurfaceAreatoVolumeRatio LV ES | Shape | 0.008 | -0.033 | -0.013 | 0.032 | 1.000E+00 | | 1.000E+00 | 1.000E+00 | 1.000E+00 |  |
| Kurtosis MYO ES | Shape | -0.038 | -0.004 | -0.013 | -0.036 | 1.000E+00 | | 1.000E+00 | 1.000E+00 | 1.000E+00 |  |
| ClusterProminence glcm MYO ES | Shape | 0.008 | -0.007 | -0.005 | -0.045 | 1.000E+00 | | 1.000E+00 | 1.000E+00 | 1.000E+00 |  |
| ClusterShade glcm MYO ES | Shape | 0.005 | -0.009 | -0.004 | -0.052 | 1.000E+00 | | 1.000E+00 | 1.000E+00 | 1.000E+00 |  |
| SurfaceAreatoVolumeRatio RV ES | Shape | -0.019 | -0.030 | -0.016 | 0.004 | 1.000E+00 | | 1.000E+00 | 1.000E+00 | 1.000E+00 |  |
| SurfaceAreatoVolumeRatio LV ED | Shape | -0.022 | -0.037 | -0.018 | 0.014 | 1.000E+00 | | 3.750E-01 | 1.000E+00 | 1.000E+00 |  |
| InformalMeasureofCorrelation1 glcm MYO ES | Shape | -0.056 | -0.024 | -0.007 | 0.044 | 1.000E+00 | | 1.000E+00 | 1.000E+00 | 1.000E+00 |  |
| SurfaceAreatoVolumeRatio RV ED | Shape | -0.042 | -0.021 | -0.002 | 0.016 | 1.000E+00 | | 1.000E+00 | 1.000E+00 | 1.000E+00 |  |
| LeastAxis RV ED | Size | 0.064 | 0.021 | 0.017 | -0.020 | 3.480E-01 | | 1.000E+00 | 1.000E+00 | 1.000E+00 |  |
| Volume RV ED | Size | 0.045 | 0.037 | 0.018 | -0.007 | 1.000E+00 | | 1.313E-02 | 1.000E+00 | 1.000E+00 |  |
| Max2DdiameterColumn RV ED | Size | 0.025 | 0.024 | 0.014 | -0.013 | 1.000E+00 | | 1.000E+00 | 1.000E+00 | 1.000E+00 |  |
| MinorAxis RV ED | Size | 0.019 | 0.051 | 0.027 | 0.017 | 1.000E+00 | | 1.579E-03 | 1.000E+00 | 1.000E+00 |  |
| MinorAxis RV ES | Size | 0.012 | 0.036 | 0.014 | 0.040 | 1.000E+00 | | 2.992E-01 | 1.000E+00 | 1.000E+00 |  |
| GrayLevelNonUniformity glszm MYO ED | Size | 0.015 | 0.023 | 0.034 | -0.010 | 1.000E+00 | | 1.000E+00 | 1.000E+00 | 1.000E+00 |  |
| SizeZoneNonUniformity glszm MYO ED | Size | 0.015 | 0.023 | 0.034 | -0.010 | 1.000E+00 | | 1.000E+00 | 1.000E+00 | 1.000E+00 |  |
| SurfaceArea RV ED | Size | 0.038 | 0.041 | 0.029 | -0.004 | 1.000E+00 | | 3.872E-03 | 3.778E-01 | 1.000E+00 |  |
| Max2DdiameterColumn RV ES | Size | -0.002 | 0.022 | 0.000 | 0.005 | 1.000E+00 | | 1.000E+00 | 1.000E+00 | 1.000E+00 |  |
| LeastAxis RV ES | Size | 0.040 | 0.027 | 0.021 | -0.019 | 1.000E+00 | | 1.000E+00 | 1.000E+00 | 1.000E+00 |  |
| LeastAxis LV ED | Size | 0.028 | 0.014 | 0.006 | -0.046 | 1.000E+00 | | 1.000E+00 | 1.000E+00 | 1.000E+00 |  |
| Volume RV ES | Size | 0.031 | 0.040 | 0.021 | -0.002 | 1.000E+00 | | 7.115E-03 | 1.000E+00 | 1.000E+00 |  |
| MinorAxis LV ED | Size | 0.040 | 0.054 | 0.042 | -0.049 | 1.000E+00 | | 1.739E-04 | 2.078E-02 | 1.000E+00 |  |
| Max2DdiameterSlice LV ED | Size | 0.003 | 0.025 | 0.017 | -0.033 | 1.000E+00 | | 1.000E+00 | 1.000E+00 | 1.000E+00 |  |
| Max2DdiameterRow RV ED | Size | 0.004 | 0.038 | 0.033 | 0.012 | 1.000E+00 | | 2.064E-01 | 6.487E-01 | 1.000E+00 |  |
| SurfaceArea RV ES | Size | 0.025 | 0.039 | 0.024 | 0.002 | 1.000E+00 | | 6.868E-03 | 1.000E+00 | 1.000E+00 |  |
| Volume LV ED | Size | 0.040 | 0.036 | 0.021 | -0.035 | 1.000E+00 | | 3.439E-02 | 1.000E+00 | 1.000E+00 |  |
| Max2DdiameterSlice LV ES | Size | -0.015 | 0.011 | -0.010 | -0.050 | 1.000E+00 | | 1.000E+00 | 1.000E+00 | 1.000E+00 |  |
| Max3Ddiameter LV ED | Size | -0.022 | 0.008 | 0.009 | -0.020 | 1.000E+00 | | 1.000E+00 | 1.000E+00 | 1.000E+00 |  |
| LeastAxis LV ES | Size | 0.000 | 0.022 | 0.003 | -0.056 | 1.000E+00 | | 1.000E+00 | 1.000E+00 | 1.000E+00 |  |
| Max2DdiameterRow LV ED | Size | -0.003 | 0.016 | 0.012 | -0.019 | 1.000E+00 | | 1.000E+00 | 1.000E+00 | 1.000E+00 |  |
| Max3Ddiameter LV ES | Size | -0.036 | 0.011 | 0.007 | -0.027 | 1.000E+00 | | 1.000E+00 | 1.000E+00 | 1.000E+00 |  |
| Max3Ddiameter RV ED | Size | 0.002 | 0.010 | 0.022 | -0.004 | 1.000E+00 | | 1.000E+00 | 1.000E+00 | 1.000E+00 |  |
| MinorAxis LV ES | Size | 0.005 | 0.028 | 0.014 | -0.072 | 1.000E+00 | | 1.000E+00 | 1.000E+00 | 7.246E-01 |  |
| Max2DdiameterRow LV ES | Size | -0.022 | 0.017 | 0.003 | -0.033 | 1.000E+00 | | 1.000E+00 | 1.000E+00 | 1.000E+00 |  |
| SurfaceArea LV ED | Size | 0.031 | 0.026 | 0.020 | -0.042 | 1.000E+00 | | 1.000E+00 | 1.000E+00 | 1.000E+00 |  |
| MajorAxis LV ES | Size | -0.016 | 0.017 | -0.006 | -0.030 | 1.000E+00 | | 1.000E+00 | 1.000E+00 | 1.000E+00 |  |
| Max2DdiameterColumn LV ES | Size | -0.025 | 0.014 | 0.003 | -0.030 | 1.000E+00 | | 1.000E+00 | 1.000E+00 | 1.000E+00 |  |
| MajorAxis LV ED | Size | 0.000 | 0.012 | 0.007 | -0.014 | 1.000E+00 | | 1.000E+00 | 1.000E+00 | 1.000E+00 |  |
| Max2DdiameterColumn LV ED | Size | 0.013 | 0.019 | 0.015 | -0.021 | 1.000E+00 | | 1.000E+00 | 1.000E+00 | 1.000E+00 |  |
| Volume LV ES | Size | 0.008 | 0.035 | 0.009 | -0.055 | 1.000E+00 | | 2.078E-01 | 1.000E+00 | 1.000E+00 |  |
| SurfaceArea LV ES | Size | 0.003 | 0.023 | 0.003 | -0.055 | 1.000E+00 | | 1.000E+00 | 1.000E+00 | 1.000E+00 |  |
| Max2DdiameterSlice RV ED | Size | 0.006 | 0.035 | 0.037 | 0.015 | 1.000E+00 | | 1.000E+00 | 4.776E-01 | 1.000E+00 |  |
| Max2DdiameterRow RV ES | Size | 0.002 | 0.037 | 0.030 | 0.034 | 1.000E+00 | | 5.778E-01 | 1.000E+00 | 1.000E+00 |  |
| MajorAxis RV ED | Size | 0.012 | 0.030 | 0.030 | -0.010 | 1.000E+00 | | 1.000E+00 | 1.000E+00 | 1.000E+00 |  |
| RunLengthNonUniformity glrlm MYO ED | Size | 0.041 | 0.036 | 0.033 | -0.030 | 1.000E+00 | | 9.754E-01 | 1.000E+00 | 1.000E+00 |  |
| RunLengthNonUniformity glrlm MYO ES | Size | 0.027 | 0.024 | 0.023 | -0.026 | 1.000E+00 | | 1.000E+00 | 1.000E+00 | 1.000E+00 |  |
| Max3Ddiameter RV ES | Size | 0.008 | 0.013 | 0.015 | 0.006 | 1.000E+00 | | 1.000E+00 | 1.000E+00 | 1.000E+00 |  |
| DependenceNonUniformity gldm MYO ED | Size | 0.042 | 0.034 | 0.022 | -0.017 | 1.000E+00 | | 1.000E+00 | 1.000E+00 | 1.000E+00 |  |
| Max2DdiameterSlice RV ES | Size | -0.018 | 0.017 | 0.023 | 0.055 | 1.000E+00 | | 1.000E+00 | 1.000E+00 | 1.000E+00 |  |
| GrayLevelNonUniformity glszm MYO ES | Size | -0.045 | 0.001 | -0.009 | -0.013 | 1.000E+00 | | 1.000E+00 | 1.000E+00 | 1.000E+00 |  |
| SizeZoneNonUniformity glszm MYO ES | Size | -0.045 | 0.001 | -0.009 | -0.013 | 1.000E+00 | | 1.000E+00 | 1.000E+00 | 1.000E+00 |  |
| MajorAxis RV ES | Size | -0.007 | 0.023 | 0.016 | -0.011 | 1.000E+00 | | 1.000E+00 | 1.000E+00 | 1.000E+00 |  |

**Supplementary Table 8. Association of vascular risk factors with radiomics features in fully adjusted linear regression models stratified for men and women**

|  |  |  | **Beta** | | | | **Adjusted p-value** | | | |
| --- | --- | --- | --- | --- | --- | --- | --- | --- | --- | --- |
| **Sex** | **Features** | **Cluster** | **Diabetes** | **High chol.** | **Hypertension** | **Smoker** | **Diabetes** | **High chol.** | **Hypertension** | **Smoker** |
| M | Autocorrelation glcm MYO ED | Global Intensity | 0.031 | -0.037 | -0.038 | 0.016 | 1.000E+00 | 1.000E+00 | 1.000E+00 | 1.000E+00 |
| F | Autocorrelation glcm MYO ED | Global Intensity | -0.032 | -0.053 | -0.009 | 0.160 | 1.000E+00 | 1.000E+00 | 1.000E+00 | 2.961E-01 |
| M | Autocorrelation glcm MYO ES | Global Intensity | -0.075 | -0.043 | -0.091 | -0.037 | 1.000E+00 | 1.000E+00 | 6.609E-04 | 1.000E+00 |
| F | Autocorrelation glcm MYO ES | Global Intensity | -0.165 | -0.109 | -0.120 | 0.029 | 2.783E-02 | 3.952E-06 | 1.865E-07 | 1.000E+00 |
| M | ClusterTendency glcm MYO ES | Global Intensity | -0.259 | -0.080 | -0.097 | -0.110 | 3.100E-12 | 3.684E-03 | 2.645E-05 | 1.000E+00 |
| F | ClusterTendency glcm MYO ES | Global Intensity | -0.252 | -0.046 | -0.108 | -0.020 | 8.313E-06 | 1.000E+00 | 5.605E-05 | 1.000E+00 |
| M | Complexity ngtdm MYO ED | Global Intensity | -0.082 | -0.019 | -0.036 | -0.029 | 5.196E-01 | 1.000E+00 | 1.000E+00 | 1.000E+00 |
| F | Complexity ngtdm MYO ED | Global Intensity | -0.132 | -0.049 | -0.141 | -0.116 | 9.684E-01 | 1.000E+00 | 3.283E-09 | 1.000E+00 |
| M | Correlation glcm MYO ED | Global Intensity | -0.071 | -0.017 | 0.104 | 0.106 | 1.000E+00 | 1.000E+00 | 7.033E-05 | 1.000E+00 |
| F | Correlation glcm MYO ED | Global Intensity | -0.021 | -0.015 | 0.103 | 0.065 | 1.000E+00 | 1.000E+00 | 4.374E-06 | 1.000E+00 |
| M | DependenceEntropy gldm MYO ED | Global Intensity | -0.110 | -0.008 | 0.109 | 0.095 | 8.120E-01 | 1.000E+00 | 2.095E-05 | 1.000E+00 |
| F | DependenceEntropy gldm MYO ED | Global Intensity | -0.145 | -0.025 | 0.145 | 0.054 | 8.430E-02 | 1.000E+00 | 1.429E-12 | 1.000E+00 |
| M | Energy MYO ED | Global Intensity | -0.353 | -0.141 | 0.114 | -0.029 | 2.462E-18 | 2.723E-09 | 5.633E-06 | 1.000E+00 |
| F | Energy MYO ED | Global Intensity | -0.251 | -0.106 | 0.124 | -0.019 | 1.226E-08 | 1.671E-07 | 2.416E-10 | 1.000E+00 |
| M | Energy MYO ES | Global Intensity | -0.403 | -0.191 | 0.076 | -0.103 | 1.201E-22 | 1.335E-16 | 7.896E-02 | 1.000E+00 |
| F | Energy MYO ES | Global Intensity | -0.272 | -0.125 | 0.096 | -0.025 | 2.903E-13 | 9.738E-14 | 1.198E-07 | 1.000E+00 |
| M | Entropy MYO ES | Global Intensity | -0.284 | -0.106 | -0.148 | -0.170 | 2.023E-12 | 3.426E-05 | 2.169E-11 | 1.759E-02 |
| F | Entropy MYO ES | Global Intensity | -0.280 | -0.104 | -0.182 | -0.130 | 2.488E-10 | 1.108E-06 | 4.846E-22 | 6.107E-01 |
| M | GrayLevelVariance gldm MYO ES | Global Intensity | -0.214 | -0.070 | -0.114 | -0.095 | 4.798E-09 | 1.510E-02 | 1.145E-08 | 1.000E+00 |
| F | GrayLevelVariance gldm MYO ES | Global Intensity | -0.238 | -0.060 | -0.149 | -0.060 | 5.965E-05 | 8.838E-01 | 3.024E-10 | 1.000E+00 |
| M | GrayLevelVariance glrlm MYO ES | Global Intensity | -0.186 | -0.060 | -0.112 | -0.093 | 1.420E-06 | 1.487E-01 | 2.208E-08 | 1.000E+00 |
| F | GrayLevelVariance glrlm MYO ES | Global Intensity | -0.239 | -0.068 | -0.146 | -0.063 | 5.941E-05 | 2.690E-01 | 1.239E-09 | 1.000E+00 |
| M | HighGrayLevelEmphasis gldm MYO ED | Global Intensity | 0.012 | -0.035 | -0.048 | 0.004 | 1.000E+00 | 1.000E+00 | 1.000E+00 | 1.000E+00 |
| F | HighGrayLevelEmphasis gldm MYO ED | Global Intensity | -0.051 | -0.052 | -0.033 | 0.122 | 1.000E+00 | 1.000E+00 | 1.000E+00 | 1.000E+00 |
| M | HighGrayLevelEmphasis gldm MYO ES | Global Intensity | -0.083 | -0.044 | -0.095 | -0.034 | 1.000E+00 | 1.000E+00 | 2.188E-04 | 1.000E+00 |
| F | HighGrayLevelEmphasis gldm MYO ES | Global Intensity | -0.169 | -0.110 | -0.125 | 0.027 | 1.796E-02 | 3.240E-06 | 3.968E-08 | 1.000E+00 |
| M | HighGrayLevelRunEmphasis glrlm MYO ED | Global Intensity | -0.002 | -0.039 | -0.052 | -0.006 | 1.000E+00 | 1.000E+00 | 5.231E-01 | 1.000E+00 |
| F | HighGrayLevelRunEmphasis glrlm MYO ED | Global Intensity | -0.060 | -0.051 | -0.038 | 0.109 | 1.000E+00 | 1.000E+00 | 1.000E+00 | 1.000E+00 |
| M | HighGrayLevelRunEmphasis glrlm MYO ES | Global Intensity | -0.098 | -0.047 | -0.096 | -0.030 | 1.000E+00 | 1.000E+00 | 1.720E-04 | 1.000E+00 |
| F | HighGrayLevelRunEmphasis glrlm MYO ES | Global Intensity | -0.170 | -0.108 | -0.127 | 0.026 | 1.785E-02 | 6.179E-06 | 2.246E-08 | 1.000E+00 |
| M | HighGrayLevelZoneEmphasis glszm MYO ED | Global Intensity | -0.081 | -0.052 | -0.086 | -0.052 | 1.000E+00 | 5.112E-01 | 5.458E-05 | 1.000E+00 |
| F | HighGrayLevelZoneEmphasis glszm MYO ED | Global Intensity | -0.138 | -0.069 | -0.086 | 0.000 | 3.223E-01 | 9.650E-02 | 2.777E-03 | 1.000E+00 |
| M | HighGrayLevelZoneEmphasis glszm MYO ES | Global Intensity | -0.190 | -0.078 | -0.131 | -0.041 | 1.749E-05 | 1.539E-02 | 2.400E-09 | 1.000E+00 |
| F | HighGrayLevelZoneEmphasis glszm MYO ES | Global Intensity | -0.214 | -0.116 | -0.162 | -0.073 | 1.589E-04 | 6.002E-07 | 4.070E-14 | 1.000E+00 |
| M | InformalMeasureofCorrelation2 glcm MYO ED | Global Intensity | -0.092 | -0.014 | 0.075 | 0.066 | 1.000E+00 | 1.000E+00 | 7.559E-02 | 1.000E+00 |
| F | InformalMeasureofCorrelation2 glcm MYO ED | Global Intensity | -0.026 | -0.032 | 0.021 | 0.046 | 1.000E+00 | 1.000E+00 | 1.000E+00 | 1.000E+00 |
| M | InterquartileRange MYO ES | Global Intensity | -0.323 | -0.114 | -0.124 | -0.254 | 3.900E-16 | 4.144E-06 | 1.140E-07 | 8.884E-07 |
| F | InterquartileRange MYO ES | Global Intensity | -0.258 | -0.096 | -0.152 | -0.148 | 1.050E-07 | 5.323E-05 | 1.497E-13 | 2.548E-01 |
| M | JointAverage glcm MYO ED | Global Intensity | 0.040 | -0.037 | -0.040 | 0.018 | 1.000E+00 | 1.000E+00 | 1.000E+00 | 1.000E+00 |
| F | JointAverage glcm MYO ED | Global Intensity | -0.030 | -0.052 | -0.007 | 0.158 | 1.000E+00 | 1.000E+00 | 1.000E+00 | 2.349E-01 |
| M | JointAverage glcm MYO ES | Global Intensity | -0.072 | -0.040 | -0.089 | -0.030 | 1.000E+00 | 1.000E+00 | 1.756E-03 | 1.000E+00 |
| F | JointAverage glcm MYO ES | Global Intensity | -0.165 | -0.111 | -0.120 | 0.021 | 1.765E-02 | 8.122E-07 | 6.410E-08 | 1.000E+00 |
| M | JointEntropy glcm MYO ES | Global Intensity | -0.240 | -0.089 | -0.137 | -0.131 | 5.715E-09 | 1.533E-03 | 5.471E-10 | 4.638E-01 |
| F | JointEntropy glcm MYO ES | Global Intensity | -0.261 | -0.092 | -0.167 | -0.120 | 1.012E-08 | 5.952E-05 | 3.122E-18 | 1.000E+00 |
| M | Maximum MYO ED | Global Intensity | -0.148 | -0.040 | -0.031 | -0.036 | 9.275E-04 | 1.000E+00 | 1.000E+00 | 1.000E+00 |
| F | Maximum MYO ED | Global Intensity | -0.153 | -0.059 | -0.095 | -0.154 | 6.545E-02 | 4.867E-01 | 1.915E-04 | 2.162E-01 |
| M | Mean MYO ED | Global Intensity | -0.163 | -0.080 | -0.031 | -0.085 | 7.215E-05 | 1.029E-03 | 1.000E+00 | 1.000E+00 |
| F | Mean MYO ED | Global Intensity | -0.166 | -0.094 | -0.038 | -0.116 | 1.239E-02 | 1.005E-04 | 1.000E+00 | 1.000E+00 |
| M | Mean MYO ES | Global Intensity | -0.272 | -0.148 | -0.098 | -0.168 | 4.293E-13 | 6.924E-13 | 3.264E-05 | 6.697E-03 |
| F | Mean MYO ES | Global Intensity | -0.267 | -0.142 | -0.103 | -0.148 | 7.786E-09 | 1.117E-12 | 4.607E-06 | 1.890E-01 |
| M | MeanAbsoluteDeviation MYO ES | Global Intensity | -0.293 | -0.096 | -0.133 | -0.195 | 3.357E-14 | 2.133E-04 | 1.153E-09 | 6.341E-04 |
| F | MeanAbsoluteDeviation MYO ES | Global Intensity | -0.265 | -0.086 | -0.176 | -0.114 | 1.086E-07 | 1.612E-03 | 3.101E-17 | 1.000E+00 |
| M | Median MYO ED | Global Intensity | -0.153 | -0.086 | -0.022 | -0.080 | 5.213E-04 | 3.209E-04 | 1.000E+00 | 1.000E+00 |
| F | Median MYO ED | Global Intensity | -0.157 | -0.098 | -0.010 | -0.089 | 3.478E-02 | 3.984E-05 | 1.000E+00 | 1.000E+00 |
| M | Median MYO ES | Global Intensity | -0.256 | -0.147 | -0.093 | -0.168 | 1.523E-11 | 1.004E-12 | 1.149E-04 | 6.593E-03 |
| F | Median MYO ES | Global Intensity | -0.254 | -0.145 | -0.101 | -0.142 | 5.273E-08 | 1.864E-13 | 6.611E-06 | 2.843E-01 |
| M | Percentile10 MYO ED | Global Intensity | -0.205 | -0.114 | -0.003 | -0.114 | 5.067E-07 | 3.242E-07 | 1.000E+00 | 1.000E+00 |
| F | Percentile10 MYO ED | Global Intensity | -0.175 | -0.113 | 0.062 | -0.113 | 1.269E-02 | 1.850E-06 | 3.638E-01 | 1.000E+00 |
| M | Percentile10 MYO ES | Global Intensity | -0.224 | -0.139 | -0.078 | -0.121 | 4.165E-09 | 7.811E-12 | 3.690E-03 | 4.444E-01 |
| F | Percentile10 MYO ES | Global Intensity | -0.227 | -0.138 | -0.064 | -0.133 | 7.379E-06 | 1.904E-11 | 1.346E-01 | 7.324E-01 |
| M | Percentile90 MYO ED | Global Intensity | -0.137 | -0.048 | -0.056 | -0.073 | 7.011E-04 | 6.885E-01 | 9.120E-02 | 1.000E+00 |
| F | Percentile90 MYO ED | Global Intensity | -0.159 | -0.077 | -0.116 | -0.133 | 1.991E-02 | 6.112E-03 | 5.760E-08 | 6.699E-01 |
| M | Percentile90 MYO ES | Global Intensity | -0.307 | -0.146 | -0.111 | -0.191 | 1.808E-16 | 5.057E-12 | 8.633E-07 | 6.012E-04 |
| F | Percentile90 MYO ES | Global Intensity | -0.296 | -0.130 | -0.123 | -0.133 | 8.793E-11 | 3.144E-10 | 7.732E-09 | 6.903E-01 |
| M | Range MYO ED | Global Intensity | -0.120 | -0.033 | -0.033 | -0.015 | 2.837E-02 | 1.000E+00 | 1.000E+00 | 1.000E+00 |
| F | Range MYO ED | Global Intensity | -0.141 | -0.059 | -0.106 | -0.127 | 2.352E-01 | 5.558E-01 | 1.215E-05 | 1.000E+00 |
| M | RobustMeanAbsoluteDeviation MYO ES | Global Intensity | -0.316 | -0.112 | -0.123 | -0.240 | 9.441E-16 | 5.144E-06 | 9.845E-08 | 4.182E-06 |
| F | RobustMeanAbsoluteDeviation MYO ES | Global Intensity | -0.267 | -0.088 | -0.152 | -0.121 | 3.786E-08 | 6.717E-04 | 3.038E-13 | 1.000E+00 |
| M | RootMeanSquared MYO ED | Global Intensity | -0.156 | -0.073 | -0.036 | -0.081 | 1.396E-04 | 5.717E-03 | 1.000E+00 | 1.000E+00 |
| F | RootMeanSquared MYO ED | Global Intensity | -0.163 | -0.088 | -0.056 | -0.118 | 1.602E-02 | 4.608E-04 | 5.747E-01 | 1.000E+00 |
| M | RootMeanSquared MYO ES | Global Intensity | -0.276 | -0.147 | -0.101 | -0.169 | 1.763E-13 | 1.146E-12 | 1.195E-05 | 5.828E-03 |
| F | RootMeanSquared MYO ES | Global Intensity | -0.271 | -0.141 | -0.108 | -0.148 | 4.140E-09 | 1.611E-12 | 7.796E-07 | 1.978E-01 |
| M | RunEntropy glrlm MYO ED | Global Intensity | -0.108 | -0.029 | 0.127 | 0.058 | 8.987E-01 | 1.000E+00 | 6.657E-08 | 1.000E+00 |
| F | RunEntropy glrlm MYO ED | Global Intensity | -0.150 | -0.035 | 0.030 | -0.014 | 8.112E-02 | 1.000E+00 | 1.000E+00 | 1.000E+00 |
| M | ShortRunHighGrayLevelEmphasis glrlm MYO ED | Global Intensity | -0.011 | -0.032 | -0.076 | -0.022 | 1.000E+00 | 1.000E+00 | 3.984E-04 | 1.000E+00 |
| F | ShortRunHighGrayLevelEmphasis glrlm MYO ED | Global Intensity | -0.065 | -0.052 | -0.082 | 0.072 | 1.000E+00 | 1.000E+00 | 1.221E-02 | 1.000E+00 |
| M | ShortRunHighGrayLevelEmphasis glrlm MYO ES | Global Intensity | -0.097 | -0.043 | -0.123 | -0.028 | 1.000E+00 | 1.000E+00 | 7.947E-09 | 1.000E+00 |
| F | ShortRunHighGrayLevelEmphasis glrlm MYO ES | Global Intensity | -0.169 | -0.109 | -0.168 | 0.004 | 2.567E-02 | 8.676E-06 | 8.379E-15 | 1.000E+00 |
| M | SmallAreaHighGrayLevelEmphasis glszm MYO ED | Global Intensity | -0.089 | -0.052 | -0.076 | -0.043 | 7.190E-01 | 4.281E-01 | 8.338E-04 | 1.000E+00 |
| F | SmallAreaHighGrayLevelEmphasis glszm MYO ED | Global Intensity | -0.155 | -0.083 | -0.105 | -0.016 | 7.902E-02 | 5.043E-03 | 2.365E-05 | 1.000E+00 |
| M | SmallAreaHighGrayLevelEmphasis glszm MYO ES | Global Intensity | -0.190 | -0.079 | -0.128 | -0.051 | 1.059E-05 | 8.740E-03 | 2.344E-09 | 1.000E+00 |
| F | SmallAreaHighGrayLevelEmphasis glszm MYO ES | Global Intensity | -0.171 | -0.129 | -0.173 | -0.125 | 2.190E-02 | 1.189E-08 | 6.730E-16 | 1.000E+00 |
| M | SmallDependenceHighGrayLevelEmphasis gldm MYO ED | Global Intensity | -0.046 | -0.018 | -0.086 | -0.058 | 1.000E+00 | 1.000E+00 | 8.010E-11 | 1.000E+00 |
| F | SmallDependenceHighGrayLevelEmphasis gldm MYO ED | Global Intensity | -0.087 | -0.069 | -0.220 | -0.069 | 1.000E+00 | 1.095E-01 | 5.478E-26 | 1.000E+00 |
| M | SmallDependenceHighGrayLevelEmphasis gldm MYO ES | Global Intensity | -0.095 | -0.028 | -0.149 | -0.049 | 2.245E-01 | 1.000E+00 | 2.927E-19 | 1.000E+00 |
| F | SmallDependenceHighGrayLevelEmphasis gldm MYO ES | Global Intensity | -0.128 | -0.113 | -0.247 | -0.096 | 1.000E+00 | 1.196E-05 | 1.721E-29 | 1.000E+00 |
| M | Strength ngtdm MYO ED | Global Intensity | -0.021 | -0.006 | -0.021 | -0.008 | 1.000E+00 | 1.000E+00 | 1.000E+00 | 1.000E+00 |
| F | Strength ngtdm MYO ED | Global Intensity | -0.023 | -0.003 | -0.119 | -0.125 | 1.000E+00 | 1.000E+00 | 8.134E-06 | 1.000E+00 |
| M | SumAverage glcm MYO ED | Global Intensity | 0.040 | -0.037 | -0.040 | 0.018 | 1.000E+00 | 1.000E+00 | 1.000E+00 | 1.000E+00 |
| F | SumAverage glcm MYO ED | Global Intensity | -0.030 | -0.052 | -0.007 | 0.158 | 1.000E+00 | 1.000E+00 | 1.000E+00 | 2.349E-01 |
| M | SumAverage glcm MYO ES | Global Intensity | -0.072 | -0.040 | -0.089 | -0.030 | 1.000E+00 | 1.000E+00 | 1.756E-03 | 1.000E+00 |
| F | SumAverage glcm MYO ES | Global Intensity | -0.165 | -0.111 | -0.120 | 0.021 | 1.765E-02 | 8.122E-07 | 6.410E-08 | 1.000E+00 |
| M | SumEntropy glcm MYO ES | Global Intensity | -0.302 | -0.109 | -0.131 | -0.165 | 4.125E-13 | 4.579E-05 | 4.792E-08 | 4.842E-02 |
| F | SumEntropy glcm MYO ES | Global Intensity | -0.286 | -0.095 | -0.144 | -0.113 | 4.566E-11 | 1.299E-05 | 8.921E-14 | 1.000E+00 |
| M | SumofSquares glcm MYO ES | Global Intensity | -0.223 | -0.069 | -0.104 | -0.088 | 2.793E-09 | 3.439E-02 | 1.163E-06 | 1.000E+00 |
| F | SumofSquares glcm MYO ES | Global Intensity | -0.239 | -0.050 | -0.126 | -0.047 | 4.235E-05 | 1.000E+00 | 3.096E-07 | 1.000E+00 |
| M | TotalEnergy MYO ED | Global Intensity | -0.321 | -0.124 | 0.151 | 0.008 | 8.525E-16 | 1.982E-07 | 9.883E-12 | 1.000E+00 |
| F | TotalEnergy MYO ED | Global Intensity | -0.219 | -0.091 | 0.148 | -0.010 | 8.871E-07 | 1.465E-05 | 4.707E-16 | 1.000E+00 |
| M | TotalEnergy MYO ES | Global Intensity | -0.384 | -0.179 | 0.106 | -0.074 | 2.589E-21 | 4.364E-15 | 8.231E-05 | 1.000E+00 |
| F | TotalEnergy MYO ES | Global Intensity | -0.252 | -0.113 | 0.118 | -0.016 | 8.988E-12 | 1.290E-11 | 2.015E-12 | 1.000E+00 |
| M | Variance MYO ES | Global Intensity | -0.204 | -0.060 | -0.109 | -0.105 | 1.733E-08 | 1.061E-01 | 3.433E-08 | 9.543E-01 |
| F | Variance MYO ES | Global Intensity | -0.222 | -0.056 | -0.153 | -0.053 | 6.370E-04 | 1.000E+00 | 2.847E-10 | 1.000E+00 |
| M | ZoneEntropy glszm MYO ES | Global Intensity | -0.215 | -0.063 | -0.120 | -0.068 | 1.370E-06 | 4.088E-01 | 4.131E-07 | 1.000E+00 |
| F | ZoneEntropy glszm MYO ES | Global Intensity | -0.267 | -0.107 | -0.094 | -0.028 | 5.476E-08 | 3.740E-06 | 1.976E-04 | 1.000E+00 |
| M | ClusterTendency glcm MYO ED | Global Variance | -0.062 | 0.008 | -0.043 | -0.009 | 1.000E+00 | 1.000E+00 | 4.542E-01 | 1.000E+00 |
| F | ClusterTendency glcm MYO ED | Global Variance | -0.096 | -0.030 | -0.172 | -0.050 | 1.000E+00 | 1.000E+00 | 3.261E-14 | 1.000E+00 |
| M | Contrast glcm MYO ED | Global Variance | -0.054 | 0.005 | -0.077 | -0.050 | 1.000E+00 | 1.000E+00 | 5.303E-07 | 1.000E+00 |
| F | Contrast glcm MYO ED | Global Variance | -0.104 | -0.053 | -0.240 | -0.136 | 1.000E+00 | 1.000E+00 | 2.322E-34 | 6.691E-01 |
| M | Contrast glcm MYO ES | Global Variance | -0.078 | -0.024 | -0.100 | -0.010 | 1.000E+00 | 1.000E+00 | 1.970E-07 | 1.000E+00 |
| F | Contrast glcm MYO ES | Global Variance | -0.147 | -0.047 | -0.144 | -0.105 | 2.463E-01 | 1.000E+00 | 3.254E-10 | 1.000E+00 |
| M | Contrast ngtdm MYO ED | Global Variance | -0.006 | 0.040 | -0.079 | -0.023 | 1.000E+00 | 1.000E+00 | 5.228E-06 | 1.000E+00 |
| F | Contrast ngtdm MYO ED | Global Variance | -0.037 | -0.006 | -0.217 | -0.126 | 1.000E+00 | 1.000E+00 | 9.465E-26 | 1.000E+00 |
| M | Contrast ngtdm MYO ES | Global Variance | -0.083 | -0.040 | -0.067 | -0.091 | 1.000E+00 | 1.000E+00 | 5.544E-02 | 1.000E+00 |
| F | Contrast ngtdm MYO ES | Global Variance | -0.110 | 0.018 | -0.077 | -0.037 | 1.000E+00 | 1.000E+00 | 3.151E-02 | 1.000E+00 |
| M | DependenceNonUniformityNormalized gldm MYO ED | Global Variance | 0.019 | 0.022 | -0.104 | -0.020 | 1.000E+00 | 1.000E+00 | 5.527E-11 | 1.000E+00 |
| F | DependenceNonUniformityNormalized gldm MYO ED | Global Variance | 0.026 | -0.030 | -0.228 | -0.071 | 1.000E+00 | 1.000E+00 | 6.483E-34 | 1.000E+00 |
| M | DependenceNonUniformityNormalized gldm MYO ES | Global Variance | -0.078 | -0.030 | -0.077 | -0.034 | 1.000E+00 | 1.000E+00 | 1.001E-03 | 1.000E+00 |
| F | DependenceNonUniformityNormalized gldm MYO ES | Global Variance | -0.036 | -0.012 | -0.154 | -0.091 | 1.000E+00 | 1.000E+00 | 2.194E-11 | 1.000E+00 |
| M | DifferenceAverage glcm MYO ED | Global Variance | -0.034 | 0.009 | -0.101 | -0.039 | 1.000E+00 | 1.000E+00 | 5.069E-10 | 1.000E+00 |
| F | DifferenceAverage glcm MYO ED | Global Variance | -0.088 | -0.046 | -0.223 | -0.120 | 1.000E+00 | 1.000E+00 | 1.014E-37 | 7.105E-01 |
| M | DifferenceAverage glcm MYO ES | Global Variance | -0.082 | -0.037 | -0.115 | -0.018 | 1.000E+00 | 1.000E+00 | 1.169E-08 | 1.000E+00 |
| F | DifferenceAverage glcm MYO ES | Global Variance | -0.154 | -0.061 | -0.162 | -0.110 | 3.344E-02 | 1.792E-01 | 3.766E-16 | 1.000E+00 |
| M | DifferenceEntropy glcm MYO ED | Global Variance | -0.059 | 0.007 | -0.120 | -0.068 | 1.000E+00 | 1.000E+00 | 1.388E-12 | 1.000E+00 |
| F | DifferenceEntropy glcm MYO ED | Global Variance | -0.110 | -0.049 | -0.226 | -0.145 | 3.548E-01 | 4.070E-01 | 3.061E-44 | 3.284E-02 |
| M | DifferenceEntropy glcm MYO ES | Global Variance | -0.093 | -0.034 | -0.132 | -0.042 | 1.000E+00 | 1.000E+00 | 4.631E-11 | 1.000E+00 |
| F | DifferenceEntropy glcm MYO ES | Global Variance | -0.154 | -0.071 | -0.178 | -0.126 | 1.707E-02 | 1.033E-02 | 1.536E-21 | 7.379E-01 |
| M | DifferenceVariance glcm MYO ED | Global Variance | -0.075 | 0.001 | -0.075 | -0.068 | 5.614E-01 | 1.000E+00 | 4.994E-06 | 1.000E+00 |
| F | DifferenceVariance glcm MYO ED | Global Variance | -0.123 | -0.057 | -0.235 | -0.159 | 7.576E-01 | 6.073E-01 | 2.199E-32 | 1.355E-01 |
| M | DifferenceVariance glcm MYO ES | Global Variance | -0.088 | -0.017 | -0.100 | -0.022 | 9.195E-01 | 1.000E+00 | 3.639E-07 | 1.000E+00 |
| F | DifferenceVariance glcm MYO ES | Global Variance | -0.146 | -0.047 | -0.144 | -0.109 | 2.471E-01 | 1.000E+00 | 3.028E-10 | 1.000E+00 |
| M | Entropy MYO ED | Global Variance | -0.051 | 0.013 | -0.091 | -0.019 | 1.000E+00 | 1.000E+00 | 2.909E-06 | 1.000E+00 |
| F | Entropy MYO ED | Global Variance | -0.105 | -0.041 | -0.174 | -0.098 | 7.184E-01 | 1.000E+00 | 1.246E-24 | 1.000E+00 |
| M | GrayLevelVariance gldm MYO ED | Global Variance | -0.058 | 0.010 | -0.056 | -0.023 | 1.000E+00 | 1.000E+00 | 9.173E-03 | 1.000E+00 |
| F | GrayLevelVariance gldm MYO ED | Global Variance | -0.101 | -0.018 | -0.190 | -0.086 | 1.000E+00 | 1.000E+00 | 1.293E-18 | 1.000E+00 |
| M | GrayLevelVariance glrlm MYO ED | Global Variance | -0.061 | 0.009 | -0.050 | -0.025 | 1.000E+00 | 1.000E+00 | 8.471E-02 | 1.000E+00 |
| F | GrayLevelVariance glrlm MYO ED | Global Variance | -0.106 | -0.016 | -0.183 | -0.089 | 1.000E+00 | 1.000E+00 | 2.830E-17 | 1.000E+00 |
| M | GrayLevelVariance glszm MYO ED | Global Variance | -0.038 | 0.008 | -0.011 | 0.032 | 1.000E+00 | 1.000E+00 | 1.000E+00 | 1.000E+00 |
| F | GrayLevelVariance glszm MYO ED | Global Variance | -0.079 | -0.011 | -0.122 | -0.020 | 1.000E+00 | 1.000E+00 | 1.061E-07 | 1.000E+00 |
| M | InterquartileRange MYO ED | Global Variance | -0.043 | 0.018 | -0.037 | 0.010 | 1.000E+00 | 1.000E+00 | 1.000E+00 | 1.000E+00 |
| F | InterquartileRange MYO ED | Global Variance | -0.104 | -0.021 | -0.144 | -0.074 | 1.000E+00 | 1.000E+00 | 1.305E-11 | 1.000E+00 |
| M | InverseVariance glcm MYO ED | Global Variance | 0.022 | 0.023 | -0.107 | 0.003 | 1.000E+00 | 1.000E+00 | 1.232E-05 | 1.000E+00 |
| F | InverseVariance glcm MYO ED | Global Variance | -0.041 | -0.017 | -0.088 | -0.030 | 1.000E+00 | 1.000E+00 | 7.694E-08 | 1.000E+00 |
| M | InverseVariance glcm MYO ES | Global Variance | -0.086 | -0.047 | -0.109 | -0.026 | 1.000E+00 | 1.000E+00 | 3.521E-06 | 1.000E+00 |
| F | InverseVariance glcm MYO ES | Global Variance | -0.149 | -0.059 | -0.136 | -0.089 | 1.469E-02 | 9.903E-02 | 1.956E-13 | 1.000E+00 |
| M | JointEntropy glcm MYO ED | Global Variance | -0.042 | 0.012 | -0.090 | -0.021 | 1.000E+00 | 1.000E+00 | 5.685E-06 | 1.000E+00 |
| F | JointEntropy glcm MYO ED | Global Variance | -0.097 | -0.047 | -0.176 | -0.090 | 1.000E+00 | 8.065E-01 | 2.640E-25 | 1.000E+00 |
| M | MeanAbsoluteDeviation MYO ED | Global Variance | -0.059 | 0.012 | -0.070 | -0.019 | 1.000E+00 | 1.000E+00 | 3.125E-04 | 1.000E+00 |
| F | MeanAbsoluteDeviation MYO ED | Global Variance | -0.108 | -0.026 | -0.191 | -0.111 | 1.000E+00 | 1.000E+00 | 3.226E-23 | 1.000E+00 |
| M | RobustMeanAbsoluteDeviation MYO ED | Global Variance | -0.046 | 0.014 | -0.044 | -0.001 | 1.000E+00 | 1.000E+00 | 5.781E-01 | 1.000E+00 |
| F | RobustMeanAbsoluteDeviation MYO ED | Global Variance | -0.112 | -0.023 | -0.165 | -0.089 | 1.000E+00 | 1.000E+00 | 8.616E-16 | 1.000E+00 |
| M | RunLengthNonUniformityNormalized glrlm MYO ED | Global Variance | -0.004 | 0.033 | -0.160 | -0.050 | 1.000E+00 | 1.000E+00 | 1.274E-20 | 1.000E+00 |
| F | RunLengthNonUniformityNormalized glrlm MYO ED | Global Variance | -0.029 | -0.024 | -0.224 | -0.113 | 1.000E+00 | 1.000E+00 | 5.410E-50 | 3.288E-01 |
| M | RunLengthNonUniformityNormalized glrlm MYO ES | Global Variance | -0.004 | 0.001 | -0.152 | -0.019 | 1.000E+00 | 1.000E+00 | 1.851E-14 | 1.000E+00 |
| F | RunLengthNonUniformityNormalized glrlm MYO ES | Global Variance | -0.049 | -0.046 | -0.203 | -0.086 | 1.000E+00 | 1.000E+00 | 2.526E-29 | 1.000E+00 |
| M | RunPercentage glrlm MYO ED | Global Variance | 0.005 | 0.032 | -0.160 | -0.049 | 1.000E+00 | 1.000E+00 | 4.331E-19 | 1.000E+00 |
| F | RunPercentage glrlm MYO ED | Global Variance | -0.029 | -0.024 | -0.201 | -0.096 | 1.000E+00 | 1.000E+00 | 2.147E-45 | 9.845E-01 |
| M | RunPercentage glrlm MYO ES | Global Variance | -0.038 | -0.015 | -0.177 | -0.048 | 1.000E+00 | 1.000E+00 | 1.647E-19 | 1.000E+00 |
| F | RunPercentage glrlm MYO ES | Global Variance | -0.075 | -0.049 | -0.213 | -0.105 | 1.000E+00 | 5.932E-01 | 4.522E-36 | 1.000E+00 |
| M | ShortRunEmphasis glrlm MYO ED | Global Variance | -0.007 | 0.033 | -0.169 | -0.057 | 1.000E+00 | 1.000E+00 | 1.947E-21 | 1.000E+00 |
| F | ShortRunEmphasis glrlm MYO ED | Global Variance | -0.043 | -0.024 | -0.214 | -0.116 | 1.000E+00 | 1.000E+00 | 2.209E-52 | 1.043E-01 |
| M | ShortRunEmphasis glrlm MYO ES | Global Variance | 0.000 | 0.004 | -0.159 | -0.023 | 1.000E+00 | 1.000E+00 | 5.072E-15 | 1.000E+00 |
| F | ShortRunEmphasis glrlm MYO ES | Global Variance | -0.059 | -0.049 | -0.198 | -0.090 | 1.000E+00 | 6.542E-01 | 4.808E-31 | 1.000E+00 |
| M | SmallDependenceEmphasis gldm MYO ED | Global Variance | -0.036 | 0.012 | -0.113 | -0.071 | 1.000E+00 | 1.000E+00 | 4.151E-17 | 1.000E+00 |
| F | SmallDependenceEmphasis gldm MYO ED | Global Variance | -0.045 | -0.044 | -0.274 | -0.155 | 1.000E+00 | 1.000E+00 | 3.071E-56 | 3.381E-02 |
| M | SmallDependenceEmphasis gldm MYO ES | Global Variance | -0.015 | 0.020 | -0.166 | -0.054 | 1.000E+00 | 1.000E+00 | 2.507E-24 | 1.000E+00 |
| F | SmallDependenceEmphasis gldm MYO ES | Global Variance | 0.006 | -0.067 | -0.273 | -0.125 | 1.000E+00 | 8.160E-02 | 1.830E-44 | 1.000E+00 |
| M | SumEntropy glcm MYO ED | Global Variance | -0.054 | 0.010 | -0.077 | -0.012 | 1.000E+00 | 1.000E+00 | 1.723E-03 | 1.000E+00 |
| F | SumEntropy glcm MYO ED | Global Variance | -0.099 | -0.050 | -0.149 | -0.076 | 1.000E+00 | 3.913E-01 | 4.121E-18 | 1.000E+00 |
| M | SumofSquares glcm MYO ED | Global Variance | -0.061 | 0.007 | -0.055 | -0.023 | 1.000E+00 | 1.000E+00 | 9.264E-03 | 1.000E+00 |
| F | SumofSquares glcm MYO ED | Global Variance | -0.101 | -0.038 | -0.199 | -0.080 | 1.000E+00 | 1.000E+00 | 8.089E-21 | 1.000E+00 |
| M | Variance MYO ED | Global Variance | -0.057 | 0.011 | -0.055 | -0.023 | 1.000E+00 | 1.000E+00 | 1.038E-02 | 1.000E+00 |
| F | Variance MYO ED | Global Variance | -0.097 | -0.014 | -0.192 | -0.086 | 1.000E+00 | 1.000E+00 | 1.032E-18 | 1.000E+00 |
| M | ZoneEntropy glszm MYO ED | Global Variance | -0.066 | -0.011 | -0.068 | -0.019 | 1.000E+00 | 1.000E+00 | 1.003E-01 | 1.000E+00 |
| F | ZoneEntropy glszm MYO ED | Global Variance | -0.098 | -0.032 | -0.094 | -0.037 | 1.000E+00 | 1.000E+00 | 3.906E-05 | 1.000E+00 |
| M | ZonePercentage glszm MYO ED | Global Variance | -0.042 | 0.009 | -0.097 | -0.080 | 1.000E+00 | 1.000E+00 | 5.710E-13 | 5.665E-01 |
| F | ZonePercentage glszm MYO ED | Global Variance | -0.035 | -0.042 | -0.271 | -0.153 | 1.000E+00 | 1.000E+00 | 1.336E-51 | 6.634E-02 |
| M | ZonePercentage glszm MYO ES | Global Variance | -0.009 | 0.031 | -0.161 | -0.067 | 1.000E+00 | 1.000E+00 | 3.303E-23 | 1.000E+00 |
| F | ZonePercentage glszm MYO ES | Global Variance | 0.012 | -0.060 | -0.263 | -0.107 | 1.000E+00 | 4.409E-01 | 1.106E-38 | 1.000E+00 |
| M | Busyness ngtdm MYO ED | Local Dimness | -0.116 | -0.004 | 0.114 | 0.077 | 7.136E-01 | 1.000E+00 | 1.731E-05 | 1.000E+00 |
| F | Busyness ngtdm MYO ED | Local Dimness | 0.000 | 0.000 | 0.079 | -0.007 | 1.000E+00 | 1.000E+00 | 3.535E-07 | 1.000E+00 |
| M | Busyness ngtdm MYO ES | Local Dimness | -0.017 | -0.010 | 0.181 | 0.049 | 1.000E+00 | 1.000E+00 | 2.244E-15 | 1.000E+00 |
| F | Busyness ngtdm MYO ES | Local Dimness | 0.089 | 0.045 | 0.177 | 0.059 | 1.000E+00 | 3.508E-01 | 2.431E-32 | 1.000E+00 |
| M | LargeAreaLowGrayLevelEmphasis glszm MYO ED | Local Dimness | -0.025 | -0.021 | 0.150 | 0.122 | 1.000E+00 | 1.000E+00 | 1.249E-07 | 1.000E+00 |
| F | LargeAreaLowGrayLevelEmphasis glszm MYO ED | Local Dimness | 0.031 | 0.009 | 0.063 | 0.007 | 1.000E+00 | 1.000E+00 | 1.754E-13 | 1.000E+00 |
| M | LargeAreaLowGrayLevelEmphasis glszm MYO ES | Local Dimness | 0.049 | -0.030 | 0.238 | 0.127 | 1.000E+00 | 1.000E+00 | 1.499E-21 | 1.000E+00 |
| F | LargeAreaLowGrayLevelEmphasis glszm MYO ES | Local Dimness | 0.088 | 0.040 | 0.133 | 0.051 | 8.492E-03 | 5.844E-03 | 8.280E-41 | 1.000E+00 |
| M | LargeDependenceLowGrayLevelEmphasis gldm MYO ED | Local Dimness | -0.061 | 0.002 | 0.121 | 0.020 | 1.000E+00 | 1.000E+00 | 3.622E-06 | 1.000E+00 |
| F | LargeDependenceLowGrayLevelEmphasis gldm MYO ED | Local Dimness | 0.044 | 0.039 | 0.070 | -0.049 | 1.000E+00 | 1.000E+00 | 1.487E-04 | 1.000E+00 |
| M | LargeDependenceLowGrayLevelEmphasis gldm MYO ES | Local Dimness | 0.055 | 0.020 | 0.141 | 0.044 | 1.000E+00 | 1.000E+00 | 2.385E-08 | 1.000E+00 |
| F | LargeDependenceLowGrayLevelEmphasis gldm MYO ES | Local Dimness | 0.141 | 0.095 | 0.142 | 0.044 | 3.475E-03 | 1.964E-08 | 1.478E-19 | 1.000E+00 |
| M | LongRunLowGrayLevelEmphasis glrlm MYO ED | Local Dimness | -0.044 | 0.010 | 0.131 | 0.043 | 1.000E+00 | 1.000E+00 | 6.706E-07 | 1.000E+00 |
| F | LongRunLowGrayLevelEmphasis glrlm MYO ED | Local Dimness | 0.046 | 0.041 | 0.052 | -0.048 | 1.000E+00 | 6.157E-01 | 3.186E-02 | 1.000E+00 |
| M | LongRunLowGrayLevelEmphasis glrlm MYO ES | Local Dimness | 0.053 | 0.021 | 0.173 | 0.062 | 1.000E+00 | 1.000E+00 | 3.325E-12 | 1.000E+00 |
| F | LongRunLowGrayLevelEmphasis glrlm MYO ES | Local Dimness | 0.133 | 0.088 | 0.139 | 0.051 | 1.837E-03 | 1.007E-08 | 3.046E-22 | 1.000E+00 |
| M | LowGrayLevelEmphasis gldm MYO ED | Local Dimness | -0.042 | 0.041 | 0.040 | -0.014 | 1.000E+00 | 1.000E+00 | 1.000E+00 | 1.000E+00 |
| F | LowGrayLevelEmphasis gldm MYO ED | Local Dimness | 0.021 | 0.049 | -0.028 | -0.147 | 1.000E+00 | 1.000E+00 | 1.000E+00 | 2.683E-01 |
| M | LowGrayLevelEmphasis gldm MYO ES | Local Dimness | 0.073 | 0.022 | 0.065 | 0.003 | 1.000E+00 | 1.000E+00 | 6.975E-01 | 1.000E+00 |
| F | LowGrayLevelEmphasis gldm MYO ES | Local Dimness | 0.130 | 0.099 | 0.092 | -0.019 | 1.165E-01 | 8.749E-07 | 1.419E-05 | 1.000E+00 |
| M | LowGrayLevelRunEmphasis glrlm MYO ED | Local Dimness | -0.030 | 0.045 | 0.047 | -0.008 | 1.000E+00 | 1.000E+00 | 1.000E+00 | 1.000E+00 |
| F | LowGrayLevelRunEmphasis glrlm MYO ED | Local Dimness | 0.026 | 0.050 | -0.024 | -0.134 | 1.000E+00 | 1.000E+00 | 1.000E+00 | 6.374E-01 |
| M | LowGrayLevelRunEmphasis glrlm MYO ES | Local Dimness | 0.104 | 0.033 | 0.065 | 0.002 | 1.000E+00 | 1.000E+00 | 7.211E-01 | 1.000E+00 |
| F | LowGrayLevelRunEmphasis glrlm MYO ES | Local Dimness | 0.129 | 0.097 | 0.093 | -0.014 | 1.151E-01 | 1.751E-06 | 7.678E-06 | 1.000E+00 |
| M | LowGrayLevelZoneEmphasis glszm MYO ED | Local Dimness | -0.002 | 0.052 | 0.094 | 0.035 | 1.000E+00 | 1.000E+00 | 1.844E-03 | 1.000E+00 |
| F | LowGrayLevelZoneEmphasis glszm MYO ED | Local Dimness | 0.074 | 0.059 | 0.028 | -0.057 | 1.000E+00 | 1.495E-01 | 1.000E+00 | 1.000E+00 |
| M | LowGrayLevelZoneEmphasis glszm MYO ES | Local Dimness | 0.161 | 0.075 | 0.083 | 0.003 | 7.885E-03 | 1.021E-01 | 1.869E-02 | 1.000E+00 |
| F | LowGrayLevelZoneEmphasis glszm MYO ES | Local Dimness | 0.148 | 0.102 | 0.105 | 0.036 | 4.527E-02 | 2.774E-06 | 1.646E-06 | 1.000E+00 |
| M | ShortRunLowGrayLevelEmphasis glrlm MYO ED | Local Dimness | -0.025 | 0.053 | 0.026 | -0.015 | 1.000E+00 | 1.000E+00 | 1.000E+00 | 1.000E+00 |
| F | ShortRunLowGrayLevelEmphasis glrlm MYO ED | Local Dimness | 0.019 | 0.050 | -0.051 | -0.151 | 1.000E+00 | 1.000E+00 | 1.000E+00 | 2.839E-01 |
| M | ShortRunLowGrayLevelEmphasis glrlm MYO ES | Local Dimness | 0.122 | 0.042 | 0.034 | -0.009 | 4.860E-01 | 1.000E+00 | 1.000E+00 | 1.000E+00 |
| F | ShortRunLowGrayLevelEmphasis glrlm MYO ES | Local Dimness | 0.121 | 0.091 | 0.066 | -0.029 | 3.872E-01 | 4.353E-05 | 4.312E-02 | 1.000E+00 |
| M | SmallAreaLowGrayLevelEmphasis glszm MYO ED | Local Dimness | -0.018 | 0.055 | 0.096 | 0.044 | 1.000E+00 | 1.000E+00 | 1.478E-03 | 1.000E+00 |
| F | SmallAreaLowGrayLevelEmphasis glszm MYO ED | Local Dimness | 0.081 | 0.061 | 0.042 | -0.055 | 1.000E+00 | 7.114E-02 | 1.000E+00 | 1.000E+00 |
| M | SmallAreaLowGrayLevelEmphasis glszm MYO ES | Local Dimness | 0.145 | 0.070 | 0.088 | -0.007 | 4.118E-02 | 2.361E-01 | 5.975E-03 | 1.000E+00 |
| F | SmallAreaLowGrayLevelEmphasis glszm MYO ES | Local Dimness | 0.163 | 0.095 | 0.100 | 0.019 | 9.896E-03 | 2.628E-05 | 9.713E-06 | 1.000E+00 |
| M | SmallDependenceLowGrayLevelEmphasis gldm MYO ED | Local Dimness | -0.038 | 0.063 | -0.009 | -0.025 | 1.000E+00 | 8.270E-02 | 1.000E+00 | 1.000E+00 |
| F | SmallDependenceLowGrayLevelEmphasis gldm MYO ED | Local Dimness | 0.030 | 0.041 | -0.132 | -0.187 | 1.000E+00 | 1.000E+00 | 4.789E-08 | 4.534E-02 |
| M | SmallDependenceLowGrayLevelEmphasis gldm MYO ES | Local Dimness | 0.132 | 0.076 | -0.028 | -0.028 | 3.745E-02 | 1.922E-02 | 1.000E+00 | 1.000E+00 |
| F | SmallDependenceLowGrayLevelEmphasis gldm MYO ES | Local Dimness | 0.153 | 0.080 | -0.042 | -0.052 | 9.243E-02 | 8.744E-03 | 1.000E+00 | 1.000E+00 |
| M | Correlation glcm MYO ES | Local Uniformity | -0.359 | -0.114 | -0.006 | -0.181 | 1.238E-21 | 1.195E-06 | 1.000E+00 | 3.217E-03 |
| F | Correlation glcm MYO ES | Local Uniformity | -0.217 | -0.042 | 0.020 | -0.009 | 2.146E-06 | 1.000E+00 | 1.000E+00 | 1.000E+00 |
| M | DependenceEntropy gldm MYO ES | Local Uniformity | -0.377 | -0.123 | -0.066 | -0.175 | 9.631E-20 | 2.197E-06 | 3.987E-01 | 2.844E-02 |
| F | DependenceEntropy gldm MYO ES | Local Uniformity | -0.296 | -0.095 | -0.019 | -0.070 | 5.042E-12 | 1.042E-05 | 1.000E+00 | 1.000E+00 |
| M | DependenceNonUniformity gldm MYO ES | Local Uniformity | -0.289 | -0.102 | 0.204 | 0.039 | 1.268E-18 | 3.570E-07 | 3.141E-32 | 1.000E+00 |
| F | DependenceNonUniformity gldm MYO ES | Local Uniformity | -0.110 | -0.046 | 0.175 | 0.080 | 4.200E-03 | 1.538E-02 | 2.022E-48 | 1.000E+00 |
| M | DependenceVariance gldm MYO ED | Local Uniformity | -0.016 | -0.016 | 0.127 | 0.026 | 1.000E+00 | 1.000E+00 | 2.388E-10 | 1.000E+00 |
| F | DependenceVariance gldm MYO ED | Local Uniformity | 0.020 | 0.032 | 0.165 | 0.052 | 1.000E+00 | 1.000E+00 | 2.694E-26 | 1.000E+00 |
| M | DependenceVariance gldm MYO ES | Local Uniformity | 0.126 | 0.062 | 0.121 | 0.066 | 4.517E-02 | 2.041E-01 | 1.006E-08 | 1.000E+00 |
| F | DependenceVariance gldm MYO ES | Local Uniformity | 0.115 | 0.028 | 0.162 | 0.107 | 1.000E+00 | 1.000E+00 | 6.590E-16 | 1.000E+00 |
| M | GrayLevelNonUniformity gldm MYO ED | Local Uniformity | -0.149 | -0.070 | 0.187 | 0.037 | 2.483E-04 | 7.983E-03 | 3.462E-27 | 1.000E+00 |
| F | GrayLevelNonUniformity gldm MYO ED | Local Uniformity | -0.012 | -0.010 | 0.157 | 0.084 | 1.000E+00 | 1.000E+00 | 2.818E-62 | 3.920E-02 |
| M | GrayLevelNonUniformity gldm MYO ES | Local Uniformity | 0.018 | 0.007 | 0.267 | 0.155 | 1.000E+00 | 1.000E+00 | 1.161E-51 | 7.116E-03 |
| F | GrayLevelNonUniformity gldm MYO ES | Local Uniformity | 0.074 | 0.014 | 0.225 | 0.133 | 4.089E-01 | 1.000E+00 | 5.080E-94 | 6.663E-05 |
| M | GrayLevelNonUniformity glrlm MYO ED | Local Uniformity | -0.204 | -0.073 | 0.163 | 0.037 | 5.195E-11 | 3.482E-04 | 5.319E-25 | 1.000E+00 |
| F | GrayLevelNonUniformity glrlm MYO ED | Local Uniformity | -0.041 | -0.026 | 0.180 | 0.097 | 1.000E+00 | 1.000E+00 | 3.413E-68 | 1.631E-02 |
| M | GrayLevelNonUniformity glrlm MYO ES | Local Uniformity | -0.090 | -0.020 | 0.229 | 0.153 | 5.461E-01 | 1.000E+00 | 9.246E-44 | 2.268E-03 |
| F | GrayLevelNonUniformity glrlm MYO ES | Local Uniformity | 0.032 | -0.007 | 0.232 | 0.131 | 1.000E+00 | 1.000E+00 | 1.106E-90 | 3.662E-04 |
| M | GrayLevelNonUniformityNormalized glrlm MYO ED | Local Uniformity | 0.053 | -0.015 | 0.085 | 0.025 | 1.000E+00 | 1.000E+00 | 1.945E-04 | 1.000E+00 |
| F | GrayLevelNonUniformityNormalized glrlm MYO ED | Local Uniformity | 0.112 | 0.037 | 0.156 | 0.098 | 2.502E-01 | 1.000E+00 | 2.232E-21 | 1.000E+00 |
| M | GrayLevelNonUniformityNormalized glrlm MYO ES | Local Uniformity | 0.281 | 0.108 | 0.132 | 0.192 | 1.358E-11 | 3.397E-05 | 2.015E-08 | 2.974E-03 |
| F | GrayLevelNonUniformityNormalized glrlm MYO ES | Local Uniformity | 0.285 | 0.106 | 0.157 | 0.120 | 7.465E-11 | 4.049E-07 | 2.118E-16 | 1.000E+00 |
| M | GrayLevelNonUniformityNormalized glszm MYO ED | Local Uniformity | 0.040 | -0.004 | 0.041 | 0.004 | 1.000E+00 | 1.000E+00 | 1.000E+00 | 1.000E+00 |
| F | GrayLevelNonUniformityNormalized glszm MYO ED | Local Uniformity | 0.072 | 0.026 | 0.112 | 0.021 | 1.000E+00 | 1.000E+00 | 8.901E-11 | 1.000E+00 |
| M | GrayLevelNonUniformityNormalized glszm MYO ES | Local Uniformity | 0.334 | 0.117 | 0.137 | 0.153 | 1.436E-15 | 9.581E-06 | 1.426E-08 | 1.717E-01 |
| F | GrayLevelNonUniformityNormalized glszm MYO ES | Local Uniformity | 0.217 | 0.121 | 0.131 | 0.136 | 2.439E-06 | 3.714E-10 | 8.160E-12 | 2.530E-01 |
| M | InformalMeasureofCorrelation2 glcm MYO ES | Local Uniformity | -0.399 | -0.126 | -0.037 | -0.211 | 2.835E-24 | 1.879E-07 | 1.000E+00 | 3.467E-04 |
| F | InformalMeasureofCorrelation2 glcm MYO ES | Local Uniformity | -0.245 | -0.058 | -0.017 | -0.051 | 2.311E-08 | 1.449E-01 | 1.000E+00 | 1.000E+00 |
| M | InverseDifference glcm MYO ED | Local Uniformity | 0.017 | -0.014 | 0.111 | 0.029 | 1.000E+00 | 1.000E+00 | 7.617E-10 | 1.000E+00 |
| F | InverseDifference glcm MYO ED | Local Uniformity | 0.075 | 0.039 | 0.195 | 0.099 | 1.000E+00 | 1.000E+00 | 2.838E-33 | 1.000E+00 |
| M | InverseDifference glcm MYO ES | Local Uniformity | 0.083 | 0.041 | 0.116 | 0.022 | 1.000E+00 | 1.000E+00 | 3.765E-08 | 1.000E+00 |
| F | InverseDifference glcm MYO ES | Local Uniformity | 0.154 | 0.063 | 0.159 | 0.105 | 1.794E-02 | 7.180E-02 | 5.098E-17 | 1.000E+00 |
| M | InverseDifferenceMoment glcm MYO ED | Local Uniformity | 0.021 | -0.013 | 0.110 | 0.032 | 1.000E+00 | 1.000E+00 | 2.429E-10 | 1.000E+00 |
| F | InverseDifferenceMoment glcm MYO ED | Local Uniformity | 0.077 | 0.041 | 0.204 | 0.105 | 1.000E+00 | 1.000E+00 | 2.302E-35 | 1.000E+00 |
| M | InverseDifferenceMoment glcm MYO ES | Local Uniformity | 0.081 | 0.040 | 0.117 | 0.021 | 1.000E+00 | 1.000E+00 | 2.042E-08 | 1.000E+00 |
| F | InverseDifferenceMoment glcm MYO ES | Local Uniformity | 0.153 | 0.064 | 0.162 | 0.107 | 2.230E-02 | 7.668E-02 | 2.592E-17 | 1.000E+00 |
| M | InverseDifferenceMomentNormalized glcm MYO ED | Local Uniformity | -0.043 | -0.052 | 0.081 | 0.039 | 1.000E+00 | 7.492E-01 | 6.706E-04 | 1.000E+00 |
| F | InverseDifferenceMomentNormalized glcm MYO ED | Local Uniformity | -0.040 | -0.004 | 0.164 | 0.103 | 1.000E+00 | 1.000E+00 | 3.074E-14 | 1.000E+00 |
| M | InverseDifferenceMomentNormalized glcm MYO ES | Local Uniformity | -0.099 | -0.029 | 0.008 | 0.002 | 9.251E-01 | 1.000E+00 | 1.000E+00 | 1.000E+00 |
| F | InverseDifferenceMomentNormalized glcm MYO ES | Local Uniformity | -0.046 | -0.068 | -0.002 | -0.021 | 1.000E+00 | 1.641E-01 | 1.000E+00 | 1.000E+00 |
| M | InverseDifferenceNormalized glcm MYO ED | Local Uniformity | -0.046 | -0.049 | 0.109 | 0.032 | 1.000E+00 | 1.000E+00 | 6.360E-08 | 1.000E+00 |
| F | InverseDifferenceNormalized glcm MYO ED | Local Uniformity | -0.014 | 0.005 | 0.175 | 0.089 | 1.000E+00 | 1.000E+00 | 1.740E-20 | 1.000E+00 |
| M | InverseDifferenceNormalized glcm MYO ES | Local Uniformity | -0.025 | 0.000 | 0.061 | 0.013 | 1.000E+00 | 1.000E+00 | 2.309E-01 | 1.000E+00 |
| F | InverseDifferenceNormalized glcm MYO ES | Local Uniformity | 0.037 | -0.017 | 0.073 | 0.026 | 1.000E+00 | 1.000E+00 | 2.866E-02 | 1.000E+00 |
| M | JointEnergy glcm MYO ED | Local Uniformity | 0.042 | -0.011 | 0.077 | 0.004 | 1.000E+00 | 1.000E+00 | 2.490E-02 | 1.000E+00 |
| F | JointEnergy glcm MYO ED | Local Uniformity | 0.082 | 0.037 | 0.102 | 0.061 | 1.000E+00 | 1.000E+00 | 4.583E-11 | 1.000E+00 |
| M | JointEnergy glcm MYO ES | Local Uniformity | 0.285 | 0.102 | 0.112 | 0.129 | 5.102E-11 | 3.724E-04 | 2.302E-05 | 1.000E+00 |
| F | JointEnergy glcm MYO ES | Local Uniformity | 0.227 | 0.082 | 0.123 | 0.118 | 1.507E-07 | 1.881E-04 | 3.686E-11 | 8.340E-01 |
| M | LargeAreaEmphasis glszm MYO ED | Local Uniformity | -0.050 | -0.060 | 0.206 | 0.093 | 1.000E+00 | 8.985E-01 | 1.902E-21 | 1.000E+00 |
| F | LargeAreaEmphasis glszm MYO ED | Local Uniformity | 0.026 | 0.002 | 0.118 | 0.070 | 1.000E+00 | 1.000E+00 | 7.199E-52 | 2.992E-02 |
| M | LargeAreaEmphasis glszm MYO ES | Local Uniformity | 0.026 | -0.036 | 0.300 | 0.147 | 1.000E+00 | 1.000E+00 | 1.544E-45 | 2.208E-01 |
| F | LargeAreaEmphasis glszm MYO ES | Local Uniformity | 0.059 | 0.023 | 0.191 | 0.098 | 1.000E+00 | 1.000E+00 | 6.996E-78 | 1.237E-02 |
| M | LargeAreaHighGrayLevelEmphasis glszm MYO ED | Local Uniformity | -0.047 | -0.076 | 0.194 | 0.094 | 1.000E+00 | 9.023E-02 | 5.762E-18 | 1.000E+00 |
| F | LargeAreaHighGrayLevelEmphasis glszm MYO ED | Local Uniformity | 0.000 | -0.013 | 0.145 | 0.124 | 1.000E+00 | 1.000E+00 | 1.718E-66 | 9.691E-08 |
| M | LargeAreaHighGrayLevelEmphasis glszm MYO ES | Local Uniformity | -0.039 | -0.053 | 0.227 | 0.094 | 1.000E+00 | 1.000E+00 | 2.740E-23 | 1.000E+00 |
| F | LargeAreaHighGrayLevelEmphasis glszm MYO ES | Local Uniformity | -0.033 | -0.024 | 0.158 | 0.114 | 1.000E+00 | 1.000E+00 | 2.162E-47 | 1.947E-03 |
| M | LargeDependenceEmphasis gldm MYO ED | Local Uniformity | -0.007 | -0.033 | 0.168 | 0.048 | 1.000E+00 | 1.000E+00 | 4.477E-18 | 1.000E+00 |
| F | LargeDependenceEmphasis gldm MYO ED | Local Uniformity | 0.033 | 0.023 | 0.176 | 0.082 | 1.000E+00 | 1.000E+00 | 2.927E-40 | 1.000E+00 |
| M | LargeDependenceEmphasis gldm MYO ES | Local Uniformity | 0.057 | 0.024 | 0.185 | 0.060 | 1.000E+00 | 1.000E+00 | 6.387E-20 | 1.000E+00 |
| F | LargeDependenceEmphasis gldm MYO ES | Local Uniformity | 0.091 | 0.047 | 0.204 | 0.110 | 1.000E+00 | 5.788E-01 | 6.780E-36 | 9.277E-01 |
| M | LargeDependenceHighGrayLevelEmphasis gldm MYO ED | Local Uniformity | 0.040 | -0.053 | 0.117 | 0.071 | 1.000E+00 | 1.000E+00 | 4.175E-06 | 1.000E+00 |
| F | LargeDependenceHighGrayLevelEmphasis gldm MYO ED | Local Uniformity | -0.014 | -0.019 | 0.191 | 0.188 | 1.000E+00 | 1.000E+00 | 1.760E-33 | 7.926E-05 |
| M | LargeDependenceHighGrayLevelEmphasis gldm MYO ES | Local Uniformity | -0.033 | -0.029 | 0.025 | -0.011 | 1.000E+00 | 1.000E+00 | 1.000E+00 | 1.000E+00 |
| F | LargeDependenceHighGrayLevelEmphasis gldm MYO ES | Local Uniformity | -0.104 | -0.075 | 0.040 | 0.084 | 1.000E+00 | 3.368E-03 | 1.000E+00 | 1.000E+00 |
| M | LongRunEmphasis glrlm MYO ED | Local Uniformity | -0.003 | -0.032 | 0.203 | 0.075 | 1.000E+00 | 1.000E+00 | 3.967E-23 | 1.000E+00 |
| F | LongRunEmphasis glrlm MYO ED | Local Uniformity | 0.051 | 0.023 | 0.160 | 0.092 | 1.000E+00 | 1.000E+00 | 6.209E-47 | 9.508E-02 |
| M | LongRunEmphasis glrlm MYO ES | Local Uniformity | 0.040 | 0.023 | 0.226 | 0.076 | 1.000E+00 | 1.000E+00 | 1.274E-26 | 1.000E+00 |
| F | LongRunEmphasis glrlm MYO ES | Local Uniformity | 0.099 | 0.053 | 0.187 | 0.119 | 2.031E-01 | 1.649E-02 | 2.666E-41 | 5.943E-02 |
| M | LongRunHighGrayLevelEmphasis glrlm MYO ED | Local Uniformity | 0.030 | -0.059 | 0.125 | 0.076 | 1.000E+00 | 1.000E+00 | 9.212E-07 | 1.000E+00 |
| F | LongRunHighGrayLevelEmphasis glrlm MYO ED | Local Uniformity | -0.008 | -0.025 | 0.151 | 0.203 | 1.000E+00 | 1.000E+00 | 8.818E-19 | 3.507E-05 |
| M | LongRunHighGrayLevelEmphasis glrlm MYO ES | Local Uniformity | -0.043 | -0.024 | 0.083 | 0.016 | 1.000E+00 | 1.000E+00 | 2.762E-02 | 1.000E+00 |
| F | LongRunHighGrayLevelEmphasis glrlm MYO ES | Local Uniformity | -0.070 | -0.055 | 0.066 | 0.111 | 1.000E+00 | 1.045E-01 | 7.997E-03 | 9.069E-01 |
| M | MaximumProbability glcm MYO ED | Local Uniformity | 0.057 | 0.005 | 0.064 | -0.007 | 1.000E+00 | 1.000E+00 | 1.741E-01 | 1.000E+00 |
| F | MaximumProbability glcm MYO ED | Local Uniformity | 0.086 | 0.047 | 0.090 | 0.052 | 1.000E+00 | 4.679E-01 | 1.483E-06 | 1.000E+00 |
| M | MaximumProbability glcm MYO ES | Local Uniformity | 0.276 | 0.096 | 0.092 | 0.108 | 7.543E-11 | 9.905E-04 | 1.519E-03 | 1.000E+00 |
| F | MaximumProbability glcm MYO ES | Local Uniformity | 0.208 | 0.070 | 0.105 | 0.100 | 2.013E-05 | 1.395E-02 | 6.652E-07 | 1.000E+00 |
| M | RunEntropy glrlm MYO ES | Local Uniformity | -0.272 | -0.095 | 0.070 | -0.121 | 1.007E-11 | 3.502E-04 | 7.832E-02 | 9.858E-01 |
| F | RunEntropy glrlm MYO ES | Local Uniformity | -0.224 | -0.063 | 0.067 | -0.001 | 6.205E-07 | 4.738E-02 | 2.121E-02 | 1.000E+00 |
| M | RunVariance glrlm MYO ED | Local Uniformity | -0.011 | -0.031 | 0.203 | 0.068 | 1.000E+00 | 1.000E+00 | 6.543E-23 | 1.000E+00 |
| F | RunVariance glrlm MYO ED | Local Uniformity | 0.047 | 0.023 | 0.152 | 0.087 | 1.000E+00 | 1.000E+00 | 1.990E-43 | 1.625E-01 |
| M | RunVariance glrlm MYO ES | Local Uniformity | 0.047 | 0.028 | 0.244 | 0.078 | 1.000E+00 | 1.000E+00 | 1.587E-31 | 1.000E+00 |
| F | RunVariance glrlm MYO ES | Local Uniformity | 0.100 | 0.052 | 0.196 | 0.123 | 1.382E-01 | 1.431E-02 | 2.650E-47 | 2.637E-02 |
| M | Uniformity MYO ED | Local Uniformity | 0.055 | -0.008 | 0.083 | 0.008 | 1.000E+00 | 1.000E+00 | 1.191E-03 | 1.000E+00 |
| F | Uniformity MYO ED | Local Uniformity | 0.101 | 0.040 | 0.133 | 0.085 | 4.062E-01 | 1.000E+00 | 3.563E-17 | 1.000E+00 |
| M | Uniformity MYO ES | Local Uniformity | 0.335 | 0.122 | 0.123 | 0.174 | 1.243E-15 | 2.381E-06 | 9.979E-07 | 2.875E-02 |
| F | Uniformity MYO ES | Local Uniformity | 0.267 | 0.094 | 0.142 | 0.131 | 1.865E-10 | 5.350E-06 | 1.115E-14 | 3.328E-01 |
| M | ZoneVariance glszm MYO ED | Local Uniformity | -0.054 | -0.061 | 0.207 | 0.091 | 1.000E+00 | 7.628E-01 | 1.060E-21 | 1.000E+00 |
| F | ZoneVariance glszm MYO ED | Local Uniformity | 0.025 | 0.001 | 0.118 | 0.070 | 1.000E+00 | 1.000E+00 | 4.429E-52 | 2.928E-02 |
| M | ZoneVariance glszm MYO ES | Local Uniformity | 0.025 | -0.035 | 0.301 | 0.149 | 1.000E+00 | 1.000E+00 | 4.027E-46 | 1.823E-01 |
| F | ZoneVariance glszm MYO ES | Local Uniformity | 0.061 | 0.023 | 0.192 | 0.099 | 1.000E+00 | 1.000E+00 | 4.106E-80 | 7.678E-03 |
| M | ClusterProminence glcm MYO ED | Shape | -0.044 | 0.022 | 0.009 | -0.011 | 1.000E+00 | 1.000E+00 | 1.000E+00 | 1.000E+00 |
| F | ClusterProminence glcm MYO ED | Shape | -0.044 | 0.025 | -0.079 | 0.045 | 1.000E+00 | 1.000E+00 | 1.118E-01 | 1.000E+00 |
| M | ClusterProminence glcm MYO ES | Shape | -0.037 | 0.004 | -0.004 | -0.002 | 1.000E+00 | 1.000E+00 | 1.000E+00 | 1.000E+00 |
| F | ClusterProminence glcm MYO ES | Shape | -0.071 | 0.030 | -0.006 | 0.140 | 1.000E+00 | 1.000E+00 | 1.000E+00 | 1.000E+00 |
| M | ClusterShade glcm MYO ED | Shape | -0.068 | 0.018 | -0.005 | -0.022 | 1.000E+00 | 1.000E+00 | 1.000E+00 | 1.000E+00 |
| F | ClusterShade glcm MYO ED | Shape | -0.074 | 0.021 | -0.125 | -0.029 | 1.000E+00 | 1.000E+00 | 1.427E-06 | 1.000E+00 |
| M | ClusterShade glcm MYO ES | Shape | -0.054 | 0.008 | 0.000 | 0.017 | 1.000E+00 | 1.000E+00 | 1.000E+00 | 1.000E+00 |
| F | ClusterShade glcm MYO ES | Shape | -0.069 | 0.037 | 0.004 | 0.126 | 1.000E+00 | 1.000E+00 | 1.000E+00 | 1.000E+00 |
| M | Coarseness ngtdm MYO ED | Shape | 0.237 | 0.077 | -0.106 | 0.014 | 1.849E-17 | 1.701E-05 | 1.767E-11 | 1.000E+00 |
| F | Coarseness ngtdm MYO ED | Shape | 0.205 | 0.082 | -0.188 | -0.070 | 7.766E-07 | 3.275E-05 | 2.999E-30 | 1.000E+00 |
| M | Coarseness ngtdm MYO ES | Shape | 0.163 | 0.060 | -0.121 | -0.056 | 4.929E-06 | 3.522E-02 | 3.113E-12 | 1.000E+00 |
| F | Coarseness ngtdm MYO ES | Shape | 0.115 | 0.067 | -0.218 | -0.064 | 8.096E-01 | 3.334E-02 | 1.001E-31 | 1.000E+00 |
| M | Complexity ngtdm MYO ES | Shape | -0.121 | -0.041 | -0.081 | -0.003 | 3.924E-02 | 1.000E+00 | 7.011E-04 | 1.000E+00 |
| F | Complexity ngtdm MYO ES | Shape | -0.182 | -0.090 | -0.112 | -0.134 | 1.483E-02 | 2.871E-03 | 1.457E-05 | 1.000E+00 |
| M | Elongation LV ED | Shape | 0.007 | -0.036 | -0.069 | -0.035 | 1.000E+00 | 1.000E+00 | 6.158E-02 | 1.000E+00 |
| F | Elongation LV ED | Shape | 0.015 | -0.055 | -0.039 | -0.042 | 1.000E+00 | 1.000E+00 | 1.000E+00 | 1.000E+00 |
| M | Elongation LV ES | Shape | 0.024 | -0.023 | -0.100 | 0.009 | 1.000E+00 | 1.000E+00 | 3.817E-05 | 1.000E+00 |
| F | Elongation LV ES | Shape | -0.020 | -0.040 | -0.110 | -0.028 | 1.000E+00 | 1.000E+00 | 1.076E-05 | 1.000E+00 |
| M | Elongation RV ED | Shape | -0.160 | -0.017 | 0.002 | -0.079 | 1.457E-03 | 1.000E+00 | 1.000E+00 | 1.000E+00 |
| F | Elongation RV ED | Shape | -0.082 | 0.011 | 0.034 | -0.203 | 1.000E+00 | 1.000E+00 | 1.000E+00 | 7.055E-03 |
| M | Elongation RV ES | Shape | -0.162 | -0.018 | -0.040 | -0.106 | 8.809E-04 | 1.000E+00 | 1.000E+00 | 1.000E+00 |
| F | Elongation RV ES | Shape | -0.119 | -0.022 | -0.004 | -0.209 | 1.000E+00 | 1.000E+00 | 1.000E+00 | 2.255E-03 |
| M | Flatness LV ED | Shape | -0.040 | -0.056 | -0.075 | -0.012 | 1.000E+00 | 9.293E-01 | 2.270E-02 | 1.000E+00 |
| F | Flatness LV ED | Shape | -0.048 | -0.059 | -0.007 | -0.086 | 1.000E+00 | 6.100E-01 | 1.000E+00 | 1.000E+00 |
| M | Flatness LV ES | Shape | 0.008 | -0.061 | -0.170 | 0.020 | 1.000E+00 | 3.085E-01 | 4.943E-17 | 1.000E+00 |
| F | Flatness LV ES | Shape | -0.051 | -0.083 | -0.171 | -0.068 | 1.000E+00 | 8.025E-03 | 4.039E-15 | 1.000E+00 |
| M | Flatness RV ED | Shape | 0.002 | 0.071 | 0.094 | -0.117 | 1.000E+00 | 6.333E-02 | 2.350E-04 | 1.000E+00 |
| F | Flatness RV ED | Shape | -0.015 | 0.076 | 0.110 | -0.158 | 1.000E+00 | 1.559E-02 | 2.958E-06 | 1.705E-01 |
| M | Flatness RV ES | Shape | -0.103 | 0.038 | -0.041 | -0.117 | 1.000E+00 | 1.000E+00 | 1.000E+00 | 1.000E+00 |
| F | Flatness RV ES | Shape | -0.144 | -0.055 | -0.021 | -0.209 | 1.737E-01 | 9.174E-01 | 1.000E+00 | 1.884E-03 |
| M | GrayLevelVariance glszm MYO ES | Shape | -0.115 | -0.055 | -0.105 | -0.048 | 7.023E-02 | 3.854E-01 | 3.235E-07 | 1.000E+00 |
| F | GrayLevelVariance glszm MYO ES | Shape | -0.198 | -0.114 | -0.157 | -0.151 | 1.845E-03 | 3.061E-06 | 1.800E-12 | 4.707E-01 |
| M | InformalMeasureofCorrelation1 glcm MYO ED | Shape | 0.069 | 0.008 | -0.171 | -0.136 | 1.000E+00 | 1.000E+00 | 3.443E-13 | 7.933E-01 |
| F | InformalMeasureofCorrelation1 glcm MYO ED | Shape | -0.049 | -0.008 | -0.142 | -0.136 | 1.000E+00 | 1.000E+00 | 1.250E-16 | 9.704E-02 |
| M | InformalMeasureofCorrelation1 glcm MYO ES | Shape | 0.368 | 0.123 | -0.006 | 0.197 | 3.744E-20 | 6.334E-07 | 1.000E+00 | 1.988E-03 |
| F | InformalMeasureofCorrelation1 glcm MYO ES | Shape | 0.170 | 0.033 | -0.032 | -0.003 | 9.334E-05 | 1.000E+00 | 1.000E+00 | 1.000E+00 |
| M | Kurtosis MYO ED | Shape | -0.084 | -0.020 | 0.054 | 0.011 | 1.000E+00 | 1.000E+00 | 1.000E+00 | 1.000E+00 |
| F | Kurtosis MYO ED | Shape | 0.009 | 0.015 | 0.042 | -0.066 | 1.000E+00 | 1.000E+00 | 1.000E+00 | 1.000E+00 |
| M | Kurtosis MYO ES | Shape | 0.071 | 0.018 | -0.006 | 0.108 | 1.000E+00 | 1.000E+00 | 1.000E+00 | 1.000E+00 |
| F | Kurtosis MYO ES | Shape | 0.076 | -0.007 | 0.004 | -0.048 | 1.000E+00 | 1.000E+00 | 1.000E+00 | 1.000E+00 |
| M | Maximum MYO ES | Shape | -0.210 | -0.101 | -0.081 | -0.073 | 4.238E-07 | 2.810E-05 | 4.312E-03 | 1.000E+00 |
| F | Maximum MYO ES | Shape | -0.209 | -0.115 | -0.110 | -0.174 | 1.937E-04 | 4.134E-07 | 2.512E-06 | 4.526E-02 |
| M | Minimum MYO ED | Shape | -0.195 | -0.049 | 0.002 | -0.120 | 9.627E-05 | 1.000E+00 | 1.000E+00 | 1.000E+00 |
| F | Minimum MYO ED | Shape | -0.119 | -0.021 | 0.025 | -0.191 | 7.248E-01 | 1.000E+00 | 1.000E+00 | 3.855E-03 |
| M | Minimum MYO ES | Shape | -0.208 | -0.098 | 0.018 | -0.128 | 2.598E-05 | 1.138E-03 | 1.000E+00 | 1.000E+00 |
| F | Minimum MYO ES | Shape | -0.127 | -0.014 | 0.029 | -0.128 | 3.080E-01 | 1.000E+00 | 1.000E+00 | 7.273E-01 |
| M | Range MYO ES | Shape | -0.163 | -0.079 | -0.094 | -0.039 | 4.420E-04 | 5.466E-03 | 9.335E-05 | 1.000E+00 |
| F | Range MYO ES | Shape | -0.187 | -0.121 | -0.129 | -0.149 | 3.502E-03 | 1.513E-07 | 1.324E-08 | 4.196E-01 |
| M | SizeZoneNonUniformityNormalized glszm MYO ED | Shape | -0.065 | -0.019 | 0.045 | 0.041 | 1.000E+00 | 1.000E+00 | 1.000E+00 | 1.000E+00 |
| F | SizeZoneNonUniformityNormalized glszm MYO ED | Shape | -0.083 | -0.032 | -0.031 | -0.028 | 1.000E+00 | 1.000E+00 | 1.000E+00 | 1.000E+00 |
| M | SizeZoneNonUniformityNormalized glszm MYO ES | Shape | -0.080 | -0.017 | 0.011 | -0.033 | 1.000E+00 | 1.000E+00 | 1.000E+00 | 1.000E+00 |
| F | SizeZoneNonUniformityNormalized glszm MYO ES | Shape | 0.125 | -0.037 | -0.040 | -0.133 | 1.000E+00 | 1.000E+00 | 1.000E+00 | 1.000E+00 |
| M | Skewness MYO ED | Shape | -0.110 | -0.015 | -0.009 | -0.053 | 1.000E+00 | 1.000E+00 | 1.000E+00 | 1.000E+00 |
| F | Skewness MYO ED | Shape | -0.021 | 0.020 | -0.022 | -0.167 | 1.000E+00 | 1.000E+00 | 1.000E+00 | 1.049E-02 |
| M | Skewness MYO ES | Shape | -0.019 | 0.011 | 0.010 | 0.049 | 1.000E+00 | 1.000E+00 | 1.000E+00 | 1.000E+00 |
| F | Skewness MYO ES | Shape | 0.011 | 0.026 | 0.008 | -0.101 | 1.000E+00 | 1.000E+00 | 1.000E+00 | 1.000E+00 |
| M | SmallAreaEmphasis glszm MYO ED | Shape | -0.071 | -0.019 | 0.040 | 0.042 | 1.000E+00 | 1.000E+00 | 1.000E+00 | 1.000E+00 |
| F | SmallAreaEmphasis glszm MYO ED | Shape | -0.087 | -0.034 | -0.030 | -0.034 | 1.000E+00 | 1.000E+00 | 1.000E+00 | 1.000E+00 |
| M | SmallAreaEmphasis glszm MYO ES | Shape | -0.093 | -0.012 | 0.010 | -0.044 | 1.000E+00 | 1.000E+00 | 1.000E+00 | 1.000E+00 |
| F | SmallAreaEmphasis glszm MYO ES | Shape | 0.124 | -0.042 | -0.045 | -0.139 | 1.000E+00 | 1.000E+00 | 1.000E+00 | 1.000E+00 |
| M | Sphericity LV ED | Shape | -0.026 | -0.116 | -0.145 | -0.067 | 1.000E+00 | 6.082E-06 | 4.198E-10 | 1.000E+00 |
| F | Sphericity LV ED | Shape | -0.216 | -0.111 | -0.143 | -0.163 | 1.256E-06 | 5.973E-09 | 5.153E-15 | 1.475E-02 |
| M | Sphericity LV ES | Shape | 0.030 | -0.121 | -0.189 | -0.001 | 1.000E+00 | 1.888E-07 | 4.804E-20 | 1.000E+00 |
| F | Sphericity LV ES | Shape | -0.156 | -0.110 | -0.209 | -0.092 | 1.406E-02 | 1.006E-07 | 1.764E-29 | 1.000E+00 |
| M | Sphericity RV ED | Shape | 0.077 | 0.112 | 0.055 | -0.123 | 1.000E+00 | 4.984E-06 | 1.000E+00 | 9.419E-01 |
| F | Sphericity RV ED | Shape | 0.056 | 0.128 | 0.031 | -0.095 | 1.000E+00 | 7.400E-09 | 1.000E+00 | 1.000E+00 |
| M | Sphericity RV ES | Shape | 0.052 | 0.078 | -0.020 | -0.049 | 1.000E+00 | 2.101E-02 | 1.000E+00 | 1.000E+00 |
| F | Sphericity RV ES | Shape | -0.090 | 0.041 | -0.041 | -0.172 | 1.000E+00 | 1.000E+00 | 1.000E+00 | 6.499E-02 |
| M | Strength ngtdm MYO ES | Shape | -0.044 | -0.017 | -0.081 | -0.033 | 1.000E+00 | 1.000E+00 | 2.097E-05 | 1.000E+00 |
| F | Strength ngtdm MYO ES | Shape | -0.066 | -0.058 | -0.138 | -0.142 | 1.000E+00 | 1.000E+00 | 5.858E-08 | 1.000E+00 |
| M | SurfaceAreatoVolumeRatio LV ED | Shape | 0.313 | 0.178 | 0.081 | 0.076 | 5.936E-19 | 3.085E-20 | 1.236E-03 | 1.000E+00 |
| F | SurfaceAreatoVolumeRatio LV ED | Shape | 0.387 | 0.182 | 0.058 | 0.137 | 1.280E-25 | 9.202E-28 | 6.824E-02 | 9.564E-02 |
| M | SurfaceAreatoVolumeRatio LV ES | Shape | 0.190 | 0.162 | 0.147 | 0.025 | 5.063E-07 | 2.893E-18 | 2.622E-15 | 1.000E+00 |
| F | SurfaceAreatoVolumeRatio LV ES | Shape | 0.256 | 0.176 | 0.141 | 0.062 | 1.433E-09 | 3.333E-23 | 2.767E-14 | 1.000E+00 |
| M | SurfaceAreatoVolumeRatio RV ED | Shape | 0.267 | 0.055 | 0.014 | 0.160 | 1.300E-21 | 3.372E-02 | 1.000E+00 | 5.107E-05 |
| F | SurfaceAreatoVolumeRatio RV ED | Shape | 0.179 | 0.051 | 0.011 | 0.158 | 2.439E-05 | 1.969E-01 | 1.000E+00 | 4.903E-03 |
| M | SurfaceAreatoVolumeRatio RV ES | Shape | 0.176 | 0.059 | 0.088 | 0.064 | 9.319E-10 | 4.771E-03 | 3.017E-08 | 1.000E+00 |
| F | SurfaceAreatoVolumeRatio RV ES | Shape | 0.173 | 0.092 | 0.090 | 0.148 | 3.945E-04 | 4.130E-06 | 9.818E-06 | 4.578E-02 |
| M | DependenceNonUniformity gldm MYO ED | Size | -0.301 | -0.079 | 0.103 | 0.037 | 3.191E-15 | 1.091E-02 | 1.666E-05 | 1.000E+00 |
| F | DependenceNonUniformity gldm MYO ED | Size | -0.159 | -0.087 | 0.064 | 0.066 | 7.638E-04 | 3.702E-06 | 1.024E-02 | 1.000E+00 |
| M | GrayLevelNonUniformity glszm MYO ED | Size | -0.319 | -0.071 | 0.024 | -0.108 | 4.862E-14 | 2.171E-01 | 1.000E+00 | 1.000E+00 |
| F | GrayLevelNonUniformity glszm MYO ED | Size | -0.163 | -0.080 | -0.009 | -0.071 | 7.999E-04 | 1.156E-04 | 1.000E+00 | 1.000E+00 |
| M | GrayLevelNonUniformity glszm MYO ES | Size | 0.079 | 0.072 | 0.072 | 0.069 | 1.000E+00 | 1.387E-01 | 1.087E-01 | 1.000E+00 |
| F | GrayLevelNonUniformity glszm MYO ES | Size | 0.078 | 0.003 | 0.054 | 0.093 | 1.000E+00 | 1.000E+00 | 2.059E-01 | 1.000E+00 |
| M | LeastAxis LV ED | Size | -0.335 | -0.140 | -0.021 | -0.052 | 1.481E-24 | 6.948E-14 | 1.000E+00 | 1.000E+00 |
| F | LeastAxis LV ED | Size | -0.245 | -0.117 | 0.047 | -0.078 | 4.813E-13 | 7.941E-15 | 1.830E-01 | 1.000E+00 |
| M | LeastAxis LV ES | Size | -0.204 | -0.119 | -0.096 | -0.032 | 1.386E-08 | 8.336E-10 | 3.840E-06 | 1.000E+00 |
| F | LeastAxis LV ES | Size | -0.134 | -0.117 | -0.052 | -0.024 | 5.883E-03 | 8.824E-14 | 7.224E-02 | 1.000E+00 |
| M | LeastAxis RV ED | Size | -0.284 | -0.088 | 0.010 | -0.142 | 6.690E-20 | 1.166E-05 | 1.000E+00 | 8.209E-03 |
| F | LeastAxis RV ED | Size | -0.206 | -0.087 | 0.033 | -0.112 | 1.073E-08 | 1.542E-07 | 1.000E+00 | 2.538E-01 |
| M | LeastAxis RV ES | Size | -0.229 | -0.087 | -0.093 | -0.074 | 3.151E-13 | 8.117E-06 | 6.332E-07 | 1.000E+00 |
| F | LeastAxis RV ES | Size | -0.138 | -0.127 | -0.059 | -0.085 | 1.851E-03 | 2.567E-17 | 6.750E-03 | 1.000E+00 |
| M | MajorAxis LV ED | Size | -0.250 | -0.063 | 0.056 | -0.031 | 5.052E-15 | 2.554E-02 | 1.077E-01 | 1.000E+00 |
| F | MajorAxis LV ED | Size | -0.155 | -0.045 | 0.045 | 0.022 | 2.429E-05 | 1.573E-01 | 1.773E-01 | 1.000E+00 |
| M | MajorAxis LV ES | Size | -0.206 | -0.053 | 0.064 | -0.048 | 2.538E-10 | 2.028E-01 | 9.753E-03 | 1.000E+00 |
| F | MajorAxis LV ES | Size | -0.083 | -0.038 | 0.087 | 0.036 | 1.000E+00 | 1.000E+00 | 1.667E-07 | 1.000E+00 |
| M | MajorAxis RV ED | Size | -0.279 | -0.165 | -0.094 | -0.014 | 1.142E-17 | 1.494E-20 | 2.373E-06 | 1.000E+00 |
| F | MajorAxis RV ED | Size | -0.184 | -0.161 | -0.080 | 0.051 | 1.574E-06 | 7.400E-27 | 8.098E-06 | 1.000E+00 |
| M | MajorAxis RV ES | Size | -0.145 | -0.139 | -0.056 | 0.047 | 6.545E-04 | 6.314E-14 | 1.897E-01 | 1.000E+00 |
| F | MajorAxis RV ES | Size | 0.002 | -0.081 | -0.042 | 0.132 | 1.000E+00 | 3.662E-05 | 1.000E+00 | 1.160E-01 |
| M | Max2DdiameterColumn LV ED | Size | -0.275 | -0.090 | 0.032 | -0.040 | 3.261E-20 | 1.520E-06 | 1.000E+00 | 1.000E+00 |
| F | Max2DdiameterColumn LV ED | Size | -0.212 | -0.068 | 0.030 | 0.036 | 2.165E-10 | 9.481E-05 | 1.000E+00 | 1.000E+00 |
| M | Max2DdiameterColumn LV ES | Size | -0.206 | -0.088 | 0.038 | -0.042 | 6.271E-10 | 1.210E-05 | 1.000E+00 | 1.000E+00 |
| F | Max2DdiameterColumn LV ES | Size | -0.116 | -0.053 | 0.049 | 0.038 | 7.651E-02 | 5.570E-02 | 2.083E-01 | 1.000E+00 |
| M | Max2DdiameterColumn RV ED | Size | -0.316 | -0.124 | 0.024 | -0.130 | 1.606E-23 | 1.540E-11 | 1.000E+00 | 5.141E-02 |
| F | Max2DdiameterColumn RV ED | Size | -0.216 | -0.105 | 0.021 | -0.098 | 2.838E-09 | 6.656E-11 | 1.000E+00 | 1.000E+00 |
| M | Max2DdiameterColumn RV ES | Size | -0.238 | -0.098 | -0.025 | -0.095 | 5.416E-12 | 2.777E-06 | 1.000E+00 | 1.000E+00 |
| F | Max2DdiameterColumn RV ES | Size | -0.137 | -0.081 | -0.001 | -0.057 | 6.730E-03 | 1.107E-05 | 1.000E+00 | 1.000E+00 |
| M | Max2DdiameterRow LV ED | Size | -0.281 | -0.094 | 0.033 | -0.052 | 1.579E-20 | 4.478E-07 | 1.000E+00 | 1.000E+00 |
| F | Max2DdiameterRow LV ED | Size | -0.202 | -0.064 | 0.022 | 0.001 | 7.613E-08 | 2.151E-03 | 1.000E+00 | 1.000E+00 |
| M | Max2DdiameterRow LV ES | Size | -0.242 | -0.072 | 0.048 | -0.044 | 1.070E-14 | 1.286E-03 | 5.234E-01 | 1.000E+00 |
| F | Max2DdiameterRow LV ES | Size | -0.100 | -0.052 | 0.046 | 0.022 | 5.952E-01 | 1.096E-01 | 6.099E-01 | 1.000E+00 |
| M | Max2DdiameterRow RV ED | Size | -0.192 | -0.082 | -0.044 | -0.046 | 3.371E-07 | 6.821E-04 | 1.000E+00 | 1.000E+00 |
| F | Max2DdiameterRow RV ED | Size | -0.102 | -0.079 | -0.024 | -0.062 | 7.499E-01 | 7.288E-05 | 1.000E+00 | 1.000E+00 |
| M | Max2DdiameterRow RV ES | Size | -0.112 | -0.077 | -0.055 | 0.021 | 1.490E-01 | 4.418E-03 | 4.751E-01 | 1.000E+00 |
| F | Max2DdiameterRow RV ES | Size | -0.011 | -0.065 | -0.030 | 0.006 | 1.000E+00 | 3.680E-02 | 1.000E+00 | 1.000E+00 |
| M | Max2DdiameterSlice LV ED | Size | -0.234 | -0.046 | 0.027 | -0.075 | 7.390E-12 | 1.000E+00 | 1.000E+00 | 1.000E+00 |
| F | Max2DdiameterSlice LV ED | Size | -0.071 | -0.056 | 0.040 | -0.029 | 1.000E+00 | 3.602E-01 | 1.000E+00 | 1.000E+00 |
| M | Max2DdiameterSlice LV ES | Size | -0.172 | -0.023 | 0.032 | -0.062 | 1.193E-05 | 1.000E+00 | 1.000E+00 | 1.000E+00 |
| F | Max2DdiameterSlice LV ES | Size | -0.123 | -0.020 | 0.057 | -0.009 | 1.408E-01 | 1.000E+00 | 1.171E-01 | 1.000E+00 |
| M | Max2DdiameterSlice RV ED | Size | -0.324 | -0.187 | -0.143 | 0.018 | 5.856E-19 | 4.979E-21 | 3.384E-12 | 1.000E+00 |
| F | Max2DdiameterSlice RV ED | Size | -0.178 | -0.168 | -0.094 | -0.013 | 2.503E-04 | 7.166E-22 | 4.185E-06 | 1.000E+00 |
| M | Max2DdiameterSlice RV ES | Size | -0.172 | -0.140 | -0.069 | 0.087 | 1.426E-04 | 4.257E-11 | 5.701E-02 | 1.000E+00 |
| F | Max2DdiameterSlice RV ES | Size | -0.035 | -0.068 | -0.036 | 0.045 | 1.000E+00 | 7.306E-03 | 1.000E+00 | 1.000E+00 |
| M | Max3Ddiameter LV ED | Size | -0.265 | -0.080 | 0.035 | -0.055 | 4.721E-18 | 8.767E-05 | 1.000E+00 | 1.000E+00 |
| F | Max3Ddiameter LV ED | Size | -0.181 | -0.044 | 0.034 | -0.004 | 1.058E-05 | 7.665E-01 | 1.000E+00 | 1.000E+00 |
| M | Max3Ddiameter LV ES | Size | -0.224 | -0.060 | 0.065 | -0.054 | 8.445E-12 | 5.313E-02 | 1.294E-02 | 1.000E+00 |
| F | Max3Ddiameter LV ES | Size | -0.108 | -0.031 | 0.058 | 0.015 | 3.211E-01 | 1.000E+00 | 3.672E-02 | 1.000E+00 |
| M | Max3Ddiameter RV ED | Size | -0.269 | -0.136 | -0.030 | -0.029 | 6.211E-18 | 4.591E-15 | 1.000E+00 | 1.000E+00 |
| F | Max3Ddiameter RV ED | Size | -0.138 | -0.101 | -0.025 | -0.003 | 3.137E-03 | 3.743E-10 | 1.000E+00 | 1.000E+00 |
| M | Max3Ddiameter RV ES | Size | -0.201 | -0.111 | 0.015 | 0.024 | 2.144E-09 | 1.381E-09 | 1.000E+00 | 1.000E+00 |
| F | Max3Ddiameter RV ES | Size | -0.015 | -0.059 | -0.002 | 0.075 | 1.000E+00 | 1.308E-02 | 1.000E+00 | 1.000E+00 |
| M | MinorAxis LV ED | Size | -0.272 | -0.116 | -0.017 | -0.077 | 3.898E-16 | 2.240E-09 | 1.000E+00 | 1.000E+00 |
| F | MinorAxis LV ED | Size | -0.174 | -0.112 | 0.012 | -0.032 | 1.103E-05 | 1.441E-12 | 1.000E+00 | 1.000E+00 |
| M | MinorAxis LV ES | Size | -0.185 | -0.076 | -0.025 | -0.039 | 4.685E-07 | 1.477E-03 | 1.000E+00 | 1.000E+00 |
| F | MinorAxis LV ES | Size | -0.104 | -0.076 | 0.001 | 0.010 | 3.116E-01 | 4.980E-05 | 1.000E+00 | 1.000E+00 |
| M | MinorAxis RV ED | Size | -0.371 | -0.148 | -0.072 | -0.092 | 2.079E-29 | 4.566E-15 | 5.441E-03 | 1.000E+00 |
| F | MinorAxis RV ED | Size | -0.218 | -0.118 | -0.030 | -0.142 | 1.063E-07 | 1.475E-11 | 1.000E+00 | 5.219E-02 |
| M | MinorAxis RV ES | Size | -0.258 | -0.115 | -0.078 | -0.075 | 6.880E-14 | 8.073E-09 | 1.014E-03 | 1.000E+00 |
| F | MinorAxis RV ES | Size | -0.115 | -0.078 | -0.031 | -0.119 | 1.560E-01 | 6.287E-05 | 1.000E+00 | 3.203E-01 |
| M | RunLengthNonUniformity glrlm MYO ED | Size | -0.316 | -0.058 | 0.037 | 0.010 | 3.291E-14 | 1.000E+00 | 1.000E+00 | 1.000E+00 |
| F | RunLengthNonUniformity glrlm MYO ED | Size | -0.196 | -0.084 | 0.067 | 0.038 | 5.738E-07 | 3.315E-06 | 1.889E-03 | 1.000E+00 |
| M | RunLengthNonUniformity glrlm MYO ES | Size | -0.223 | -0.067 | 0.025 | 0.032 | 4.737E-06 | 4.627E-01 | 1.000E+00 | 1.000E+00 |
| F | RunLengthNonUniformity glrlm MYO ES | Size | -0.146 | -0.074 | 0.053 | 0.043 | 2.068E-03 | 1.481E-04 | 1.044E-01 | 1.000E+00 |
| M | SizeZoneNonUniformity glszm MYO ED | Size | -0.319 | -0.071 | 0.024 | -0.108 | 4.862E-14 | 2.171E-01 | 1.000E+00 | 1.000E+00 |
| F | SizeZoneNonUniformity glszm MYO ED | Size | -0.163 | -0.080 | -0.009 | -0.071 | 7.999E-04 | 1.156E-04 | 1.000E+00 | 1.000E+00 |
| M | SizeZoneNonUniformity glszm MYO ES | Size | 0.079 | 0.072 | 0.072 | 0.069 | 1.000E+00 | 1.387E-01 | 1.087E-01 | 1.000E+00 |
| F | SizeZoneNonUniformity glszm MYO ES | Size | 0.078 | 0.003 | 0.054 | 0.093 | 1.000E+00 | 1.000E+00 | 2.059E-01 | 1.000E+00 |
| M | SurfaceArea LV ED | Size | -0.331 | -0.105 | 0.042 | -0.031 | 1.828E-29 | 2.997E-09 | 1.000E+00 | 1.000E+00 |
| F | SurfaceArea LV ED | Size | -0.176 | -0.082 | 0.060 | 0.007 | 1.335E-10 | 2.041E-11 | 1.242E-05 | 1.000E+00 |
| M | SurfaceArea LV ES | Size | -0.230 | -0.098 | -0.009 | -0.020 | 3.909E-12 | 6.433E-07 | 1.000E+00 | 1.000E+00 |
| F | SurfaceArea LV ES | Size | -0.105 | -0.080 | 0.023 | 0.026 | 1.934E-02 | 6.658E-09 | 1.000E+00 | 1.000E+00 |
| M | SurfaceArea RV ED | Size | -0.396 | -0.167 | -0.062 | -0.100 | 2.707E-42 | 1.447E-24 | 1.147E-02 | 5.812E-01 |
| F | SurfaceArea RV ED | Size | -0.205 | -0.137 | -0.028 | -0.075 | 7.502E-13 | 5.520E-29 | 1.000E+00 | 1.000E+00 |
| M | SurfaceArea RV ES | Size | -0.274 | -0.141 | -0.109 | -0.052 | 5.609E-20 | 2.523E-17 | 2.308E-10 | 1.000E+00 |
| F | SurfaceArea RV ES | Size | -0.106 | -0.108 | -0.060 | -0.034 | 1.065E-02 | 8.783E-18 | 9.775E-05 | 1.000E+00 |
| M | Volume LV ED | Size | -0.358 | -0.157 | -0.002 | -0.051 | 8.158E-31 | 2.697E-19 | 1.000E+00 | 1.000E+00 |
| F | Volume LV ED | Size | -0.233 | -0.111 | 0.027 | -0.032 | 2.046E-19 | 1.236E-21 | 1.000E+00 | 1.000E+00 |
| M | Volume LV ES | Size | -0.223 | -0.145 | -0.058 | -0.015 | 1.781E-09 | 1.439E-13 | 2.182E-01 | 1.000E+00 |
| F | Volume LV ES | Size | -0.129 | -0.099 | -0.014 | 0.013 | 1.671E-04 | 1.313E-14 | 1.000E+00 | 1.000E+00 |
| M | Volume RV ED | Size | -0.392 | -0.143 | -0.048 | -0.140 | 1.464E-41 | 4.478E-18 | 3.960E-01 | 4.969E-03 |
| F | Volume RV ED | Size | -0.185 | -0.104 | -0.017 | -0.090 | 9.115E-13 | 4.660E-20 | 1.000E+00 | 1.198E-01 |
| M | Volume RV ES | Size | -0.267 | -0.126 | -0.118 | -0.064 | 5.323E-17 | 2.251E-12 | 5.068E-11 | 1.000E+00 |
| F | Volume RV ES | Size | -0.107 | -0.092 | -0.060 | -0.057 | 2.383E-03 | 8.192E-15 | 1.127E-05 | 1.000E+00 |
