# Supplementary material for: Cardiac Magnetic Resonance Radiomics Reveal Differential Impact of Sex, Age, and Vascular Risk Factors on Cardiac Structure and Myocardial Tissue
Source: Front Cardiovasc Med. 2021 Dec 22;8:763361. doi: 10.3389/fcvm.2021.763361 (PMC8727756; doi:10.3389/fcvm.2021.763361)
Supplement: Supplementary file 2 [file Data_Sheet_2.docx]

**Supplementary Figure 1. Flow chart of participant selection for the study**

UK Biobank participants with CMR data available

n= 32,068

Exclusion of participants with cardiovascular disease or VRF n=17,166

Exclusion of participants with cardiovascular disease

n=3,528

VRF matched subset

n=27,400

Healthy subset

n=14,902

Participants with at least one VRF n=13,700

Healthy participants n=13,700

Matched 1:1 on age and sex

*Supplementary Figure 1. CMR: cardiovascular magnetic resonance, VRF: vascular risk factor*

**Supplementary Figure 2. Interaction terms for sex-differential age related associations with radiomics features in the healthy subset grouped into clusters**

**

*Supplementary Figure 2. Results are beta coefficient corresponding to an age-sex interaction term in linear regression models additionally adjusted for age, sex, and body surface area. The y axis is standardised beta coefficients. Each dot represents association with a radiomic feature from a separate model. Black dots indicate statistically significant associations. Grey dots indicate non-significant associations. Statistical significance is based on Bonferroni adjusted p-value <0.05. Feature associations are grouped into previously defined clusters (Figure 1, Table 1). The dark line in the box plot indicates the median beta coefficient in the cluster, the box borders indicate limits of the interquartile range.*

****Supplementary Figure 3. Interaction terms for sex and age differential associations of diabetes, high cholesterol, hypertension, and smoking with radiomics features**

*Supplementary Figure 3. Results are beta coefficient corresponding to an age-sex interaction term in linear regression models additionally adjusted for age, sex, and body surface area. The y axis is standardised beta coefficients. Each dot represents association with a radiomic feature from a separate model. Black dots indicate statistically significant associations. Grey dots indicate non-significant associations. Statistical significance is based on Bonferroni adjusted p-value <0.05. Feature associations are grouped into previously defined clusters (Figure 1, Table 1). The dark line in the box plot indicates the median beta coefficient in the cluster, the box borders indicate limits of the interquartile range.*

 **Supplementary Figure 4. Associations of diabetes, high cholesterol, hypertension, and smoking with radiomics features grouped into clusters, separated by sex**

*Supplementary Figure 4. Results are from linear regression models adjusted for age, body surface area, diabetes, high cholesterol, hypertension, and smoking. Models are run separately for men and women. The y axis is standardised beta coefficients for associations of vascular risk factors (diabetes, high cholesterol, hypertension, smoking) with radiomics features. Each dot represents point estimate of association with a radiomic feature from a separate model. Black dots indicate statistically significant associations. Grey dots indicate non-significant associations. Statistical significance is based on Bonferroni adjusted p-value <0.05. Feature associations are grouped into previously defined clusters (Figure 1, Table 1). The dark line in the box plot indicates the median beta coefficient in the cluster, the box borders indicate limits of the interquartile range*
